# Construction of diazepine-containing spiroindolines via annulation reaction of α-halogenated *N*-acylhydrazones and isatin-derived MBH carbonates

**DOI:** 10.3762/bjoc.19.143

**Published:** 2023-12-18

**Authors:** Xing Liu, Wenjing Shi, Jing Sun, Chao-Guo Yan

**Affiliations:** 1 College of Chemistry & Chemical Engineering, Yangzhou University, Jiangsu, Yangzhou 225002, China

**Keywords:** acylhydrazone, annulation, azepine, MBH carbonate, spirooxindole

## Abstract

A straightforward synthetic protocol for the efficient construction of diazepine-containing spiroindolines has been developed and proceeds through a by base-promoted annulation reaction of α-halogenated *N*-acylhydrazones and isatin-derived MBH carbonates. The reaction mechanism of this formal [4 + 3] annulation includes the in situ generated allylic ylide, nucleophilic substitution, Michael additon, and elimination processes. Additionally, the similar reaction with α-halogenated *N*-tosylhydrazones also afforded *N*-tosyl-substituted spiro[indoline-3,5'-[1,2]diazepine] in satisfactory yields. This protocol provides a convenient approach for the assembly of diverse highly functionalized spiro[indoline-3,5'-[1,2]diazepines] and also features a broad substrate scope, simple reaction conditions, and high molecular convergence.

## Introduction

Among the various N-containing heterocyclic compounds, 1,2-diazepine represents one of the important privileged structural motif, which is frequently found in natural products, bioactive molecules, and pharmaceuticals [1–4]. 1,2-Diazepine derivatives exhibit promising biological activities such as anticonvulsant, antibacterial, and antiproliferative effects [5–13]. Thus, the development of alternative synthetic methodologies for functionalized 1,2-diazepines has drawn extensive attention [14–21]. One of the most attractive strategies to synthesize the 1,2-diazepine motif represents the [4 + 3] cycloaddition reaction between activated azoalkenes and 1,3-dipolarophiles [22–27].

**Scheme 1:**
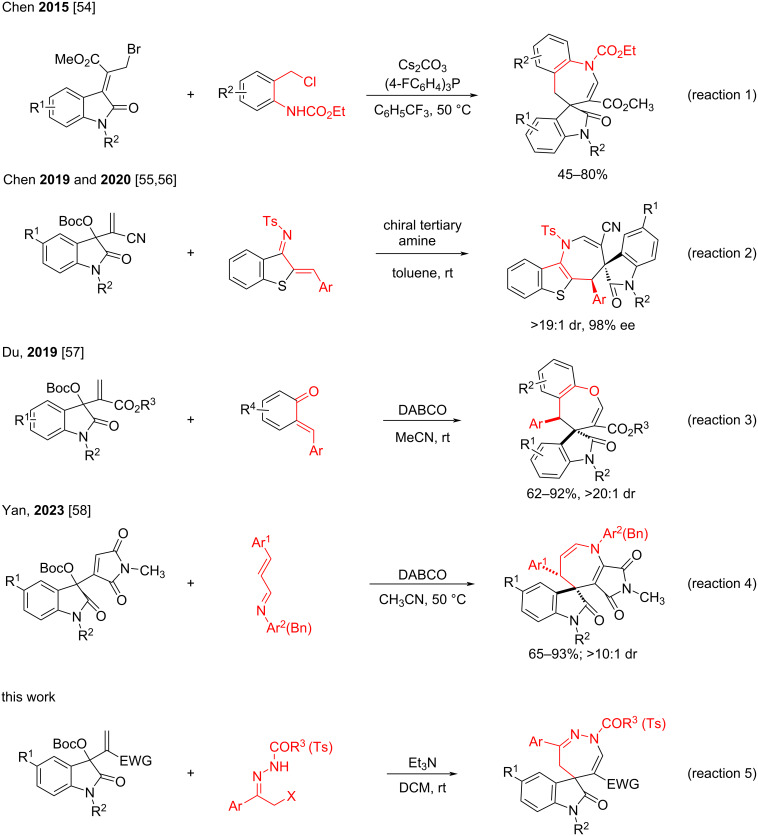
Representative [4 + 3] cycloaddition reactions of MBH carbonates derived from isatins.

In addition, spirooxindole is also a privileged structural scaffold, which has been recognized as key structural unit in many bioactive natural products and pharmaceuticals with broad biological activities [28–30]. The development of elegant synthetic methodologies for the synthesis of spirooxindole derivatives continues to be a highly active subject in organic synthesis [31–35]. In recent years, the readily available isatin-derived Morita–Baylis–Hillman (MBH) carbonates have become one of the most powerful reagents for the construction of diverse spirooxindoles [36–43]. In the presence of Lewis bases, isatin-derived MBH carbonates usually undergo [3 + 2] and [3 + 3] cycloaddition reactions with a broad range of active C–C and C–N double bonds and 1,3-dipolarpohiles to give various five- or six-membered cyclic spirooxindoles [44–48]. However, the [4 + 3] cycloaddition reaction of isatin-derived MBH carbonates with active diene components has not been well developed probably due to the lack of suitable active diene compounds [49–53]. In this respect, Chen and co-workers were the first who reported the efficient synthesis of spiro[azepine-4,3'-indoline] derivatives via the [4 + 3] cycloaddition reaction of bromo-substituted isatin-derived MBH adducts and *N*-(*o*-chloromethyl)arylamides. In this efficient protocol, the reactive allylic phosphonium ylides and aza-*o*-quinone methides were generated in situ and sequentially underwent a [4 + 3] cycloaddition reaction (reaction 1 in Scheme 1) [54]. Recently, Chen and co-workers reported a chiral tertiary amine-catalyzed asymmetric γ-regioselective [4 + 3] annulation reaction of isatin-derived MBH carbonates and cyclic 2-benzylidenebenzo[*b*]thiophen-3-ylidene)benzenesulfonamides to give chiral azepane spirooxindoles with excellent stereoselectivity (reaction 2 in Scheme 1) [55–56]. Du and co-workers reported a DABCO-mediated [4 + 3] cycloaddition reaction between *o-*quinone methides and isatin-derived MBH carbonates to give functionalized benzo[*b*]oxepine derivatives in satisfactory yields and with good diastereoselectivity (reaction 3, Scheme 1) [57]. Very recently, we found that the base-catalyzed [4 + 3] cycloaddition reaction of isatin-derived MBH carbonates with various α,β-unsaturated *N*-arylaldimines affords spiro[indoline-3,5'-pyrrolo[3,4-*b*]azepine] derivatives (reaction 4 in Scheme 1) [58]. Inspired by these elegant synthetic protocols and in continuation of our aim to provide efficient synthetic protocols for diverse spirooxindoles [59–67], we herein wish to report the base-mediated formal [4 + 3] annulation reaction of isatin-derived MBH carbonates with α-halogenated *N*-acylhydrazones for the convenient synthesis of functionalized spiro[indoline-3,5'-[1,2]diazepine] derivatives (reaction 5 in Scheme 1).

## Results and Discussion

Initially, the reaction conditions were screened by employing α-chloro-*N*-benzoylhydrazone **1a** and MBH nitrile of isatin **2a** as standard reaction. The main experiments are briefly summarized in Table 1. At first, the reaction in DCM in the presence of common organic bases such as DMAP, DABCO, or DBU gave the expected spiro[indoline-3,5'-[1,2]diazepine] **3a** in low to moderate yields (Table 1, entries 1–3). However, piperidine, TMG, and Na_2_CO_3_ failed to promote the annulation reaction (Table 1, entries 4–6). When triethylamine was employed as the base, the reaction gave the spiro compound **3a** in 71% yield (Table 1, entry 7). The yields of product **3a** remained nearly unchanged when the reaction time was either shortened to 12 h or prolonged to 36 h (Table 1, entries 8 and 9). Also, neither decreasing or increasing the reaction temperature did improve the yield of product **3a** which was obtained in 26% yield at 0 °C and 52% yield in refluxing DCM, respectively (Table 1, entries 10 and 11). In the presence of triethylamine, the reaction in other solvents such as DCE, THF, and CHCl_3_ gave the product **3a** in 61%, 46% and 56% yields, respectively (Table 1, entries 12–14). However, the reaction did not proceed in toluene and acetonitrile (Table 1, entries 15 and 16). When lowering the amount of triethylamine to one equivalent, the yield of spiro compound **3a** decreased to 35% (Table 1, entry 17). At last, if the amount of α-halogenated acylhydrazone was reduced, the yield of product **3a** also decreased to 57% yield (Table 1, entry 18). Thus, the best reaction conditions were carrying out the reaction in DCM at room temperature for 24 hours in the presence of an excess amount of triethylamine.

**Table 1:** Optimizing the reaction conditions.^a^

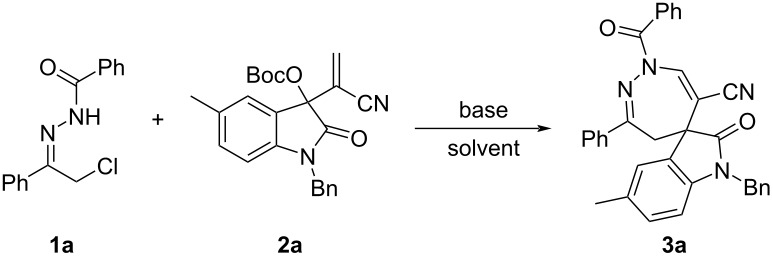					

Entry	Base	Solvent	Time (h)	Temp. (°C)	Yield of **3a** (%)^b^

1	DMAP	DCM	24	rt	45
2	DABCO	DCM	24	rt	33
3	DBU	DCM	24	rt	15
4	piperidine	DCM	12	rt	0
5	TMG	DCM	12	rt	0
6	Na_2_CO_3_	DCM	12	rt	0
7	Et_3_N	DCM	24	rt	71
8	Et_3_N	DCM	12	rt	72
9	Et_3_N	DCM	36	rt	72
10	Et_3_N	DCM	12	0	26
11	Et_3_N	DCM	12	reflux	52
12	Et_3_N	DCE	12	rt	61
13	Et_3_N	THF	12	rt	42
14	Et_3_N	CHCl_3_	12	rt	56
15	Et_3_N	MeCN	12	rt	0
16	Et_3_N	PhMe	12	rt	0
17	Et_3_N^c^	DCM	12	rt	35
18	Et_3_N^d^	DCM	12	rt	57

^a^Reaction conditions: α-halogenated acylhydrazone **1a** (0.2 mmol), MBH nitrile of isatin **2a** (0.1 mmol), base (0.2 mmol), solvent (4.0 mL); ^b^Isolated yields. ^c^Et_3_N (0.1 mmol); ^d^α-halogenated acylhydrazone (0.12 mmol).

**Scheme 2:**
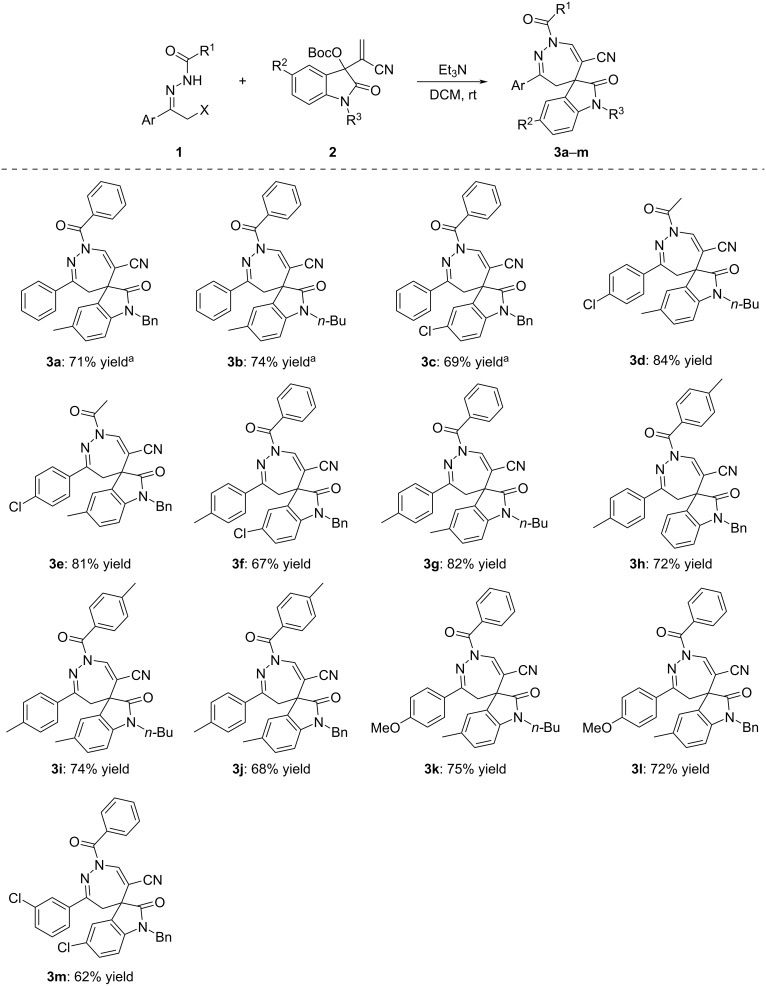
Synthesis of spiro[indoline-3,5'-[1,2]diazepines] **3a**–**m**. Conditions: α-halogenated acylhydrazone (0.2 mmol), MBH nitrile of isatin (0.1 mmol), Et_3_N (0.2 mmol), DCM (4.0 mL), rt, 24 h; ^a^the α-chloro-*N*-acylhydrazone was used, for other compounds α-bromo-*N*-acylhydrazones were used. Yields refer to isolated compounds.

With the optimized reaction conditions in hands, we next examined the scope of the reaction by employing various functionalized substrates and the results are summarized in Scheme 2. As it can be seen, the expected dihydrospiro[indoline-3,5'-[1,2]diazepines] **3a**–**m** were obtained in reasonable to good yields. Both, α-chloro- and α-bromo-*N*-acylhydrazones could be successfully used in the reaction and gave similar results. Also, hydrazones with different benzoyl-protecting groups were well tolerated in the reaction. In general, α-bromo-*N*-acetylhydrazones gave higher yields than the corresponding α-bromo-*N*-benzoylhydrazones. Also, substituents present in the MBH nitriles of isatins showed marginal effects on the yields. The obtained spiro[indoline-3,5'-[1,2]diazepines] **3a**–**m** were fully characterized by various spectroscopic methods. Because there are one C=C bond and one C=N bond in the molecules **3a**–**m**, no diastereoisomers are obtained. Therefore, the ^1^H NMR spectra gave simple absorptions for the characteristic groups in the molecules.

**Scheme 3:**
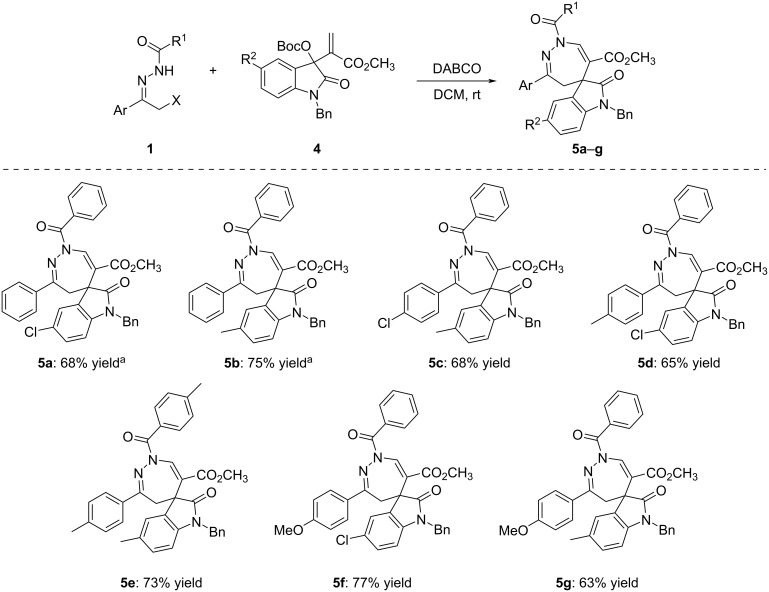
Synthesis of spiro[indoline-3,5'-[1,2]diazepines] **5a**–**g**. Conditions: α-halogenated acylhydrazone (0.2 mmol), MBH ester of isatin (0.1 mmol), DABCO (0.2 mmol), DCM (4.0 mL), rt, 24 h; ^a^α-chloro-*N*-acylhydrazone was used, for other compounds α-bromo-*N-*acylhydrazone was used. Yields refer to isolated compounds.

For further developing the scope of the [4 + 3] cycloaddition reaction, MBH esters of isatins **4** were also employed in the reaction, but it was found that the reaction proceeded sluggishly in the presence of triethylamine (Scheme 3). However, the expected annulation reaction proceeded smoothly in dichloromethane within 24 hours in the presence of DABCO as base, affording the corresponding spiro[indoline-3,5'-[1,2]diazepine]-6'-carboxylates **5a**–**g** in 63–77% yields (Scheme 3). The substituents on both substrates also showed little effect on the yields. The chemical structures were fully characterized by HRMS, IR, ^1^H and ^13^C NMR spectra.

**Scheme 4:**
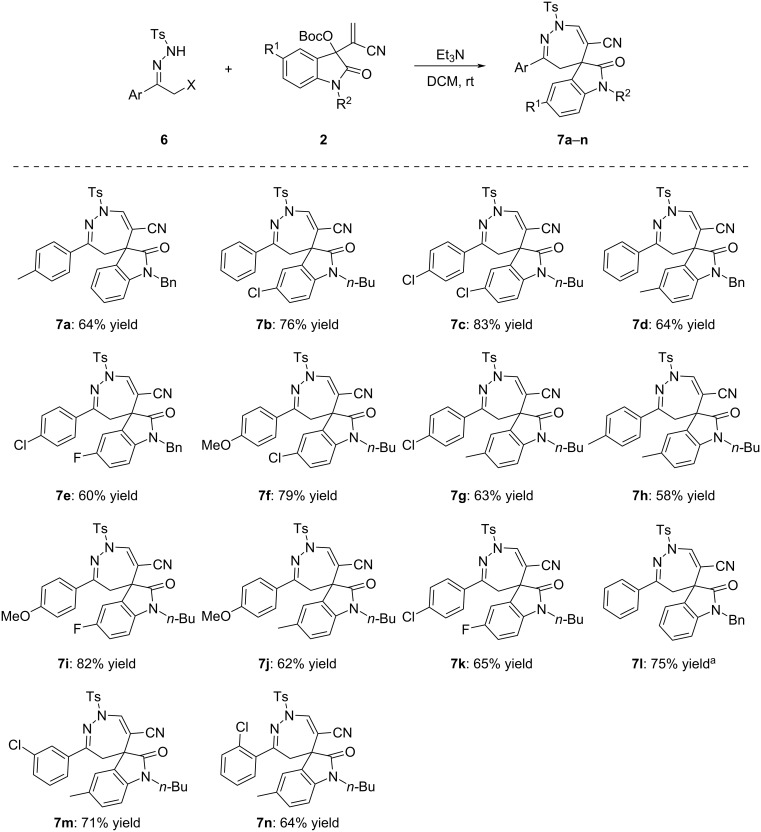
Synthesis of dihydrospiro[indoline-3,5'-[1,2]diazepines] **7a**–**n**. Conditions: α-halogenated *N*-tosylhydrazone (0.2 mmol), MBH nitrile of isatin (0.2 mmol), Et_3_N (0.4 mmol), DCM (4.0 mL), rt, 3 h; ^a^α-chloro-*N*-tosylhydrazone was used, for other compounds α-bromo-*N*-tosylhydrazone was used. Yields refer to isolated compounds.

**Figure 1:**
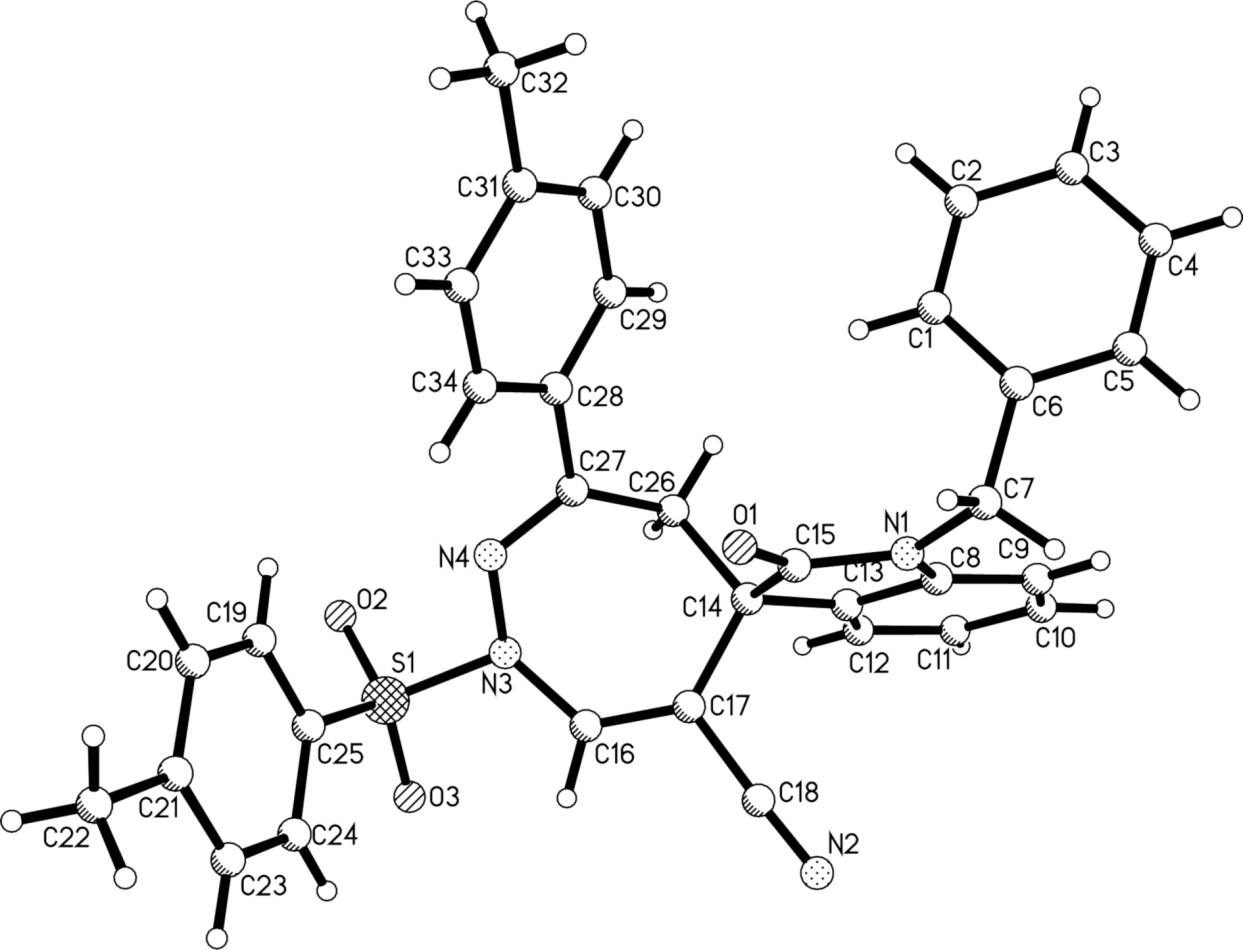
Single crystal structure of the spiro compound **7a**.

For demonstrating the synthetic value of this protocol, α-halogenated *p*-toluenesulfonylhydrazones **6** were also used in the reaction and the results are summarized in Scheme 4. As it can be seen, the triethylamine-mediated [4 + 3] cycloaddition could be accomplished at room temperature in two hours. These results clearly showed that the α-halogenated *p*-toluenesulfonylhydrazones **6** have a much higher reactivity compared to that of α-halogenated benzoylsulfonylhydrazones **1** in this reaction. The desired spiro[indoline-3,5'-[1,2]diazepines] **7a**–**n** were obtained in satisfactory yields of 58–83% and the substituents in MBH nitriles of isatins showed only marginal effects on the yields. The chemical structures of the spiro compounds **7a**–**n** were established by various spectroscopy methods. In addition, the single crystal structure of compound **7a** was also determined by X-ray diffraction (Figure 1). As can be seen from Figure 1, both the C–C and C–N double bonds are part of the cyclic 1,2-diazepine ring and the methylene unit is connected to the 3-positon of the oxindole moiety.

**Scheme 5:**
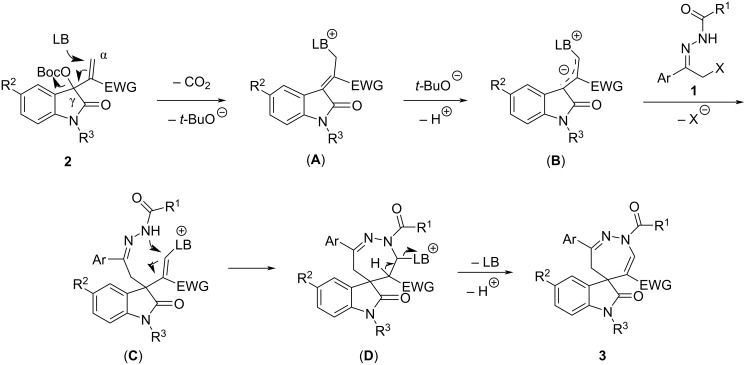
Proposed reaction mechanism.

On the basis of the current results and previous works [54–61], a reaction mechanism for the formation of the spiro[indoline-3,5'-[1,2]diazepines] has been proposed and is depicted in Scheme 5. At first, MBH carbonates of isatin **2** is attacked at the α-position by the Lewis base to give the ammonium salt **A** with elimination of carbon dioxide and a *tert*-butoxide ion. Secondly, the ammonium salt **A** is deprotonated by the in situ generated *tert*-butoxide ion to give the allylic ylide **B**. Thirdly, the intermediate **C** is formed by the nucleophilic substitution of a halide ion in substrate **1** by the allylic ylide **B**. Then, Michael addition of the amino group to the C=C bond results in the cyclic intermediate **D**. Finally, the spiro[indoline-3,5'-[1,2]diazepine] **3** is produced by the elimination of a proton and the Lewis base. Obviously, the spiro compounds **5** and **7** are formed by a similar reaction mechanism.

**Scheme 6:**
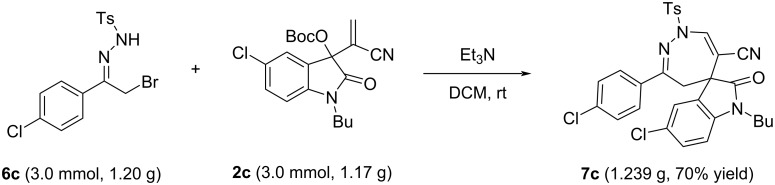
Gram-scale synthesis of compound **7c**.

Additionally, the method was applied to a gram-scale reaction of α-halogenated *p*-toluenesulfonylhydrazone **6c** and MBH nitrile of isatin **2c** under the standard conditions (Scheme 6). The expected spiro product **7c** was successfully obtained in 70% yield, which clearly demonstrated that this base-promoted annulation reaction is applicable for the large-scale synthesis of diazepine-containing spiroindolines.

## Conclusion

In summary, we have developed a synthetic protocol for the base-mediated annulation reaction of α-halogenated acylhydrazones with isatin-derived MBH carbonates. The reaction provides a straightforward synthetic route for the efficient construction of novel spiro[indoline-3,5'-[1,2]diazepine] derivatives in satisfactory yields. The advantages of this reaction include the use of readily available reagents, mild conditions, satisfactory yields, broad substrate scope, high molecular convergence, and atomic economy. The synthetic applications of this annulation reaction in heterocyclic chemistry might be significant.

## Experimental

**General procedure for the preparation of dihydrospiro[indoline-3,5'-[1,2]diazepines] 3a–l:** A 10 mL reaction tube was charged with α-halogenated acylhydrazone (0.2 mmol), MBH nitrile of isatin (0.1 mmol), triethylamine (0.2 mmol) and dichloromethane (4.0 mL) and the mixture was stirred at room temperature for 24 hours. After removing the solvent by rotatory evaporation at reduced pressure, the residue was subjected to column chromatography with ethyl acetate, dichloromethane and petroleum ether 1:3:7 (v/v/v) to give pure product for analysis.

**1'-Benzoyl-1-benzyl-5-methyl-2-oxo-3'-phenyl-1',4'-dihydrospiro[indoline-3,5'-[1,2]diazepine]-6'-carbonitrile (3a)**: yellow solid, 0.370 g, 71%; mp 186–187 °C; ^1^H NMR (400 MHz, CDCl_3_) δ 8.61 (s, 1H, ArH), 7.75–7.72 (m, 2H, ArH), 7.57–7.53 (m, 1H, ArH), 7.47–7.43 (m, 2H, ArH), 7.35–7.31 (m, 2H, ArH), 7.30–7.27 (m, 5H, ArH), 7.26–7.23 (m, 1H, ArH), 7.22–7.18 (m, 2H, ArH), 7.05–7.02 (m, 1H, ArH), 6.96 (s, 1H, ArH), 6.73 (d, *J* = 8.0 Hz, 1H, ArH), 4.95–4.90 (m, 2H, CH_2_), 3.47 (d, *J* = 14.0 Hz, 1H, CH), 3.24 (d, *J* = 14.0 Hz, 1H, CH), 2.16 (s, 3H, CH_3_) ppm; ^13^C NMR (100 MHz, CDCl_3_) δ 173.8, 170.9, 159.6, 139.6, 138.4, 136.2, 135.2, 133.3, 133.2, 131.8, 130.7, 130.3, 130.0, 129.2, 128.9, 128.4, 127.9, 127.8, 127.4, 127.3, 125.5, 117.4, 109.9, 95.0, 52.3, 44.4, 37.0, 20.9 ppm; IR (KBr) ν: 2960, 2936, 2870, 2211, 1717, 1626, 1498, 1445, 1367, 1268, 1193, 1112, 1090, 1009, 903, 868, 815 cm^−1^; HRMS–ESI TOF (*m*/*z*): [M + Na]^+^ calcd for C_34_H_26_N_4_O_2_Na, 545.1956; found, 545.1948.

**General procedure for the preparation of dihydrospiro[indoline-3,5'-[1,2]diazepines] 5a–i:** A 10 mL reaction tube was charged with α-halogenated acylhydrazone (0.2 mmol), MBH ester of isatin (0.1 mmol), DABCO (0.2 mmol, 0.0224 g), and dichloromethane (4.0 mL) and the mixture was stirred at room temperature for 24 hours. After removing the solvent by rotatory evaporation at reduced pressure, the residue was subjected to column chromatography with ethyl acetate, dichloromethane and petroleum ether 1:3:7 (v/v/v) to give pure product for analysis.

**Methyl 1'-benzoyl-1-benzyl-5-chloro-2-oxo-3'-phenyl-1',4'-dihydrospiro[indoline-3,5'-[1,2]diazepine]-6'-carboxylate (5a)**: yellow solid, 0.391 g, 68%; mp 189–191 °C; ^1^H NMR (400 MHz, CDCl_3_) δ 9.19 (s, 1H, ArH), 7.73–7.71 (m, 2H, ArH), 7.56–7.53 (m, 1H, ArH), 7.48–7.42 (m, 4H, ArH), 7.38–7.35 (m, 2H, ArH), 7.32–7.31 (m, 1H, ArH), 7.29–7.27 (m, 1H, ArH), 7.14 (t, *J* = 8.0 Hz, 2H, ArH), 7.09–7.05 (m, 3H, ArH), 6.86–6.85 (m, 1H, ArH), 6.70 (d, *J* = 8.4 Hz, 1H, ArH), 5.02 (s, 2H, CH_2_), 3.65 (s, 3H, OCH_3_), 3.49 (d, *J* = 13.6 Hz, 1H, CH), 3.10 (d, *J* = 13.6 Hz, 1H, CH) ppm; ^13^C NMR (100 MHz, CDCl_3_) δ 176.3, 171.3, 165.8, 160.6, 141.1, 137.3, 135.9, 135.5, 133.8, 131.6, 130.6, 129.7, 128.9, 128.5, 128.4, 127.9, 127.8, 127.5, 127.0, 124.4, 111.6, 110.4, 52.2, 51.1, 44.4, 36.8 ppm; IR (KBr) ν: 2924, 2853, 1721, 1608, 1484, 1456, 1430, 1340, 1170, 812 cm^−1^; HRMS–ESI TOF (*m*/*z*): [M + H]^+^ calcd for C_34_H_27_ClN_3_O_4_, 576.1685; found, 576.1683.

**General procedure for the preparation of dihydrospiro[indoline-3,5'-[1,2]diazepines] 7a–l:** A 10 mL reaction tube was charged with α-halogenated *N*-tosylhydrazone (0.2 mmol), MBH ester of isatin (0.2 mmol), triethylamine (0.4 mmol, 0.0364 g), and dichloromethane (4.0 mL) and the mixture was stirred at room temperature for three hours. After removing the solvent by rotatory evaporation at reduced pressure, the residue was subjected to column chromatography with ethyl acetate, dichloromethane, and petroleum ether 1:3.7 (v/v/v) to give pure product for analysis.

**1-Benzyl-2-oxo-3'-(*p*-tolyl)-1'-tosyl-1',4'-dihydrospiro[indoline-3,5'-[1,2]diazepine]-6'-carbonitrile (7a):** white solid, 64%; mp 226–230 °C; ^1^H NMR (400 MHz, CDCl_3_) δ 8.10 (s, 1H, C=CH-N), 7.97 (d, *J* = 8.0 Hz, 2H, ArH), 7.42 (d, *J* = 8.0 Hz, 2H, ArH), 7.29 (d, *J* = 8.4 Hz, 2H, ArH), 7.28–7.26 (s, 1H, ArH), 7.26–7.24 (m, 2H, ArH), 7.22–7.20 (m, 1H, ArH), 7.19–7.16 (m, 2H, ArH), 7.09 (s, 1H, ArH), 7.06 (d, *J* = 4.8 Hz, 2H, ArH), 6.98 (t, *J* = 7.6 Hz, 1H, ArH), 6.78 (d, *J* = 7.6 Hz, 1H, ArH), 4.88 (d, *J* = 15.6 Hz, 1H, CH), 4.79 (d, *J* = 15.6 Hz, 1H, CH), 3.29 (d, *J* = 10.0 Hz, 1H, CH), 3.20 (d, *J* = 10.0 Hz, 1H, CH), 2.49 (s, 3H, CH_3_), 2.34 (s, 3H, CH_3_) ppm; ^13^C {^1^H} NMR (100 MHz, CDCl_3_) δ 173.7, 160.5, 145.9, 141.8, 141.1, 139.4, 135.1, 133.5, 132.7, 129.9, 129.9, 129.5, 129.2, 129.0, 128.8, 127.8, 127.3, 127.2, 124.5, 123.5, 117.3, 109.9, 91.9, 52.1, 44.3, 37.3, 21.8, 21.3 ppm; IR (KBr) ν: 3057, 3055, 2928, 2217, 1716, 1613, 1488, 1467, 1449, 1369, 1297, 1259, 1189, 1175, 1090, 1021, 999, 869, 804, 764, 754, 696, 667, 657, 639 cm^−1^; HRMS–ESI TOF (*m*/*z*): [M + Na]^+^ calcd for C_34_H_28_ClN_4_O_3_SNa, 595.1774; found, 595.1765.

The crystallographic data of compound **7a** (CCDC 2280223) have been deposited at the Cambridge Crystallographic Database Centre.

## Supporting Information

File 1 Characterization data and ^1^H, ^13^C NMR, and HRMS spectra for all new compounds.

## Data Availability

All data that supports the findings of this study is available in the published article and/or the supporting information to this article.
