# Supplementary material for: Construction of diazepine-containing spiroindolines via annulation reaction of α-halogenated N-acylhydrazones and isatin-derived MBH carbonates
Source: Beilstein J Org Chem. 2023 Dec 18;19:1923–32. doi: 10.3762/bjoc.19.143 (PMC10760482; doi:10.3762/bjoc.19.143)
Supplement: File 1 — Characterization data and 1H, 13C NMR, and HRMS spectra for all new compounds. [file Beilstein_J_Org_Chem-19-1923-s001.pdf]

BEILSTEIN JOURNAL OF ORGANIC CHEMISTRY

Supporting Information

for

Construction of diazepine-containing spiroindolines via annulation reaction of α-halogenated *N*-acylhydrazones and isatin-derived MBH carbonates

Xing Liu, Wenjing Shi, Jing Sun and Chao-Guo Yan

Beilstein J. Org. Chem. **2023,** *19,* 1923–1932. doi:10.3762/bjoc.19.143

Characterization data and ^{1}H, ^{13}C NMR, and HRMS spectra for all new compounds

License and Terms: This is a supporting information file under the terms of the Creative Commons Attribution License (https://creativecommons.org/licenses/by/4.0). Please note that the reuse, redistribution and reproduction in particular requires that the author(s) and source are credited and that individual graphics may be subject to special legal provisions.
The license is subject to the *Beilstein Journal of Organic Chemistry* terms and conditions: (https://www.beilstein-journals.org/bjoc/terms)

1'-Benzoyl-1-benzyl-5-methyl-2-oxo-3'-phenyl-1',4'-dihydrospiro[indoline-3,5'-[1,2]diazepine]-6'-carbonitrile (3a): yellow solid, 0.370 g, 71%, m.p. 186-187 °C; ^{1}H NMR (400 MHz, $CDCl_3$) δ: 8.61 (s, 1H, ArH), 7.74 (d, *J* = 8.0 Hz, 2H, ArH), 7.57-7.53 (m, 1H, ArH), 7.47-7.43 (m, 2H, ArH), 7.35-7.31 (m, 2H, ArH), 7.30-7.27 (m, 5H, ArH), 7.26-7.23 (m, 1H, ArH), 7.22-7.18 (m, 2H, ArH), 7.05-7.02 (m, 1H, ArH), 6.96 (s, 1H, ArH), 6.73 (d, *J* = 8.0 Hz, 1H, ArH), 4.93 (s, 2H, CH_2), 3.47 (d, *J* = 14.0 Hz, 1H, CH), 3.24 (d, *J* = 14.0 Hz, 1H, CH), 2.16 (s, 3H, CH_3) ppm; ^{13}C NMR (100 MHz, $CDCl_3$) δ: 173.8, 170.9, 159.6, 139.6, 138.4, 136.2, 135.2, 133.3, 133.2, 131.8, 130.7, 130.3, 130.0, 129.2, 128.9, 128.4, 127.9, 127.8, 127.4, 127.3, 125.5, 117.4, 109.9, 95.0, 52.3, 44.4, 37.0, 20.9 ppm. IR (KBr) υ: 2960, 2936, 2870, 2211, 1717, 1626,1498, 1445, 1367, 1268, 1193, 1112, 1090, 1009, 903, 868, 815 cm^{-1}; MS (*m/z*): HRMS (ESI-TOF) Calcd. for $C_{34}H_{26}N_4O_2Na$ ($[M+Na]^+$): 545.1956, Found: 545.1948.

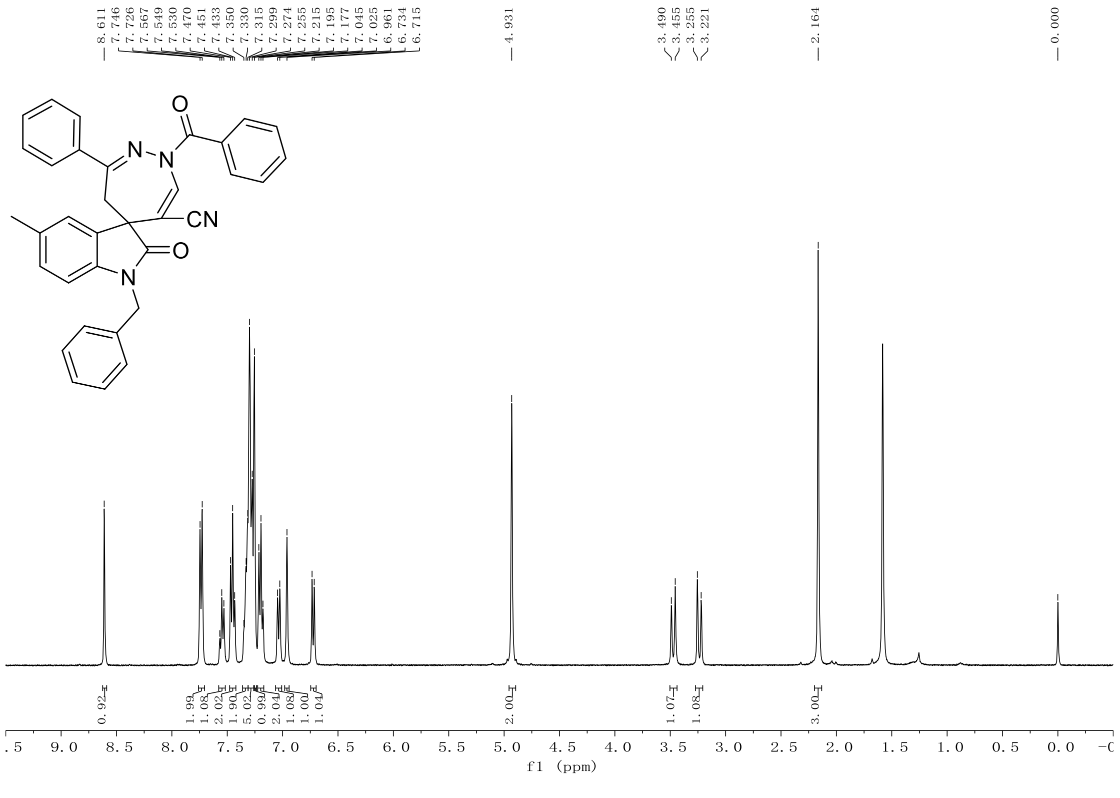

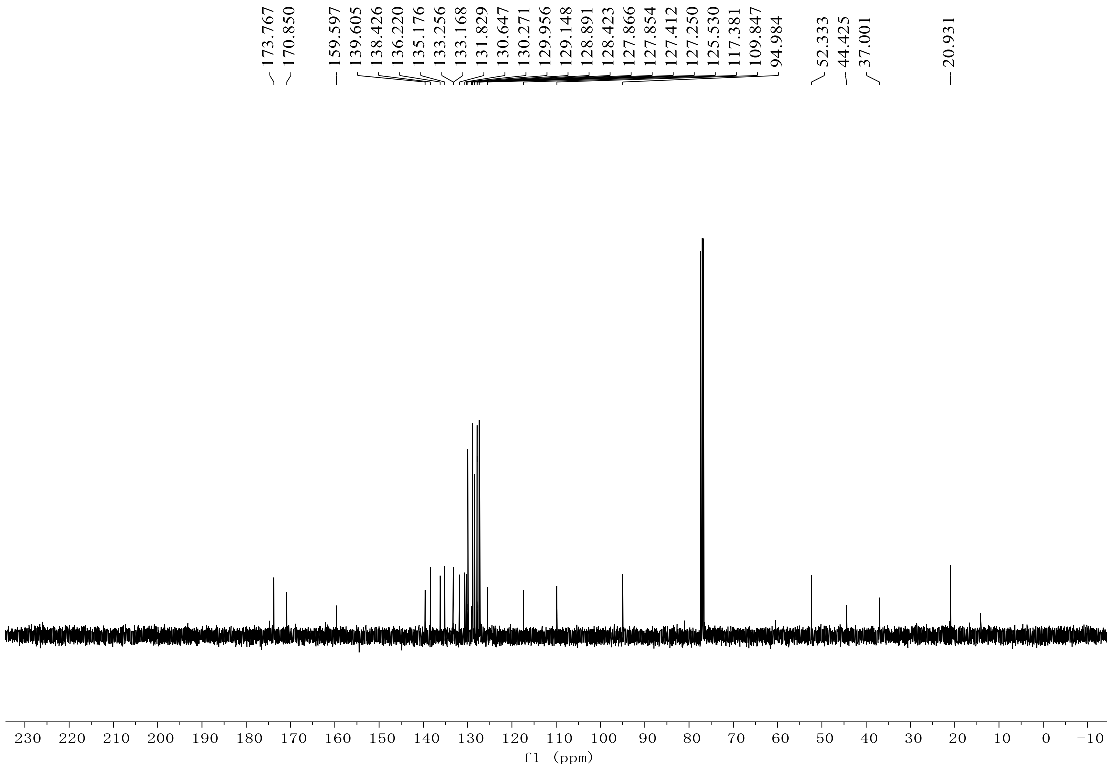
173.767
170.850
159.597
139.605
138.426
136.220
135.176
133.256
133.168
131.829
130.647
130.271
129.956
129.148
128.891
128.423
127.866
127.854
127.412
127.250
125.530
117.381
109.847
94.984
52.333
44.425
37.001
20.931
230 220 210 200 190 180 170 160 150 140 130 120 110 100 90 80 70 60 50 40 30 20 10 0 −10
f1 (ppm)

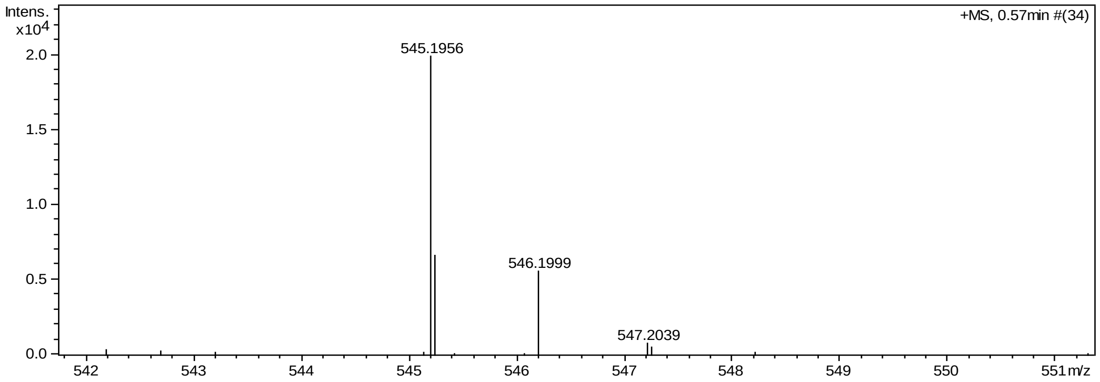
Intens.
x10^4
+MS, 0.57min #(34)
545.1956
546.1999
547.2039
2.0
1.5
1.0
0.5
0.0
542 543 544 545 546 547 548 549 550 551 m/z

1'-Benzoyl-1-butyl-5-methyl-2-oxo-3'-phenyl-1',4'-dihydrospiro[indoline-3,5'-[1,2]diazepine]-6'-carbonitrile (3b): yellow solid, 0.345 g, 74%, m.p. 191-193 ºC; ^{1}H NMR (600 MHz, $CDCl_3$) δ: 8.57 (s, 1H, ArH), 7.73 (d, *J* = 7.8 Hz, 2H, ArH), 7.55 (t, *J* = 7.2 Hz, 1H, ArH), 7.45 (t, *J* = 7.2 Hz, 2H, ArH), 7.34-7.32 (m, 1H, ArH), 7.27 (m, 1H, ArH), 7.21 (t, *J* = 7.2 Hz, 2H, ArH), 7.14 (d, *J* = 7.2 Hz, 1H, ArH), 6.94 (s, 1H, ArH), 6.85 (d, *J* = 7.8 Hz, 1H, ArH), 3.81-3.68 (m, 2H, CH_2), 3.45 (d, *J* = 14.4 Hz, 1H, CH), 3.18 (d, *J* = 13.8 Hz, 1H, CH), 2.18 (s, 3H, CH_3), 1.71-1.67 (m, 2H, CH_2), 1.40-1.36 (m, 2H, CH_2), 0.94 (t, *J* = 7.2 Hz, 3H, CH_3) ppm; ^{13}C NMR (100 MHz, $CDCl_3$) δ: 173.5, 170.8, 159.8, 140.1, 138.2, 136.3, 133.2, 132.9, 131.8, 130.6, 130.2, 129.9, 129.2, 128.4, 127.8, 127.2, 125.7, 117.1, 109.1, 95.4, 52.2, 40.4, 36.7, 29.4, 20.9, 20.0, 13.7 ppm; IR (KBr) υ: 2917, 1752, 1735, 1719, 1654, 1577, 1561, 1495, 1114, 957, 902, 855, 816 cm^{-1}; MS (*m/z*): HRMS (ESI-TOF) Calcd. for $C_{31}H_{29}N_4O_2$ ($[M+H]^+$): 489.2285, Found: 489.2304.

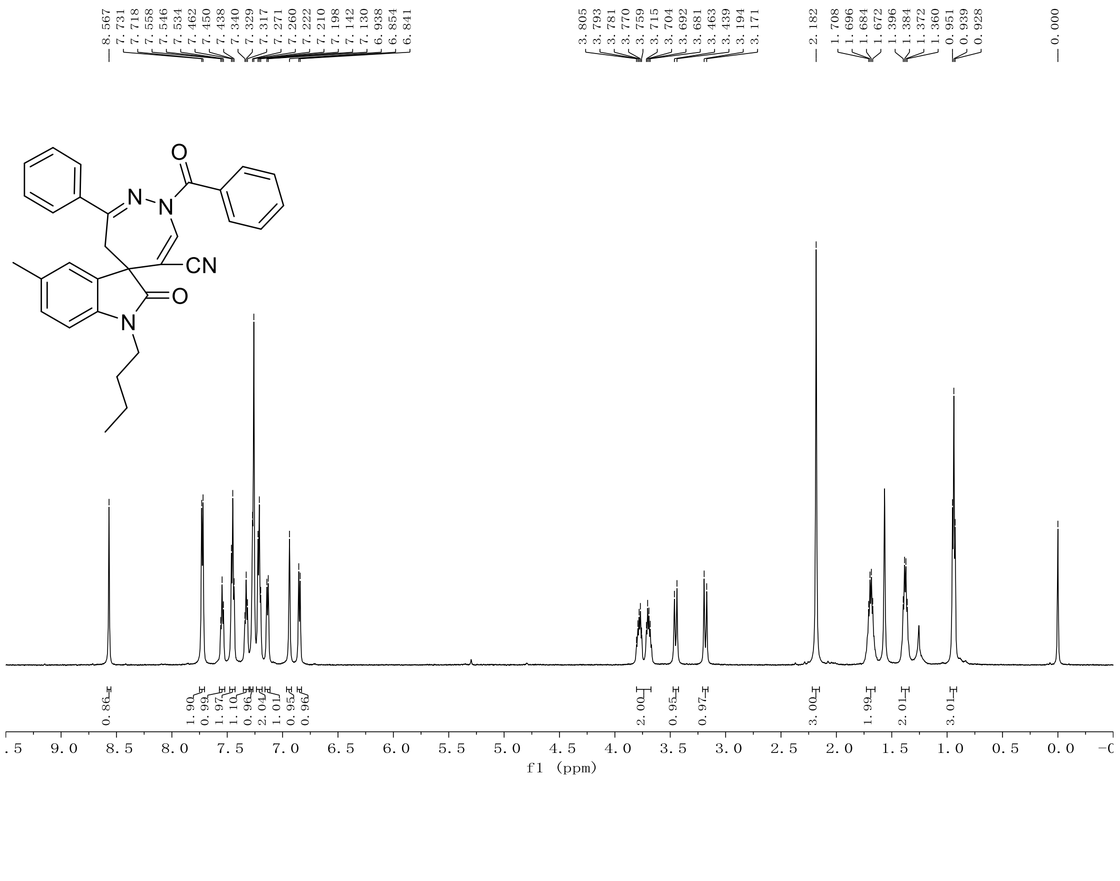

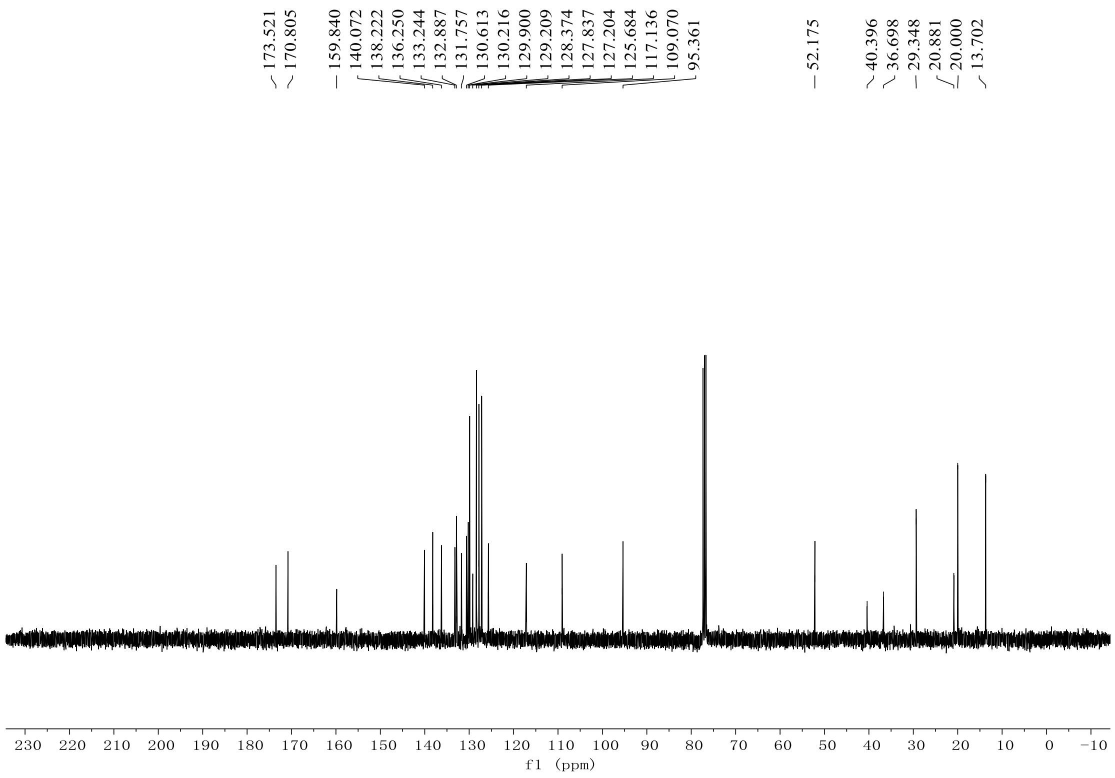

173.521
170.805
159.840
140.072
138.222
136.250
133.244
132.887
131.757
130.613
130.216
129.900
129.209
128.374
127.837
127.204
125.684
117.136
109.070
95.361
52.175
40.396
36.698
29.348
20.881
20.000
13.702
f1 (ppm)

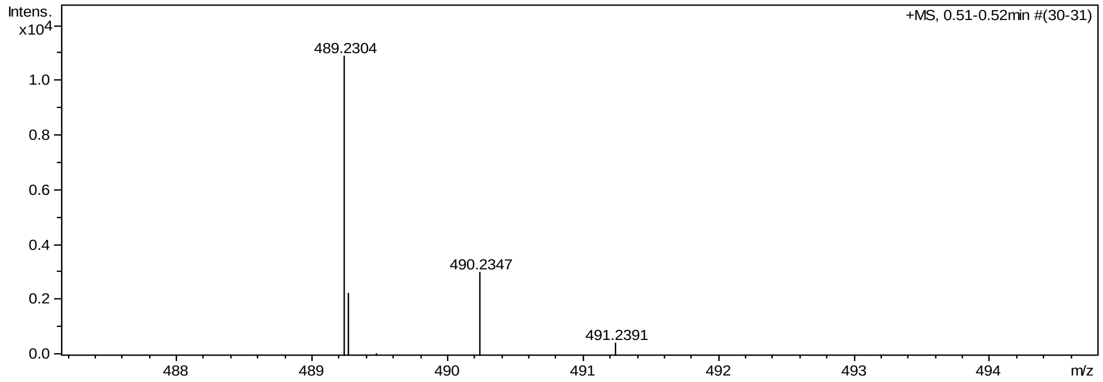

Intens.
x10^4
+MS, 0.51-0.52min #(30-31)
489.2304
490.2347
491.2391
m/z

1'-Benzoyl-1-benzyl-5-chloro-2-oxo-3'-phenyl-1',4'-dihydrospiro[indoline-3,5'-[1,2]diazepine]-6'-carbonitrile (3c): yellow solid, 0.317 g, 69%, m.p. 195-196 ºC; ^{1}H NMR (400 MHz, $CDCl_3$) δ: 8.63 (s, 1H, ArH), 7.75-7.73 (m, 2H, ArH), 7.57-7.53 (m, 1H, ArH), 7.47-7.43 (m, 2H, ArH), 7.38-7.29 (m, 5H, ArH), 7.28-7.27 (m, 3H, ArH), 7.25-7.24 (m, 2H, ArH), 7.21-7.20 (m, 2H, ArH), 7.14 (d, J = 2.4 Hz, 1H, ArH), 6.76 (d, J = 8.4 Hz, 1H, ArH), 4.95 (d, J = 15.6 Hz, 1H, CH), 4.91 (d, J = 15.6 Hz, 1H, CH), 3.47 (d, J = 13.6 Hz, 1H, CH), 3.26 (d, J = 13.6 Hz, 1H, CH) ppm; ^{13}C NMR (100 MHz, $CDCl_3$) δ: 173.4, 170.7, 158.9, 140.6, 138.7, 136.0, 134.6, 133.0, 131.9, 130.9, 130.7, 130.0, 130.0, 129.1, 129.0, 128.9, 128.9, 128.6, 128.4, 128.1, 127.9, 127.4, 127.3, 127.2, 126.8, 125.3, 117.1, 111.1, 93.8, 52.3, 36.7 ppm; IR (KBr) υ: 2959, 2928, 2871, 2215, 1712, 1632,1492, 1438, 1360, 1269, 1194, 1197, 1091, 1013, 905, 863, 811 cm^{-1}; MS (m/z): HRMS (ESI-TOF) Calcd. for $C_{26}H_{25}ClN_4O_2Na$ ($[M+Na]^+$): 483.1574, Found: 483.1558.

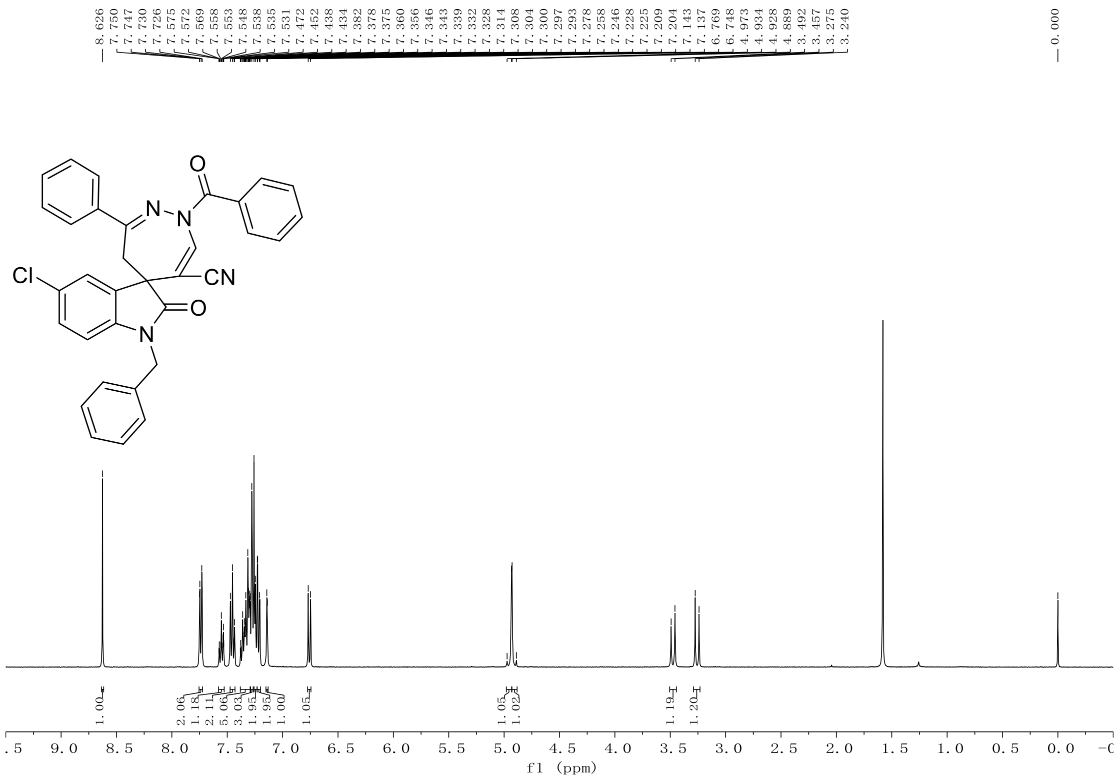

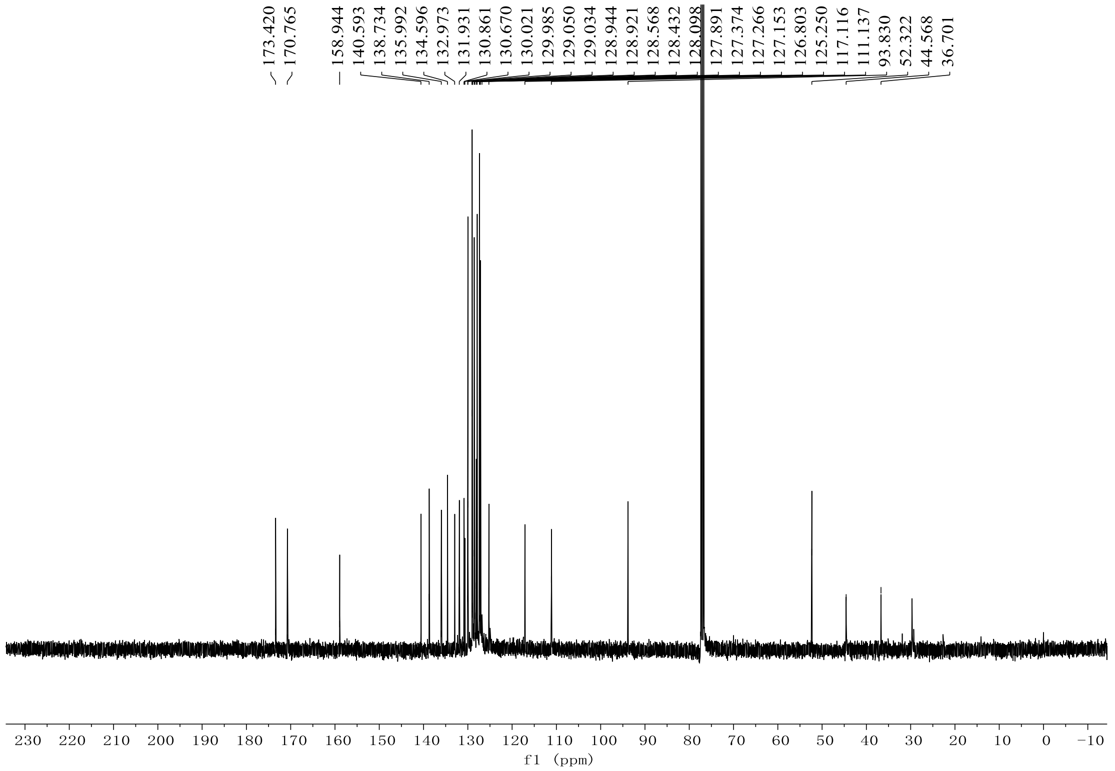
173.420
170.765
158.944
140.593
138.734
135.992
134.596
132.973
131.931
130.861
130.670
130.021
129.985
129.050
129.034
128.944
128.921
128.568
128.432
128.098
127.891
127.374
127.266
127.153
126.803
125.250
117.116
111.137
93.830
52.322
44.568
36.701
f1 (ppm)

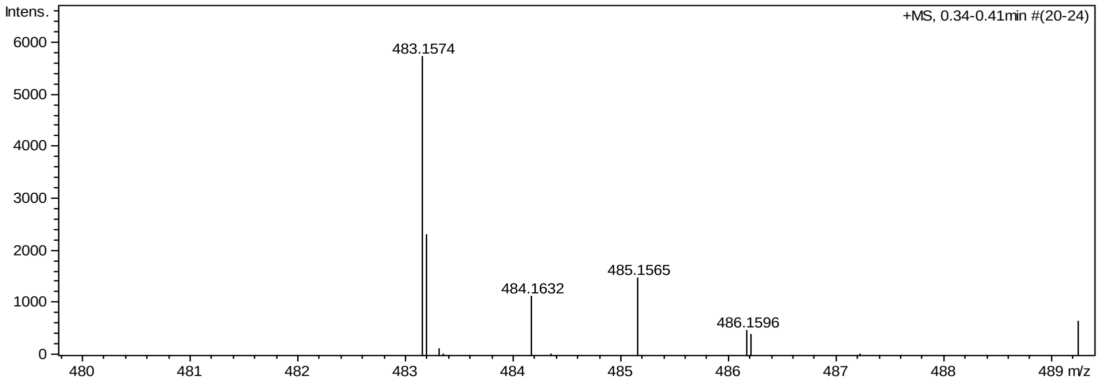
Intens.
+MS, 0.34-0.41min #(20-24)
483.1574
484.1632
485.1565
486.1596
m/z

1'-Acetyl-1-butyl-3'-(4-chlorophenyl)-5-methyl-2-oxo-1',4'-dihydrospiro[indoline-3,5'-[1,2]diazepine]-6'-carbonitrile (3d): yellow solid, 0.386 g, 84%, m.p. 132-133 °C; ^{1}H NMR (400 MHz, $CDCl_3$) δ: 8.33 (s, 1H, ArH), 7.52-7.50 (m, 2H, ArH), 7.33-7.30 (m, 2H, ArH), 7.16-7.13 (m, 1H, ArH), 6.96 (s, 1H, ArH), 6.83-6.81 (m, 1H, ArH), 3.74-3.68 (m, 1H, CH), 3.67-3.61 (m, 1H, CH), 3.23 (d, J = 14.0 Hz, 1H, CH), 3.09 (d, J = 13.6 Hz, 1H, CH), 2.55 (s, 3H, CH_3), 2.27 (s, 3H, CH_3), 1.65-1.59 (m, 2H, CH_2), 1.32-1.26 (m, 2H, CH_2), 0.91-0.87 (m, 3H, CH_3) ppm; ^{13}C NMR (100 MHz, $CDCl_3$) δ: 173.4, 173.1, 158.3, 139.8, 136.9, 135.4, 133.0, 130.3, 129.5, 128.7, 128.4, 125.2, 117.1, 109.1, 94.1, 52.3, 40.3, 36.9, 29.3, 22.4, 21.0, 19.9, 13.6 ppm; IR (KBr) υ: 2959, 2932, 2872, 2215, 1716, 1626,1495, 1443, 1364, 1268, 1197, 1111, 1093, 1012, 900, 867, 812 cm^{-1}; MS (m/z): HRMS (ESI-TOF) Calcd. for $C_{26}H_{25}ClN_4O_2Na$ ([M+Na]$^+$): 483.1558, Found: 483.1574.

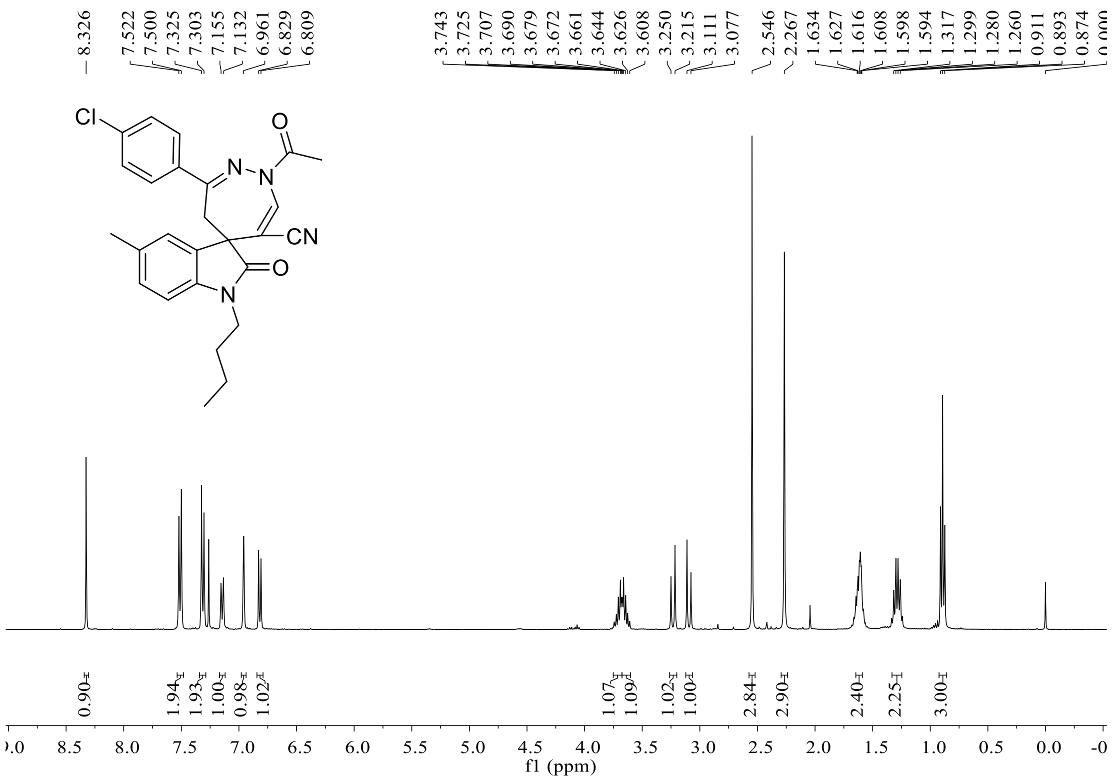

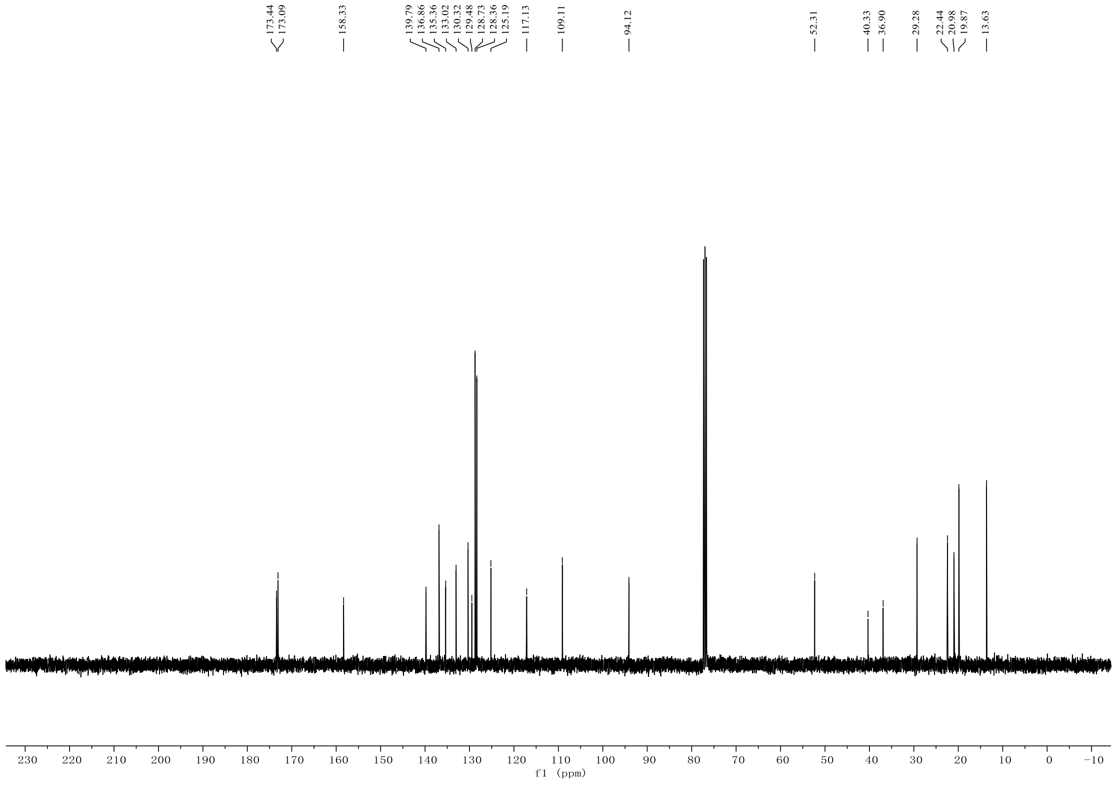
173.44
173.09
158.33
139.79
136.86
135.36
133.02
130.32
129.48
128.73
128.36
125.19
117.13
109.11
94.12
52.31
40.33
36.90
29.28
22.44
20.98
19.87
13.63
f1 (ppm)

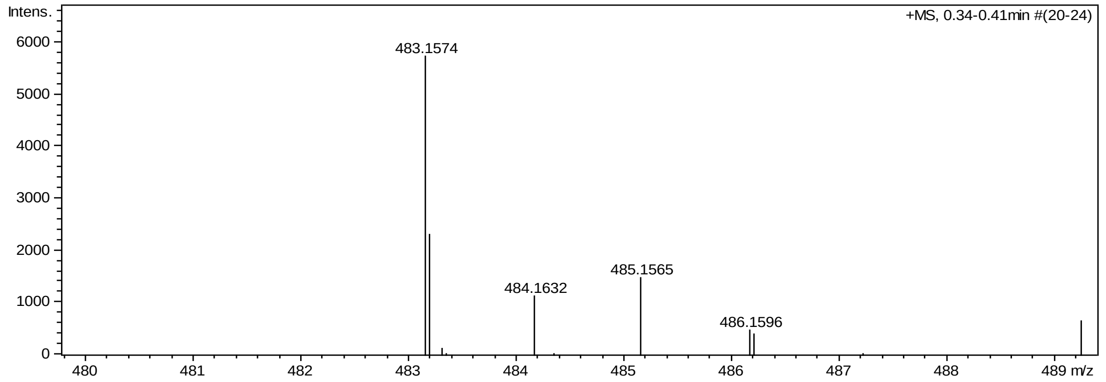
Intens.
+MS, 0.34-0.41min #(20-24)
483.1574
484.1632
485.1565
486.1596
m/z

1'-Acetyl-1-benzyl-3'-(4-chlorophenyl)-5-methyl-2-oxo-1',4'-dihydrospiro[indoline-3,5'-[1,2]diazepine]-6'-carbonitrile (3e): yellow solid, 0.400 g, 81%, m.p. 174-175 °C; ^{1}H NMR (400 MHz, $CDCl_3$) δ: 8.38 (s, 1H, ArH), 7.50 (d, *J* = 8.4 Hz, 2H, ArH), 7.31 (s, 1H, ArH), 7.30-7.26 (m, 4H, ArH), 7.21-7.18 (m, 2H, ArH), 7.06 (d, *J* = 8.0 Hz, 1H, ArH), 6.98 (s, 1H, ArH), 6.72 (d, *J* = 8.0 Hz, 1H, ArH), 4.92 (d, *J* = 15.6 Hz, 1H, CH), 4.80 (d, *J* = 15.6 Hz, 1H, CH), 3.24 (d, *J* = 14.8 Hz, 1H, CH), 3.13 (d, *J* = 14.4 Hz, 1H, CH), 2.56 (s, 3H, CH_3), 2.25 (s, 3H, CH_3) ppm; ^{13}C NMR (100 MHz, $CDCl_3$) δ: 173.7, 173.2, 158.2, 139.4, 137.1, 136.9, 135.3, 135.2, 133.4, 130.4, 129.4, 128.9, 128.8, 128.5, 128.4, 127.9, 127.4, 127.3, 125.1, 117.4, 109.8, 93.7, 52.5, 44.4, 37.3, 22.5, 21.0 ppm; IR (KBr) υ: 2923, 2853, 1716, 1666, 1602, 1496, 1456, 1364, 1268, 1170, 1093, 809 cm^{-1}; MS (*m/z*): HRMS (ESI-TOF) Calcd. for $C_{29}H_{25}ClN_4O_2Na$ ($[M+H]^+$): 495.1606, Found: 495.1582.

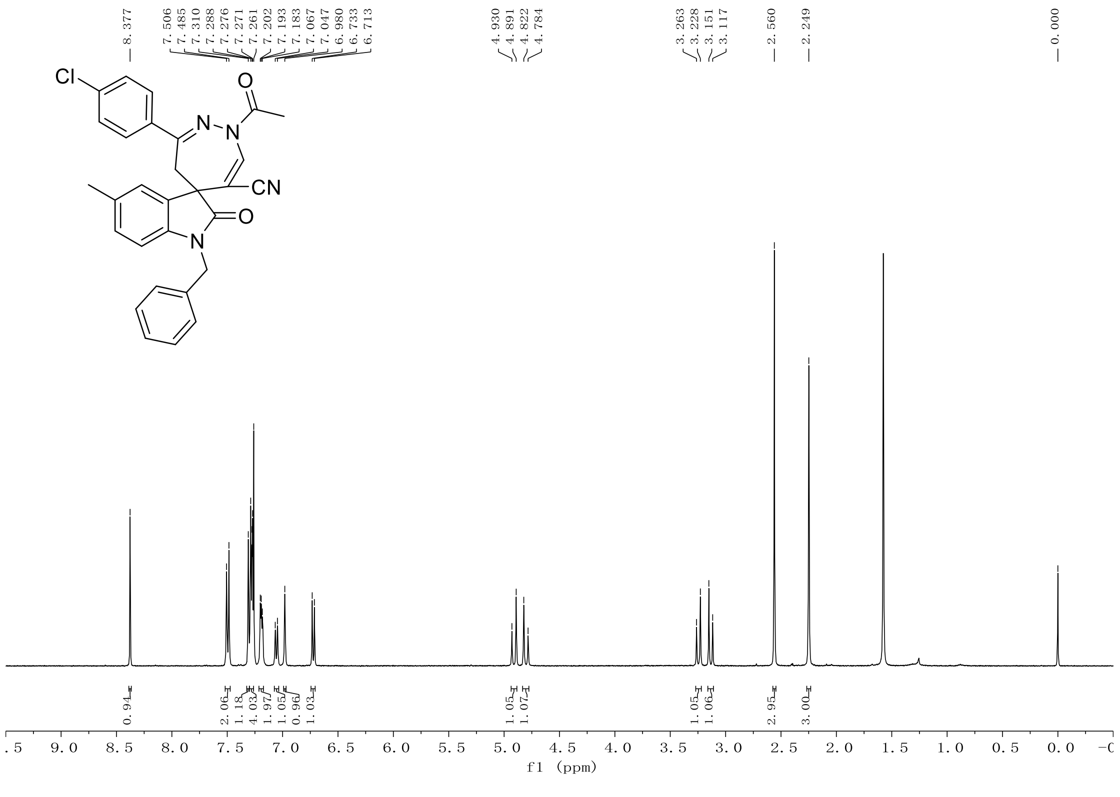

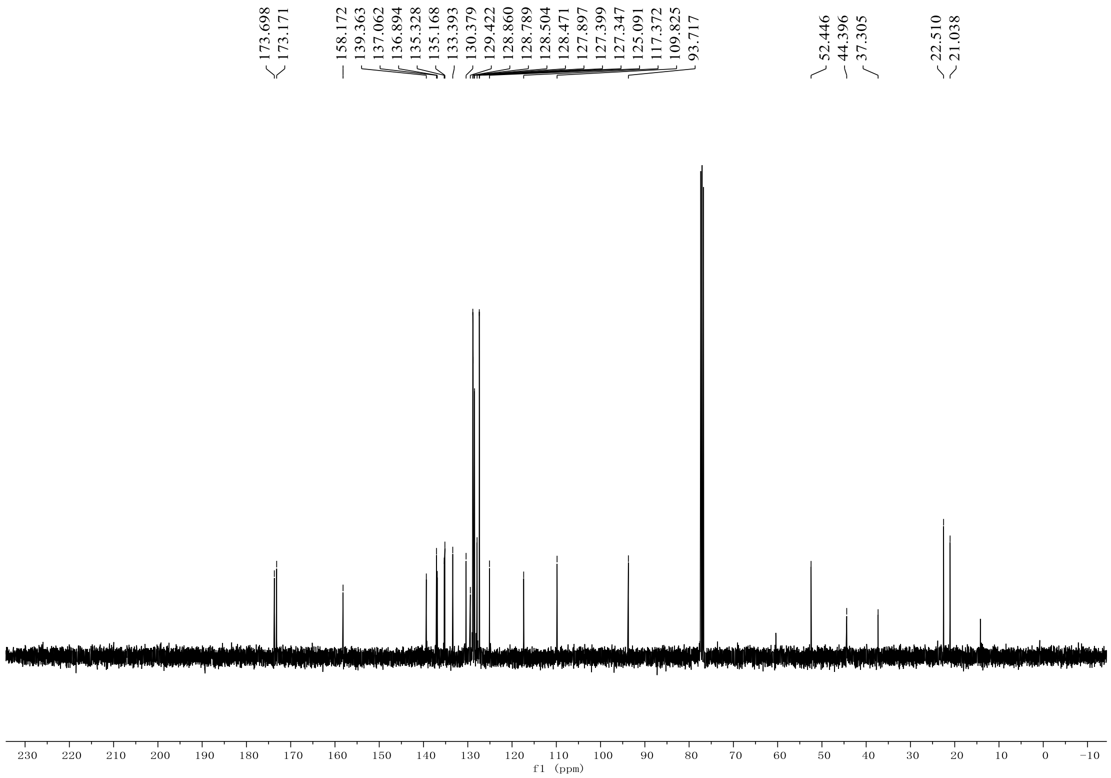
173.698
173.171
158.172
139.363
137.062
136.894
135.328
135.168
133.393
130.379
129.422
128.860
128.789
128.504
128.471
127.897
127.399
127.347
125.091
117.372
109.825
93.717
52.446
44.396
37.305
22.510
21.038
230 220 210 200 190 180 170 160 150 140 130 120 110 100 90 80 70 60 50 40 30 20 10 0 −10
f1 (ppm)

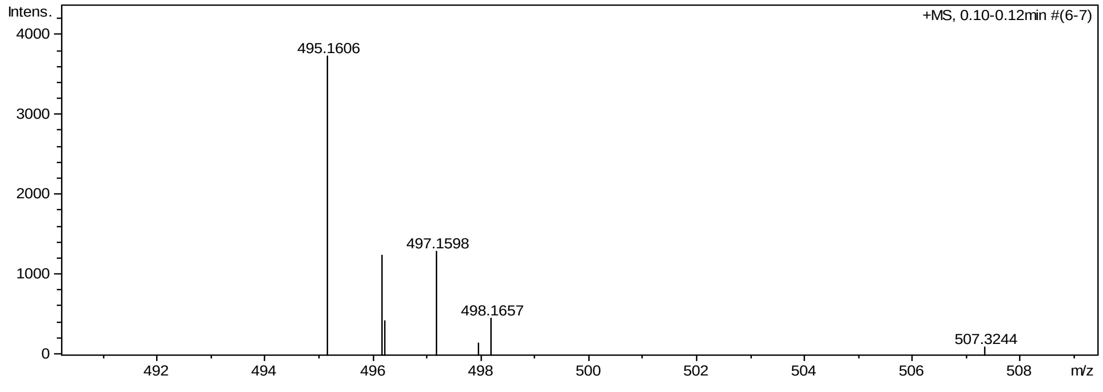
Intens.
+MS, 0.10-0.12min #(6-7)
495.1606
497.1598
498.1657
507.3244
0
1000
2000
3000
4000
492 494 496 498 500 502 504 506 508 m/z

1'-Benzoyl-1-benzyl-5-chloro-2-oxo-3'-(p-tolyl)-1',4'-dihydrospiro[indoline-3,5'-[1,2]diazepine]-6'-carbonitrile (3f): yellow solid, 0.372 g, 67%, m.p. 213-214 ºC; ^{1}H NMR (400 MHz, $CDCl_3$) δ: 8.62 (s, 1H, ArH), 7.74-7.72 (m, 2H, ArH), 7.56-7.53 (m, 1H, ArH), 7.46-7.43 (m, 2H, ArH), 7.35-7.30 (m, 3H, ArH), 7.28-7.26 (m, 2H, ArH), 7.22-7.19 (m, 2H, ArH), 7.17-7.13 (m, 3H, ArH), 7.04-7.02 (d, J = 8.4 Hz, 2H, ArH), 6.74 (m, J = 8.4 Hz, 1H, ArH), 4.96 (d, J = 15.6 Hz, 1H, CH), 4.91 (d, J = 15.2 Hz, 1H, CH), 3.47 (d, J = 14.0 Hz, 1H, CH), 3.23 (d, J = 14.0 Hz, 1H, CH), 2.31 (s, 3H, CH_3) ppm; ^{13}C NMR (100 MHz, $CDCl_3$) δ: 173.4, 170.7, 159.1, 141.4, 140.6, 138.9, 134.6, 133.2, 133.0, 131.8, 130.7, 130.0, 130.0, 129.3, 129.0, 128.1, 127.8, 127.4, 127.1, 125.3, 117.1, 111.1, 93.8, 52.3, 36.5, 21.3 ppm; IR (KBr) υ: 2943, 2879, 2197, 1867, 1791, 1698, 1568, 1488, 1374, 1339, 1280, 1171, 968, 902, 846, 809 cm^{-1}; MS (m/z): HRMS (ESI-TOF) Calcd. for $C_{34}H_{25}ClN_4O_2Na$ ($[M+Na]^+$): 579.1570, Found: 579.1558.

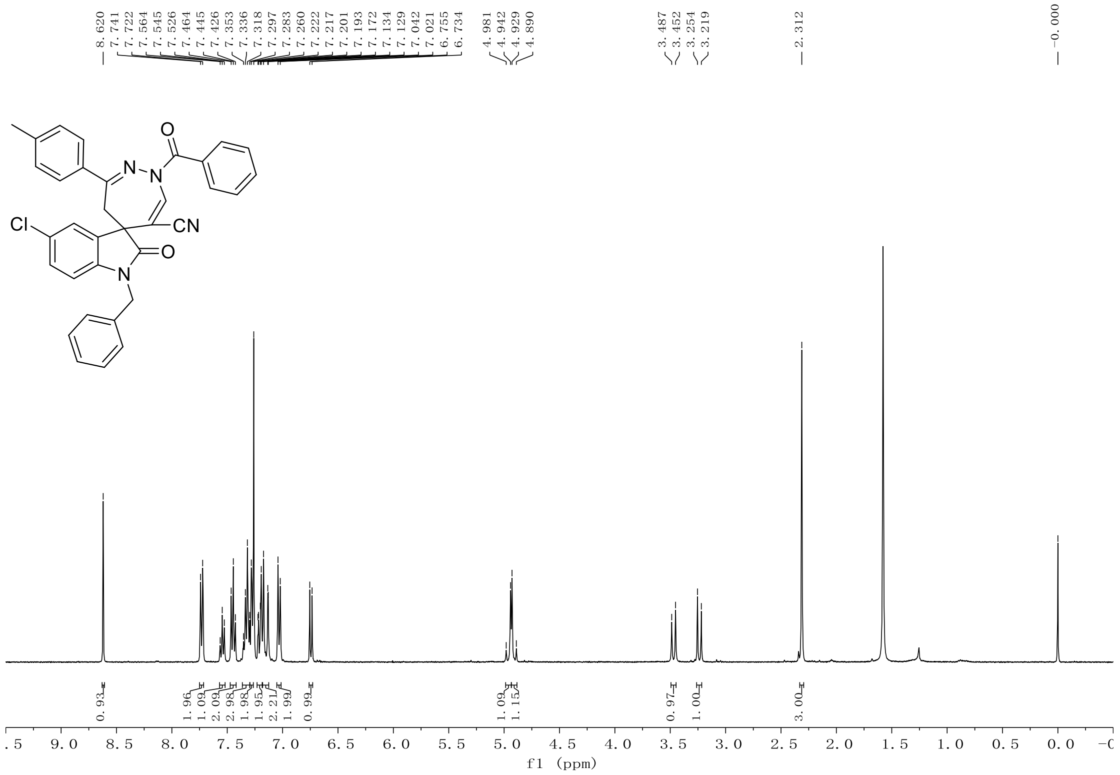

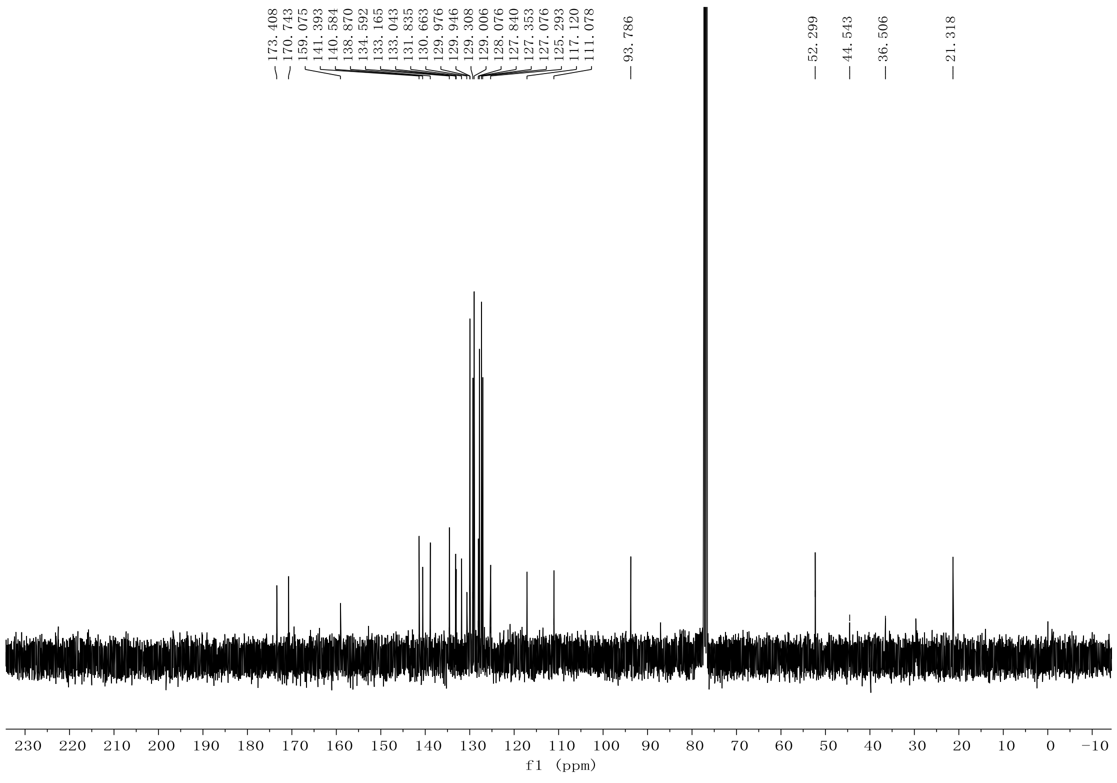
173.408
170.743
159.075
141.393
140.584
138.870
134.592
133.165
133.043
131.835
130.663
129.976
129.946
129.308
129.006
128.076
127.840
127.353
127.076
125.293
117.120
111.078
93.786
52.299
44.543
36.506
21.318
230 220 210 200 190 180 170 160 150 140 130 120 110 100 90 80 70 60 50 40 30 20 10 0 −10
f1 (ppm)

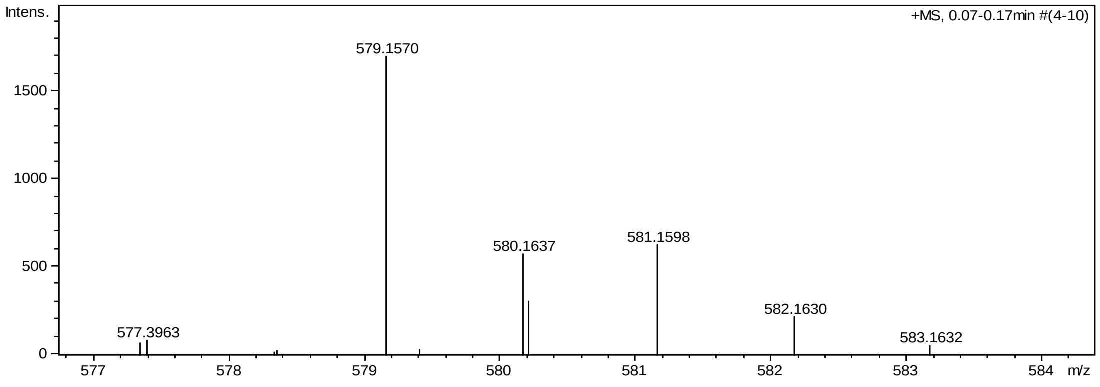
Intens.
+MS, 0.07-0.17min #(4-10)
579.1570
580.1637
581.1598
582.1630
583.1632
577.3963
1500
1000
500
0
577
578
579
580
581
582
583
584 m/z

1'-Benzoyl-1-butyl-5-methyl-2-oxo-3'-(p-tolyl)-1',4'-dihydrospiro[indoline-3,5'-[1,2]diazepine]-6'-carbonitrile (3g): yellow solid, 0.412 g, 82%, m.p. 131-133 ºC; ^{1}H NMR (600 MHz, $CDCl_3$) δ: 8.56 (s, 1H, ArH), 7.71 (d, J = 7.2 Hz, 2H, ArH), 7.55-7.52 (m, 1H, ArH), 7.45-7.42 (m, 2H, ArH), 7.16 (d, J = 7.8 Hz, 2H, ArH), 7.13 (d, J = 8.4 Hz, 1H, ArH), 7.01 (d, J = 7.8 Hz, 2H, ArH), 6.94 (s, 1H, ArH), 6.84 (d, J = 7.2 Hz, 1H, ArH), 3.80-3.75 (m, 1H, CH), 3.71-3.66 (m, 1H, CH), 3.43 (d, J = 13.8 Hz, 1H, CH), 3.16 (d, J = 13.8 Hz, 1H, CH), 2.29 (s, 3H, CH_3), 2.18 (s, 3H, CH_3), 1.71-1.66 (m, 2H, CH_2), 1.40-1.36 (m, 2H, CH_2), 0.94 (t, J = 7.2 Hz, 3H, CH_3) ppm; ^{13}C NMR (100 MHz, $CDCl_3$) δ: 173.5, 170.8, 160.0, 141.1, 140.0, 138.4, 133.4, 133.3, 132.8, 131.7, 130.2, 129.9, 129.1, 127.8, 127.1, 125.7, 117.2, 109.0, 95.3, 52.2, 40.4, 36.5, 29.3, 21.3, 20.9, 20.0, 13.7 ppm; IR (KBr) υ: 2930, 2871, 2211, 1715, 1625, 1600, 1495, 1446, 1348, 1279, 1189, 1113, 1062, 1027, 958, 900, 857, 815 cm^{-1}; MS (m/z): HRMS (ESI-TOF) Calcd. for $C_{32}H_{31}N_4O_2$ ($[M+H]^+$): 503.2460, Found: 503.2442.

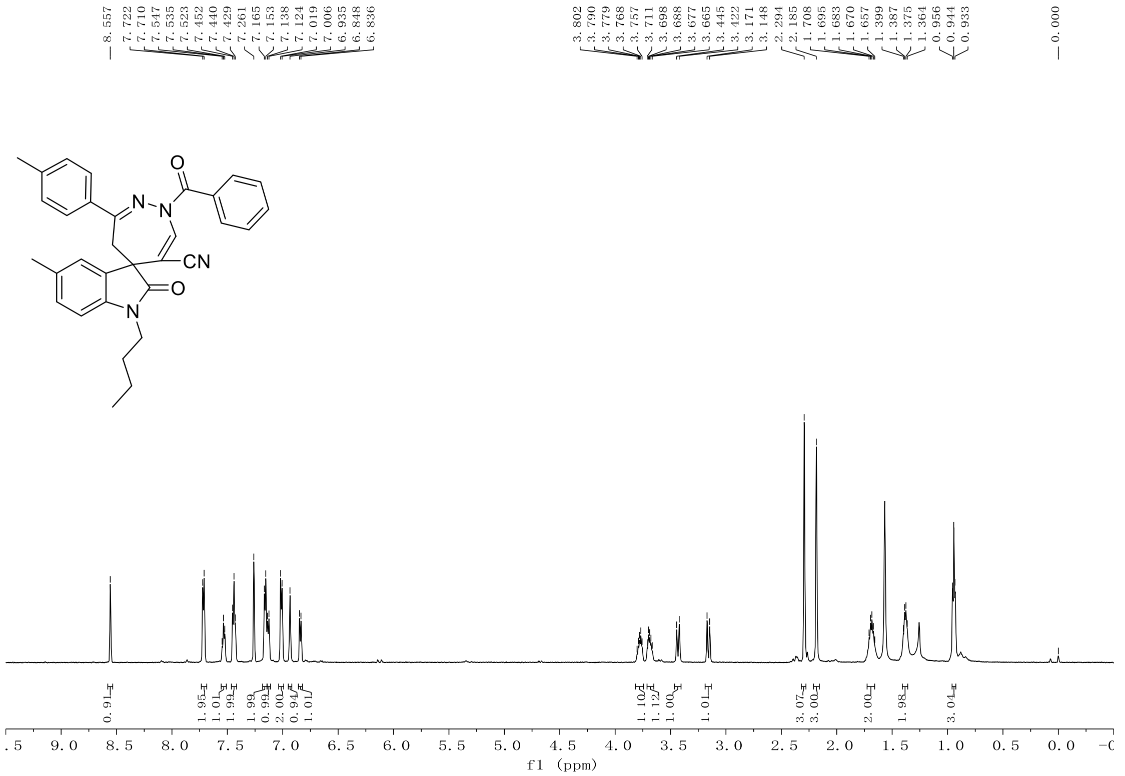

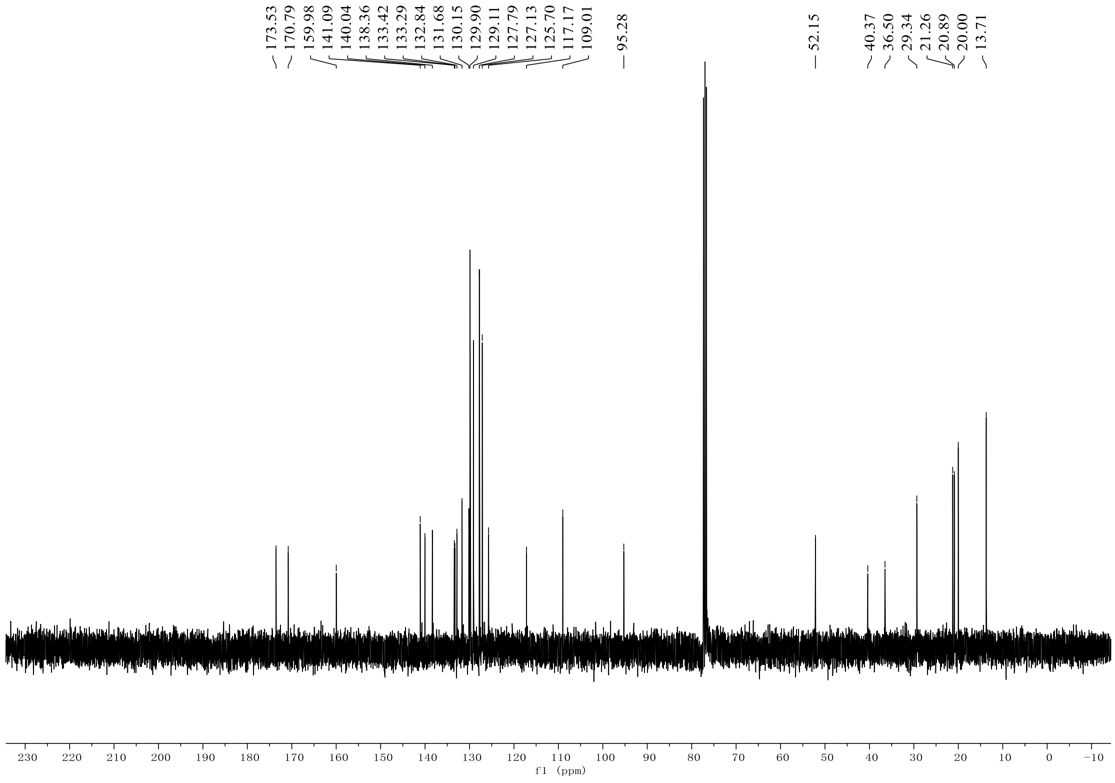
173.53
170.79
159.98
141.09
140.04
138.36
133.42
133.29
132.84
131.68
130.15
129.90
129.11
127.79
127.13
125.70
117.17
109.01
95.28
52.15
40.37
36.50
29.34
21.26
20.89
20.00
13.71
f1 (ppm)

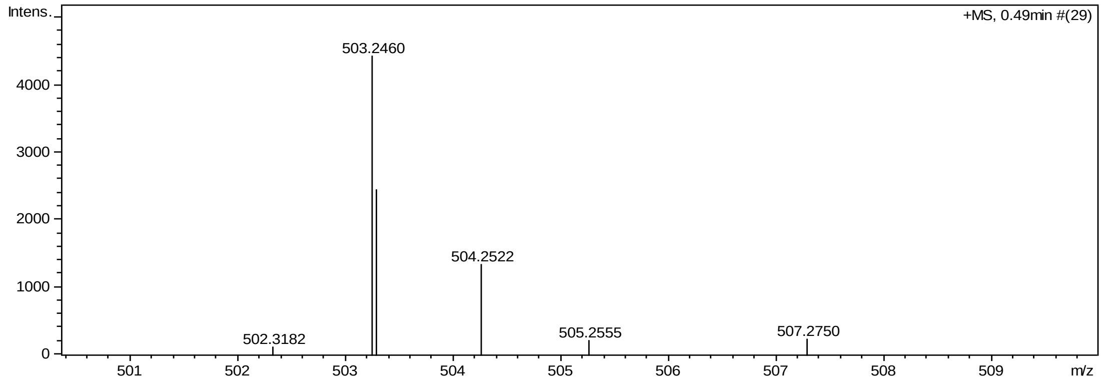
Intens.
+MS, 0.49min #(29)
503.2460
504.2522
502.3182
505.2555
507.2750
m/z

1-Benzyl-1'-(4-methylbenzoyl)-2-oxo-3'-(p-tolyl)-1',4'-dihydrospiro[indoline-3,5'-[1,2]diazepine]-6'-carbonitrile (3h): yellow solid, 0.386 g, 72%, m.p. 245-246 ºC; ^{1}H NMR (400 MHz, $CDCl_3$) δ: 8.61 (s, 1H, ArH), 7.66 (d, *J* = 8.0 Hz, 2H, ArH), 7.34-7.28 (m, 5H, ArH), 7.25-7.18 (m, 6H, ArH), 7.13 (d, *J* = 7.2 Hz, 1H, ArH), 7.02 (m, *J* = 8.0 Hz, 2H, ArH), 6.97 (t, *J* = 7.6 Hz, 1H, ArH), 6.83 (d, *J* = 8.0 Hz,1H, ArH), 4.95 (s, 2H, CH_2), 3.48 (d, *J* = 13.6 Hz, 1H, CH), 3.23 (d, *J* = 14.0 Hz, 1H, CH), 2.43 (s, 3H, CH_3), 2.31 (s, 3H, CH_3) ppm; ^{13}C NMR (100 MHz, $CDCl_3$) δ: 173.9, 170.6, 159.3, 142.6, 142.1, 141.1, 138.8, 135.1, 133.4, 130.4, 130.1, 129.9, 129.2, 129.2, 128.9, 128.5, 127.9, 127.4, 127.4, 127.2, 124.8, 123.5, 117.4, 110.0, 94.3, 52.3, 44.4, 36.8, 21.7, 21.3 ppm; IR (KBr) υ : 2921, 2868, 2209, 1748, 1715, 1698, 1682, 1568, 1516, 1507, 1489, 1471, 1287, 1029, 900, 857, 819 cm^{-1}; MS (*m/z*): HRMS (ESI-TOF) Calcd. for $C_{35}H_{29}N_4O_2$ ($[M+H]^+$): 537.2307, Found: 537.2285.

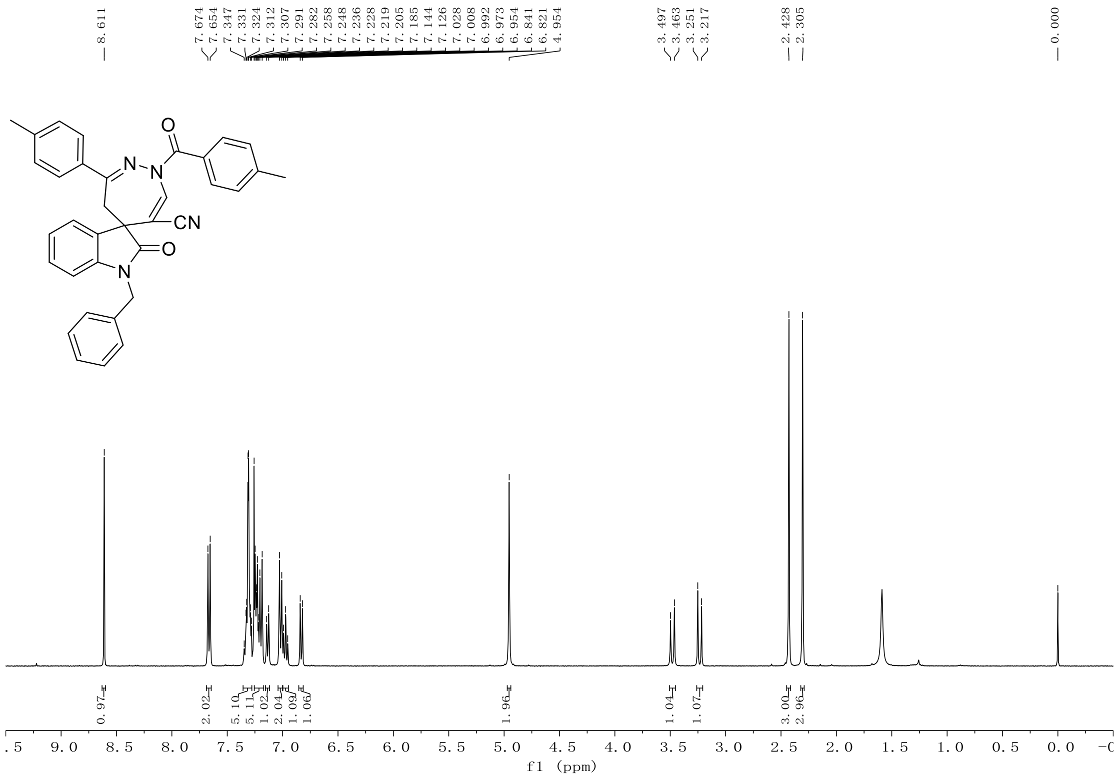

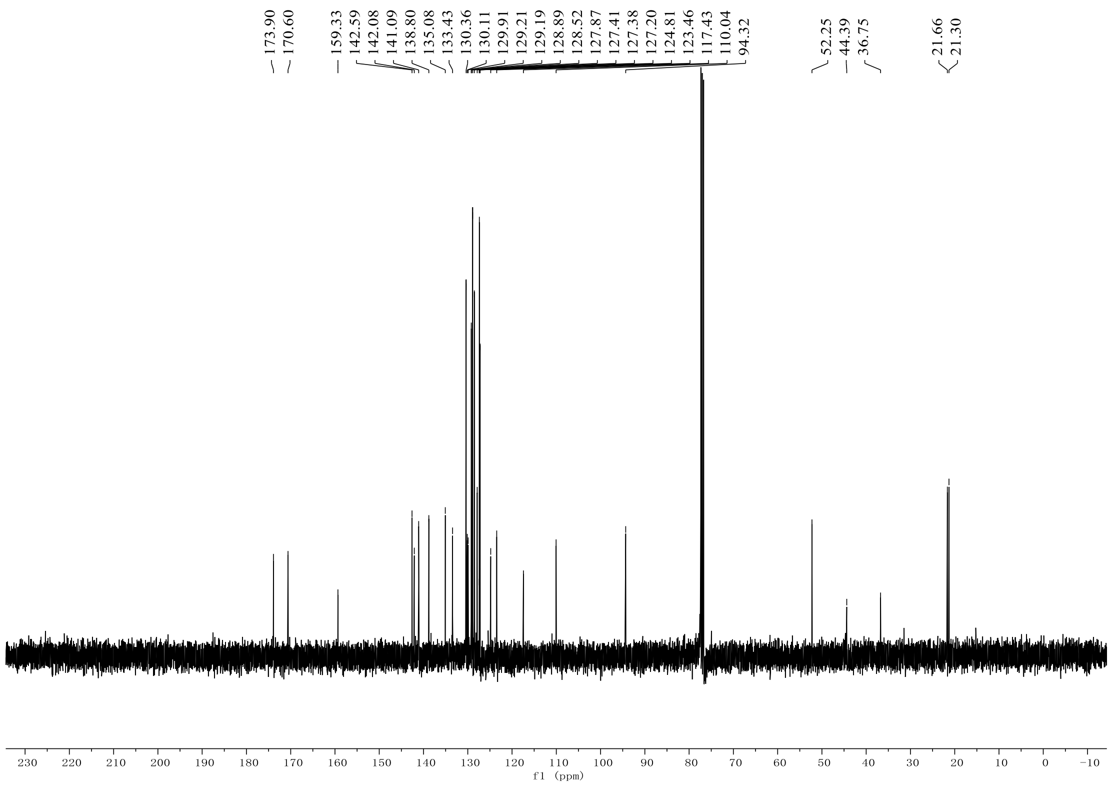

173.90
170.60
159.33
142.59
142.08
141.09
138.80
135.08
133.43
130.36
130.11
129.91
129.21
129.19
128.89
128.52
127.87
127.41
127.38
127.20
124.81
123.46
117.43
110.04
94.32
52.25
44.39
36.75
21.66
21.30
230 220 210 200 190 180 170 160 150 140 130 120 110 100 90 80 70 60 50 40 30 20 10 0 -10
f1 (ppm)

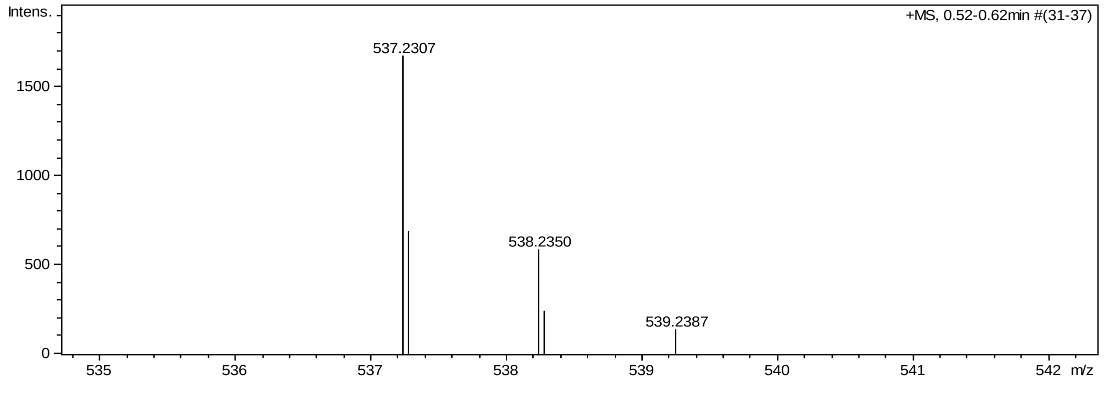

Intens.
+MS, 0.52-0.62min #(31-37)
537.2307
538.2350
539.2387
1500
1000
500
0
535 536 537 538 539 540 541 542 m/z

1-Butyl-5-methyl-1'-(4-methylbenzoyl)-2-oxo-3'-(p-tolyl)-1',4'-dihydrospiro[indoline-3,5'-[1,2]diazepine]-6'-carbonitrile (3i): yellow solid, 0.382 g, 74%, m.p. 147-148 ºC; ^{1}H NMR (600 MHz, $CDCl_3$) δ: 8.56 (s, 1H, ArH), 7.65 (d, *J* = 7.8 Hz, 2H, ArH), 7.26-7.20 (m, 4H, ArH), 7.12 (d, *J* = 7.8 Hz, 1H, ArH), 7.03 (d, *J* = 7.8 Hz, 2H, ArH), 6.93 (s, 1H, ArH), 6.83 (d, *J* = 7.8 Hz, 1H, ArH), 3.80-3.76 (m, 1H, CH), 3.70-3.66 (m, 1H, CH), 3.43 (d, *J* = 13.2 Hz, 1H, CH), 3.16 (d, *J* = 13.2 Hz, 1H, CH), 2.43 (s, 3H, CH_3), 2.30 (s, 3H, CH_3), 2.18 (s, 3H, CH_3), 1.70-1.66 (m, 2H, CH_2), 1.39-1.36 (m, 2H, CH_2), 0.94 (t, *J* = 7.2 Hz, 3H, CH_3) ppm; ^{13}C NMR (100 MHz, $CDCl_3$) δ: 173.6, 170.6, 159.9, 142.5, 141.0, 140.0, 138.6, 133.5, 132.8, 130.3, 130.2, 130.1, 129.3, 129.1, 128.5, 127.2, 125.7, 117.3, 109.0, 94.9, 52.1, 40.4, 36.5, 29.3, 21.7, 21.3, 20.9, 20.0, 13.7 ppm; IR (KBr) υ : 2931, 2875, 2212, 1711, 1627, 1603, 1497, 1442, 1349, 1278, 1189, 1110, 1063, 1028, 956, 907, 862, 818 cm^{-1}; MS (*m*/*z*): HRMS (ESI-TOF) Calcd. for $C_{33}H_{32}N_4O_2Na$ ($[M+Na]^+$): 539.2417, Found: 539.2430.

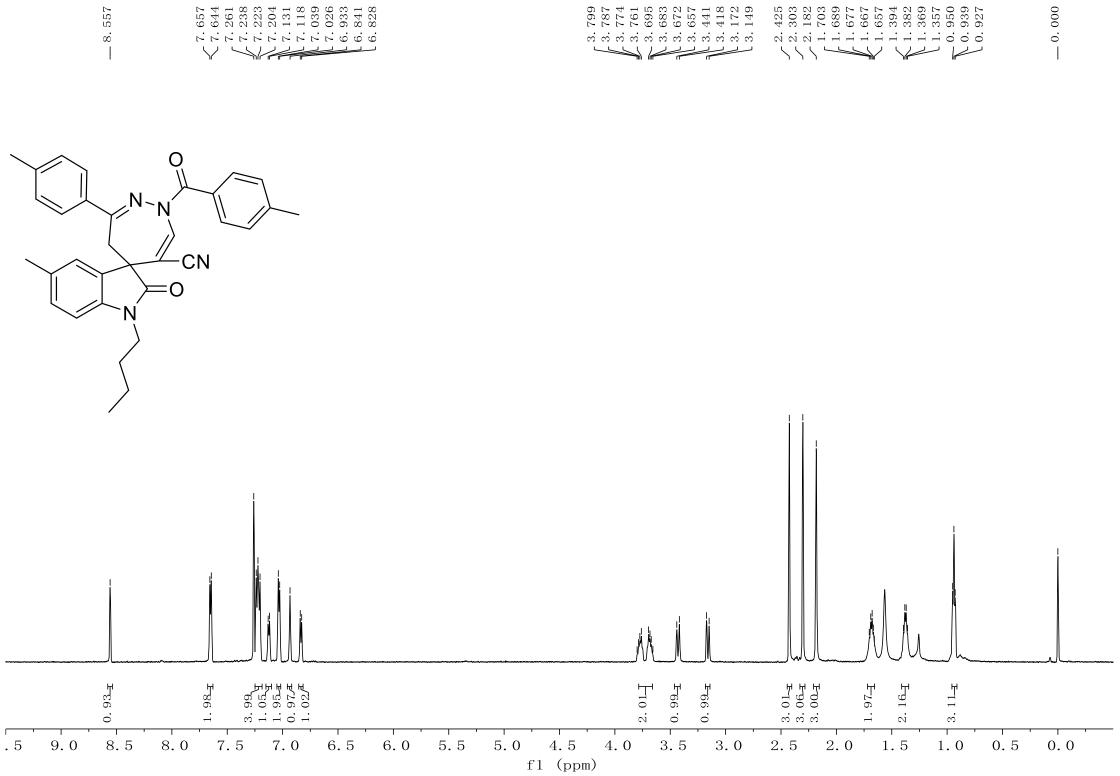

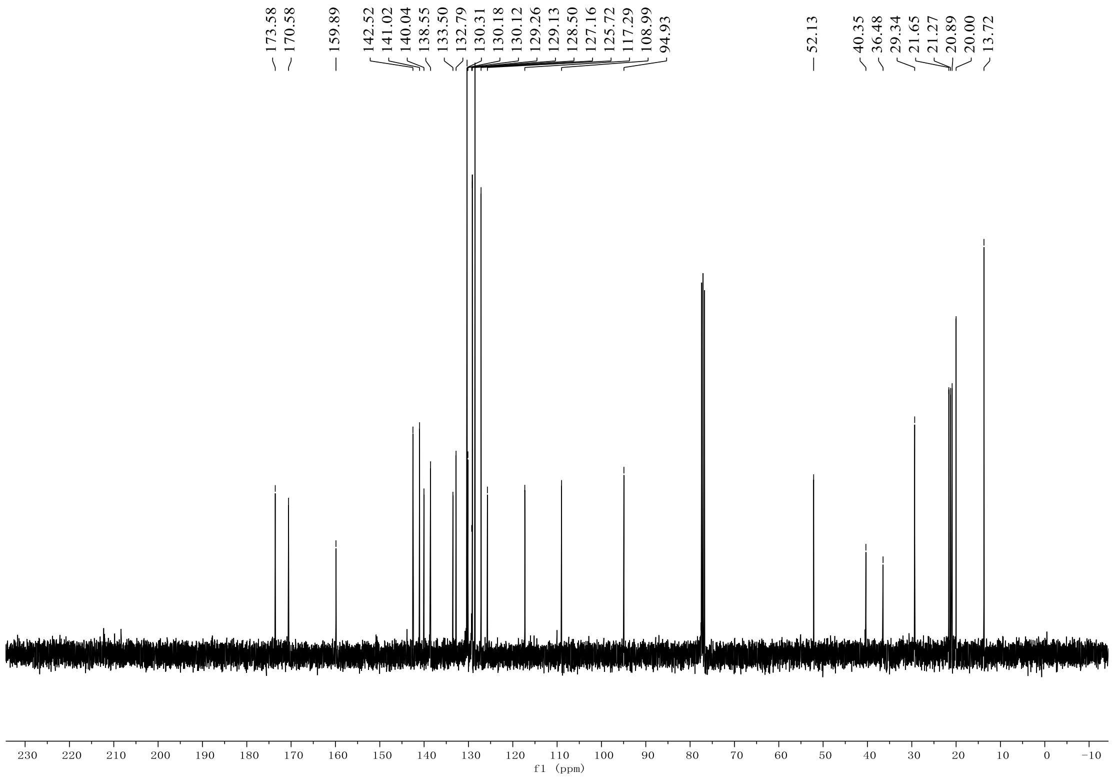
173.58
170.58
159.89
142.52
141.02
140.04
138.55
133.50
132.79
130.31
130.18
130.12
129.26
129.13
128.50
127.16
125.72
117.29
108.99
94.93
52.13
40.35
36.48
29.34
21.65
21.27
20.89
20.00
13.72
230 220 210 200 190 180 170 160 150 140 130 120 110 100 90 80 70 60 50 40 30 20 10 0 -10
f1 (ppm)

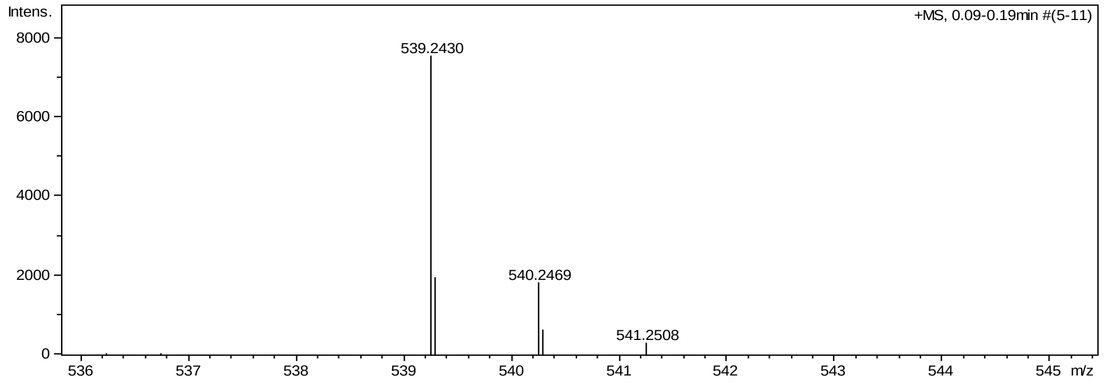
Intens.
+MS, 0.09-0.19min #(5-11)
539.2430
540.2469
541.2508
8000
6000
4000
2000
0
536 537 538 539 540 541 542 543 544 545 m/z

1-Benzyl-5-methyl-1'-(4-methylbenzoyl)-2-oxo-3'-(p-tolyl)-1',4'-dihydrospiro[indoline-3,5'-[1,2]diazepine]-6'-carbonitrile (3j): yellow solid, 0.374 g, 68%, m.p. 232-233 ºC; ^{1}H NMR (400 MHz, $CDCl_3$) δ: 8.60 (s, 1H, ArH), 7.66 (d, *J* = 8.0 Hz, 2H, ArH), 7.34-7.27 (m, 5H, ArH), 7.25 (s, 1H, ArH), 7.21 (d, *J* = 8.8 Hz, 3H, ArH), 7.02 (d, *J* = 8.0 Hz, 3H, ArH), 6.96(s, 1H, ArH), 6.70 (d, *J* = 8.0 Hz, 1H, ArH), 4.93 (s, 2H, CH_2), 3.46 (d, *J* = 13.6 Hz, 1H, CH), 3.22 (d, *J* = 13.6 Hz, 1H, CH), 2.43 (s, 3H, CH_3), 2.31 (s, 3H, CH_3), 2.16 (s, 3H, CH_3) ppm; ^{13}C NMR (100 MHz, $CDCl_3$) δ: 173.8, 170.6, 159.6, 142.6, 141.0, 139.6, 138.7, 135.2, 133.5, 133.2, 130.4, 130.1, 129.2, 129.0, 128.8, 128.5, 127.8, 127.4, 127.2, 125.6, 117.5, 109.8, 94.6, 52.3, 44.4, 36.8, 21.7, 21.3, 20.9 ppm; IR (KBr) υ: 2957, 2931, 2868, 2213, 1721, 1628,1495, 1443, 1368, 1270, 1194, 1110, 1087, 1007, 907, 867, 821 cm^{-1}; MS (*m/z*): HRMS (ESI-TOF) Calcd. for $C_{36}H_{31}N_4O_2$ ([M+H]$^{+}$): 551.2442, Found: 551.2434.

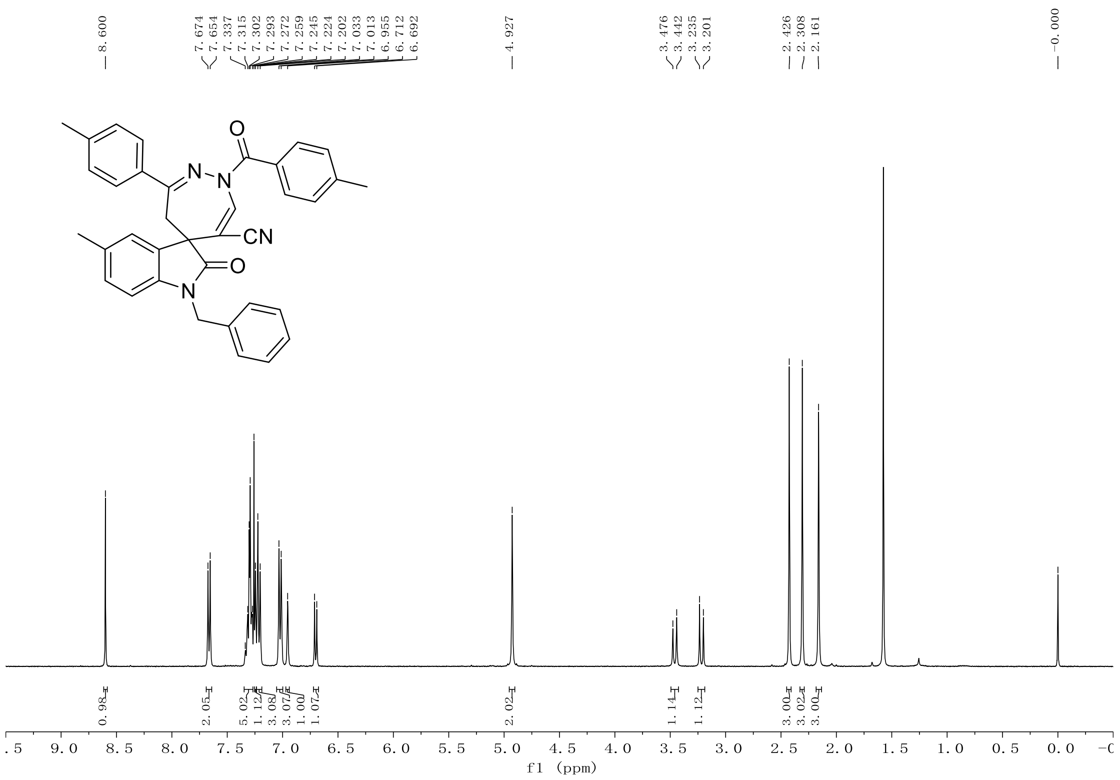

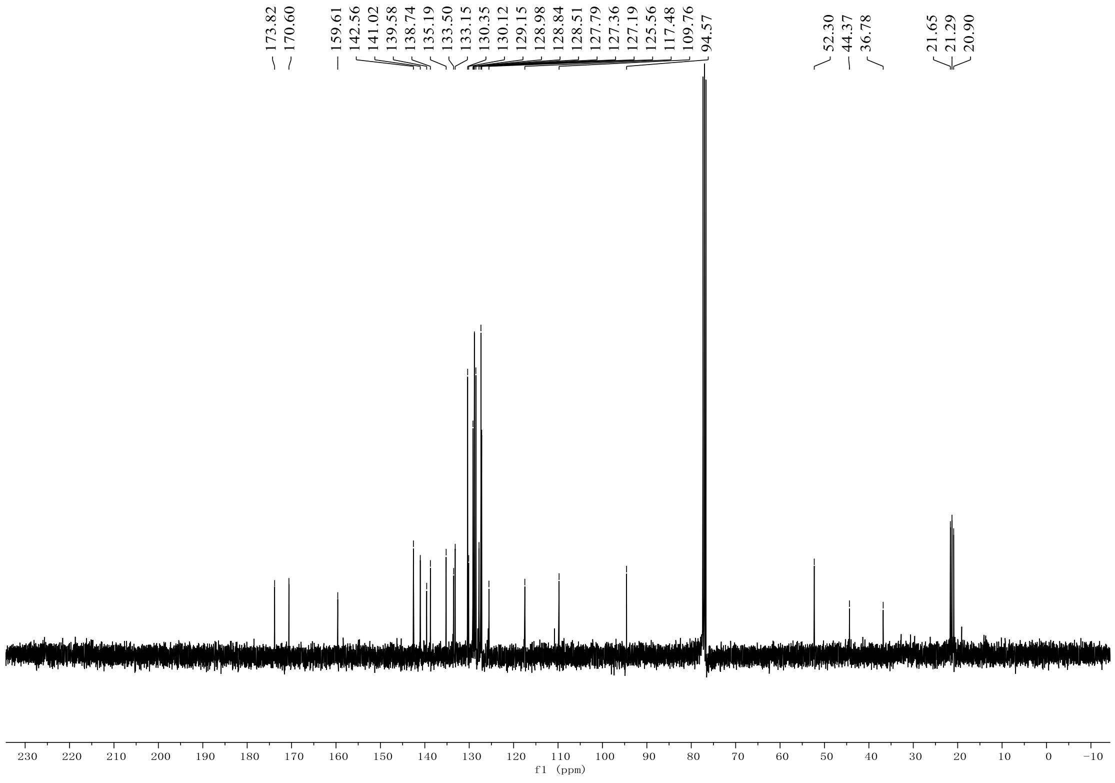
173.82
170.60
159.61
142.56
141.02
139.58
138.74
135.19
133.50
133.15
130.35
130.12
129.15
128.98
128.84
128.51
127.79
127.36
127.19
125.56
117.48
109.76
94.57
52.30
44.37
36.78
21.65
21.29
20.90
f1 (ppm)

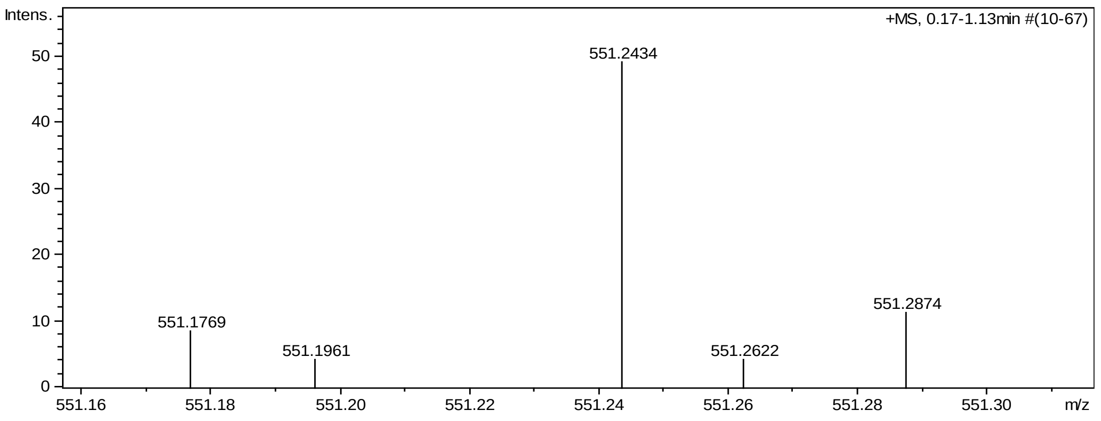
Intens.
+MS, 0.17-1.13min #(10-67)
551.2434
551.1769
551.1961
551.2622
551.2874
m/z

1'-Benzoyl-1-butyl-3'-(4-methoxyphenyl)-5-methyl-2-oxo-1',4'-dihydrospiro[indoline-3,5'-[1,2]diazepine]-6'-carbonitrile (3k): yellow solid, 0.388 g, 75%, m.p. 176-177 °C; ^{1}H NMR (600 MHz, $CDCl_3$) δ: 8.55 (s, 1H, ArH), 7.70 (d, *J* = 7.2 Hz, 2H, ArH), 7.54 (t, *J* = 7.2 Hz,1H, ArH), 7.46-7.42 (m, 2H, ArH), 7.21 (d, *J* = 8.4 Hz, 2H, ArH), 7.13 (d, *J* = 7.8 Hz, 1H, ArH), 6.92 (s, 1H, ArH), 6.84 (d, *J* = 7.8 Hz, 1H, ArH), 6.71 (d, *J* = 8.4 Hz, 2H, ArH), 3.81-3.66 (m, 2H, CH_2), 3.77 (s, 3H, OCH_3), 3.44 (d, *J* = 13.8 Hz, 1H, CH), 3.14 (d, *J* = 13.8 Hz, 1H, CH), 2.17 (s, 3H, CH_3), 1.72-1.67 (m, 2H, CH_2), 1.41-1.38 (m, 2H, CH_2), 0.95 (t, *J* = 7.2 Hz, 3H, CH_3) ppm; 13C NMR (151 MHz, CDCl3) δ: 173.6, 170.8, 161.7, 159.9, 140.1, 138.6, 133.5, 132.9, 131.7, 130.2, 129.9, 129.2, 128.9, 128.7, 127.8, 125.8, 117.2, 113.8, 109.0, 95.3, 55.4, 52.2, 40.5, 36.4, 29.4, 21.0, 20.1, 13.8.cm^{-1}; MS (*m/z*): HRMS (ESI-TOF) Calcd. for $C_{32}H_{31}N_4O_3$ ([M+H]$^+$): 519.2408, Found: 519.2391.

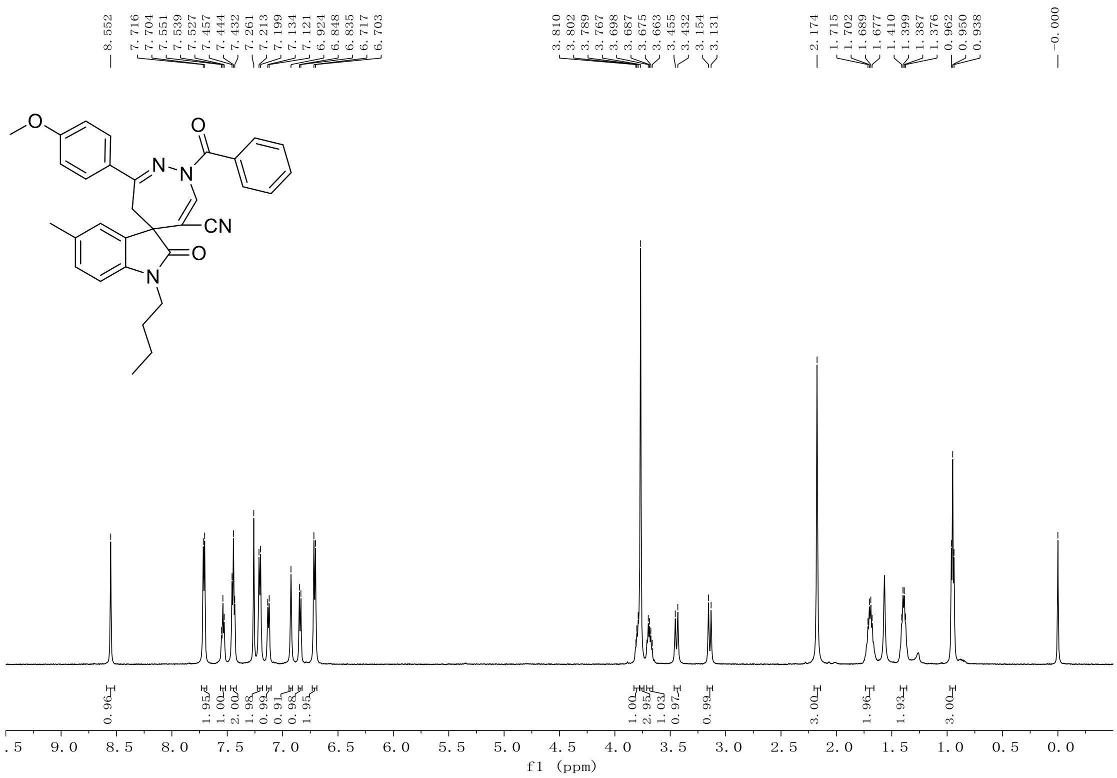

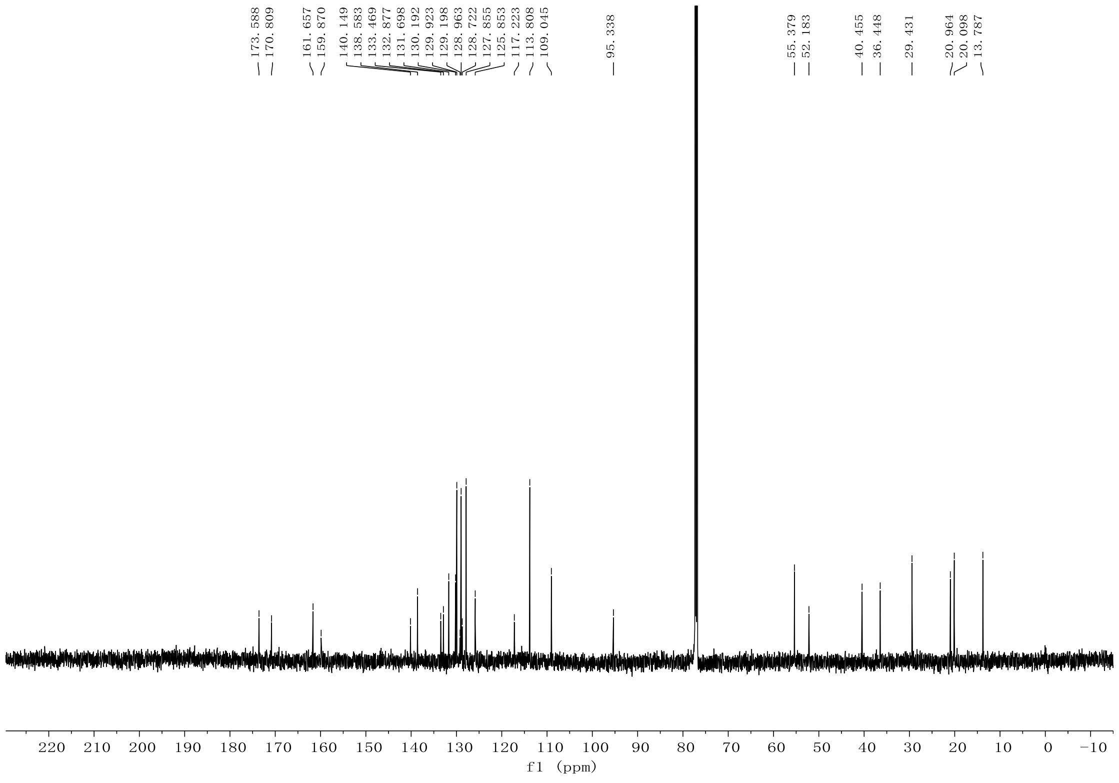
173.588
170.809
161.657
159.870
140.149
138.583
133.469
132.877
131.698
130.192
129.923
129.198
128.963
128.722
127.855
125.853
117.223
113.808
109.045
95.338
55.379
52.183
40.455
36.448
29.431
20.964
20.098
13.787
220 210 200 190 180 170 160 150 140 130 120 110 100 90 80 70 60 50 40 30 20 10 0 −10
f1 (ppm)

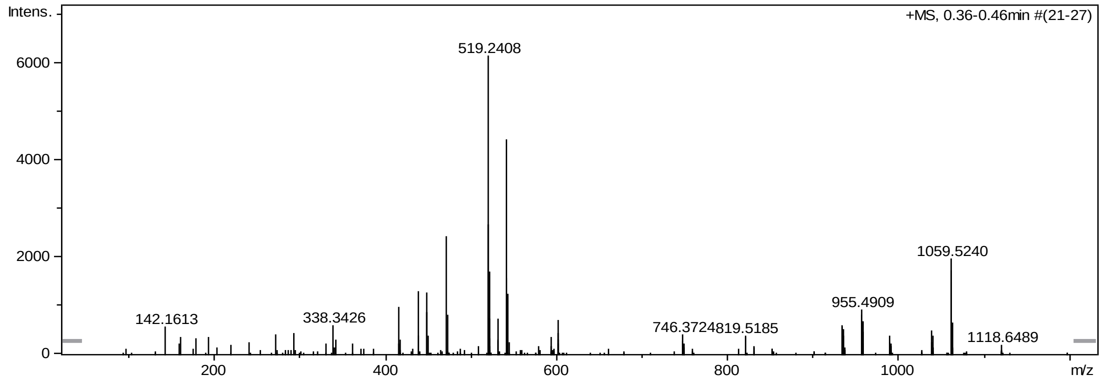
Intens.
+MS, 0.36-0.46min #(21-27)
519.2408
1059.5240
955.4909
142.1613
338.3426
746.3724
819.5185
1118.6489
6000
4000
2000
0
200
400
600
800
1000
m/z

1'-Benzoyl-1-benzyl-3'-(4-methoxyphenyl)-5-methyl-2-oxo-1',4'-dihydrospiro[indoline-3,5'-[1,2]diazepine]-6'-carbonitrile (3l): yellow solid, 0.381 g, 72%, m.p. 200-201 °C; ^{1}H NMR (400 MHz, $CDCl_3$) δ: 8.59 (s, 1H, ArH), 7.73-7.71 (m, 2H, ArH), 7.56-7.52 (m, 1H, ArH), 7.46-7.42 (m, 2H, ArH), 7.35-7.27 (m, 5H, ArH), 7.21-7.19 (m, 2H, ArH), 7.04-7.02 (m, 1H, ArH), 6.95 (s, 1H, ArH), 6.72-6.68 (m, 3H, ArH), 4.98-4.89 (m, 2H, CH_2), 3.76 (s, 3H, OCH_3), 3.46 (d, J = 14.0 Hz, 1H, CH), 3.20 (d, J = 13.6 Hz, 1H, CH), 2.16 (s, 3H, CH_3) ppm; ^{13}C NMR (100 MHz, $CDCl_3$) δ: 173.8, 170.8, 161.6, 159.6, 139.6, 138.7, 135.2, 133.3, 133.2, 131.7, 130.2, 129.9, 128.9, 128.9, 128.6, 127.8, 127.8, 127.4, 125.6, 117.4, 113.8, 109.8, 94.9, 55.3, 52.3, 44.4, 36.7, 20.9 ppm; IR (KBr) υ: 2923, 2852, 1752, 1686, 1561, 1512, 1496, 1438, 1383, 1345, 1283, 1257, 1179, 1115, 985, 892, 757 cm^{-1}; MS (m/z): HRMS (ESI-TOF) Calcd. for $C_{35}H_{29}N_4O_3H$ ([M+Na]$^+$): 553.2239, Found: 553.2234.

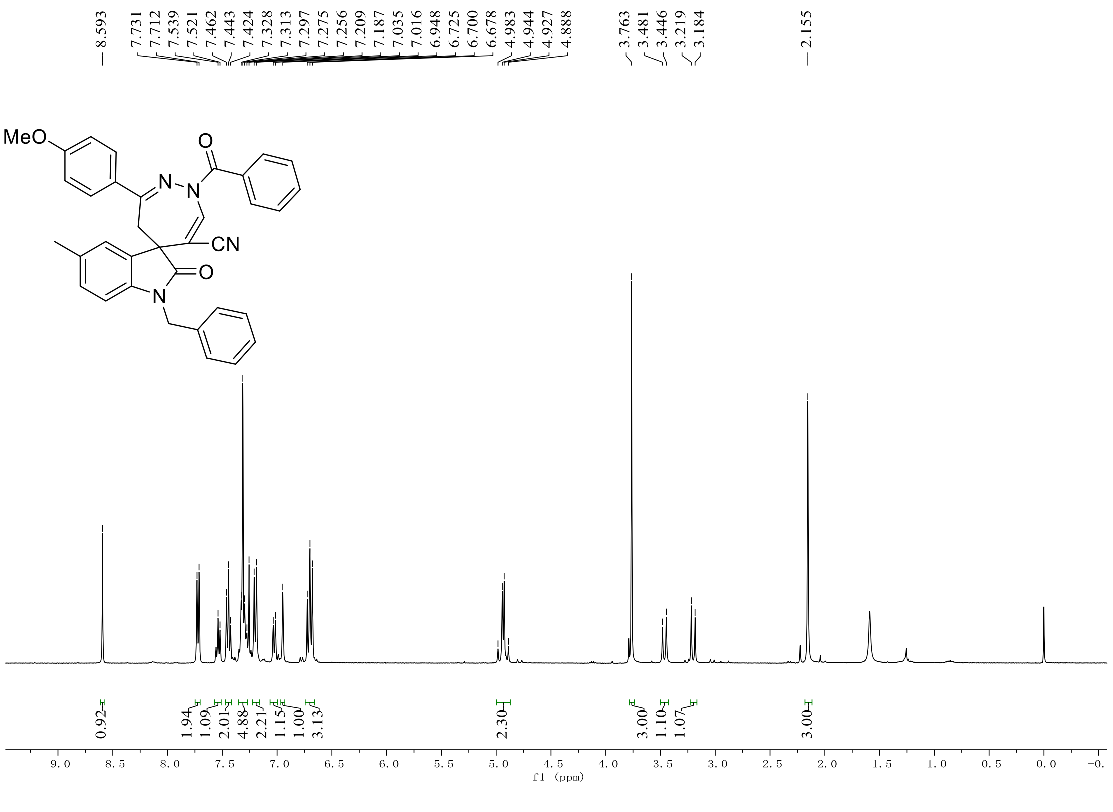

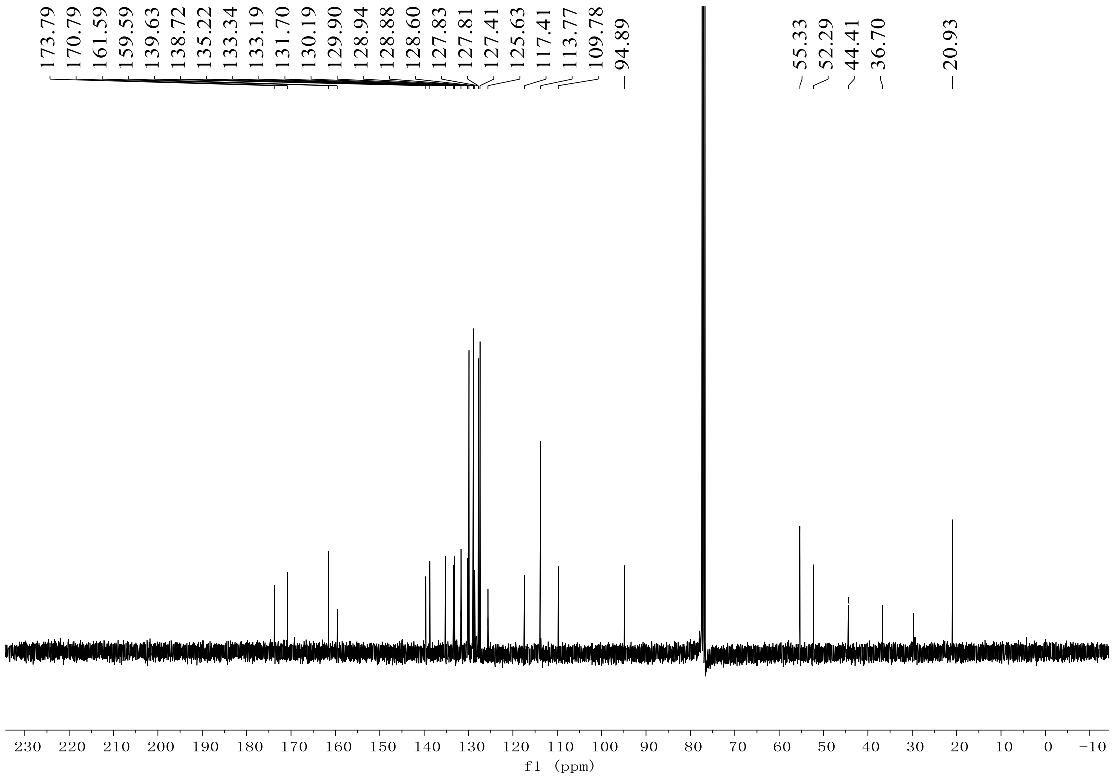
173.79
170.79
161.59
159.59
139.63
138.72
135.22
133.34
133.19
131.70
130.19
129.90
128.94
128.88
128.60
127.83
127.81
127.41
125.63
117.41
113.77
109.78
94.89
55.33
52.29
44.41
36.70
20.93
230 220 210 200 190 180 170 160 150 140 130 120 110 100 90 80 70 60 50 40 30 20 10 0 -10
f1 (ppm)

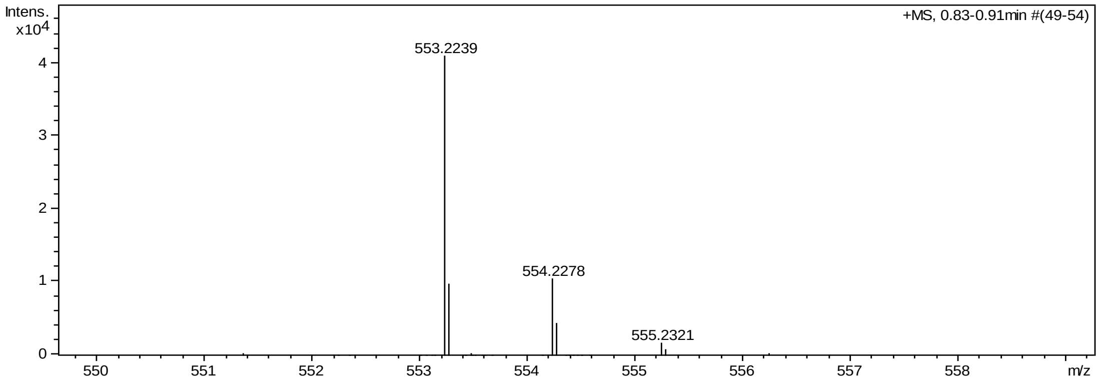
Intens.
x10^4
+MS, 0.83-0.91min #(49-54)
553.2239
554.2278
555.2321
550 551 552 553 554 555 556 557 558 m/z

1'-Benzoyl-1-benzyl-5-chloro-3'-(3-chlorophenyl)-2-oxo-1',4'-dihydrospiro[indoline-3,5'-[1,2]diazepine]-6'-carbonitrile (3m): white solid, 0.355 g, 62%, m.p. 208-211 °C; ^{1}H NMR (600 MHz, $CDCl_3$) δ: 8.62 (s, 1H, ArH), 7.74-7.71 (m, 2H, ArH), 7.59-7.56 (m, 1H, ArH), 7.49-7.46 (m, 2H, ArH), 7.34-7.33 (m, 4H, ArH), 7.32-7.29 (m, 1H, ArH), 7.27-7.26 (m, 1H, ArH), 7.25-7.24 (m, 2H, ArH), 7.17-7.16 (m, 3H, ArH), 6.77-6.76 (m, 1H, ArH), 4.96-4.90 (m, 2H, CH_2), 3.39 (d, J = 14.4 Hz, 1H, CH), 3.22 (d, J = 13.8 Hz, 1H, CH) ppm; ^{13}C NMR (151 MHz, $CDCl_3$) δ: 173.4, 170.8, 157.1, 140.6, 138.5, 138.1, 134.8, 134.5, 132.8, 132.1, 130.8, 130.2, 129.9, 129.8, 129.3, 129.1, 128.2, 128.0, 127.3, 127.3, 125.3, 125.1, 117.0, 111.3, 93.9, 52.4, 44.7, 36.9 ppm; IR (KBr) υ:3085, 2190, 1825, 1755, 1678, 1568, 1482, 1319, 1280, 1173, 966, 905, 842, 805 cm^{-1}; MS (m/z): HRMS (ESI-TOF) Calcd. for $C_{33}H_{22}Cl_2N_4O_2Na$ ($[M+Na]^+$): 599.1012, Found: 599.1054.

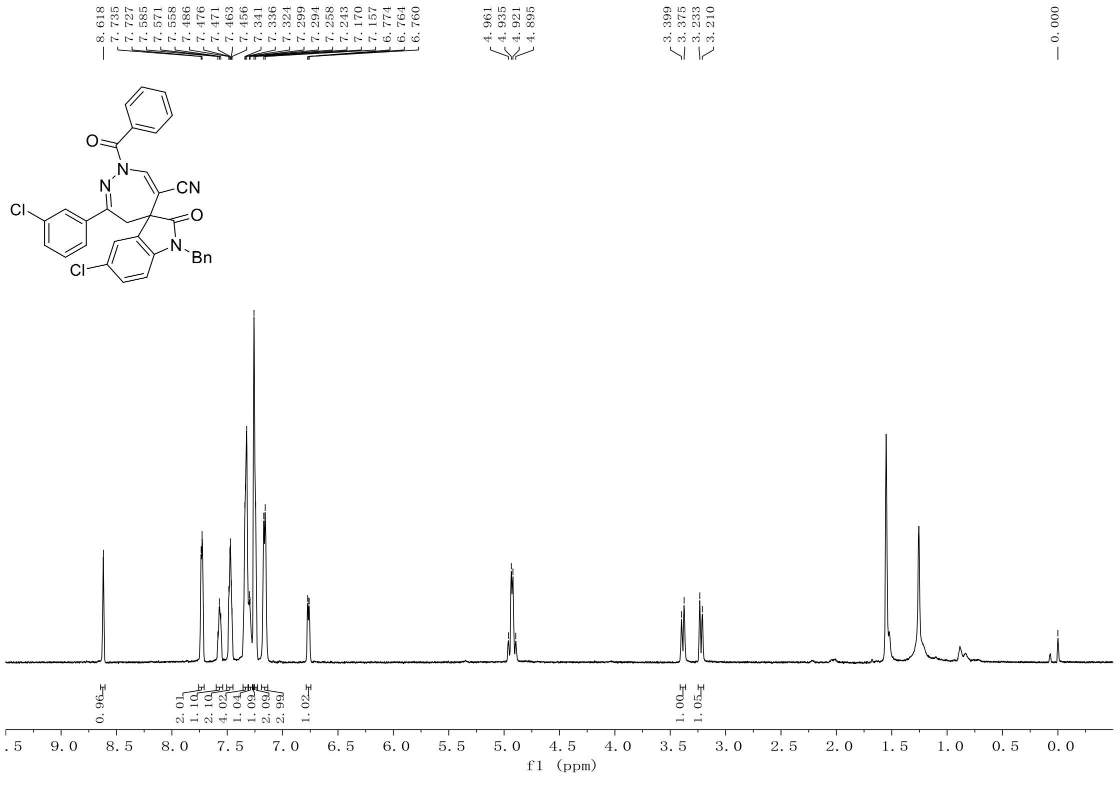

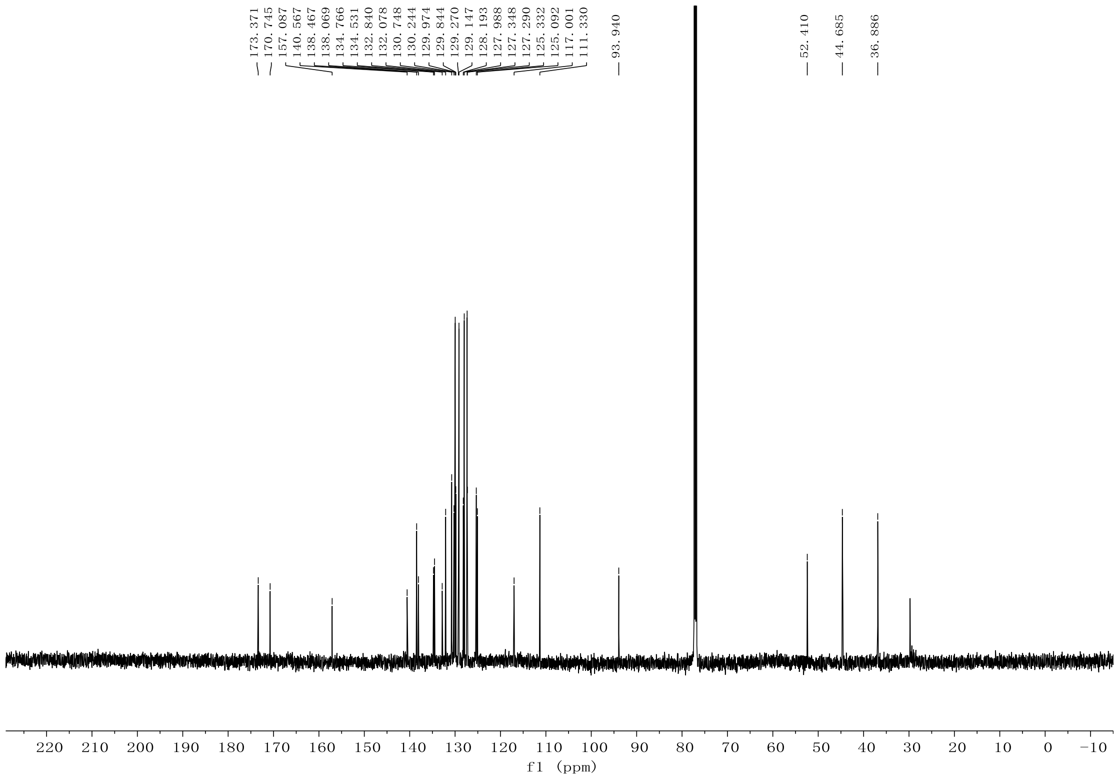
173.371
170.745
157.087
140.567
138.467
138.069
134.766
134.531
132.840
132.078
130.748
130.244
129.974
129.844
129.270
129.147
128.193
127.988
127.348
127.290
125.332
125.092
117.001
111.330
93.940
52.410
44.685
36.886
220 210 200 190 180 170 160 150 140 130 120 110 100 90 80 70 60 50 40 30 20 10 0 −10
f1 (ppm)

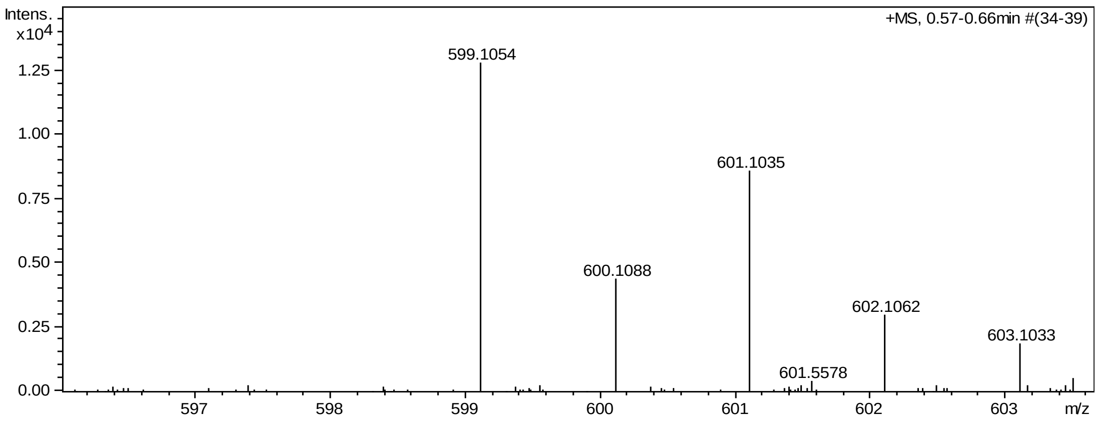
Intens.
x10^4
+MS, 0.57-0.66min #(34-39)
599.1054
601.1035
600.1088
602.1062
603.1033
601.5578
1.25
1.00
0.75
0.50
0.25
0.00
597
598
599
600
601
602
603
m/z

Methyl 1'-benzoyl-1-benzyl-5-chloro-2-oxo-3'-phenyl-1',4'-dihydrospiro[indoline-3,5'-[1,2]diazepine]-6'-carboxylate (5a): yellow solid, 0.391 g, 68%, m.p. 189-191 ºC; ^{1}H NMR (400 MHz, $CDCl_3$) δ: 9.19 (s, 1H, ArH), 7.73-7.71 (m, 2H, ArH), 7.56-7.53 (m, 1H, ArH), 7.48-7.42 (m, 4H, ArH), 7.38-7.35 (m, 2H, ArH), 7.32-7.31 (m, 1H, ArH), 7.29-7.27 (m, 1H, ArH), 7.14 (t, J = 8.0 Hz, 2H, ArH), 7.09-7.05 (m, 3H, ArH), 6.86-6.85 (m, 1H, ArH), 6.70 (d, J = 8.4 Hz, 1H, ArH), 5.02 (s, 2H, CH_2), 3.65 (s, 3H, OCH_3), 3.49 (d, J = 13.6 Hz, 1H, CH), 3.10 (d, J = 13.6 Hz, 1H, CH) ppm; ^{13}C NMR (100 MHz, $CDCl_3$) δ: 176.3, 171.3, 165.8, 160.6, 141.1, 137.3, 135.9, 135.5, 133.8, 131.6, 130.6, 129.7, 128.9, 128.5, 128.4, 127.9, 127.8, 127.5, 127.0, 124.4, 111.6, 110.4, 52.2, 51.1, 44.4, 36.8 ppm ; IR (KBr) υ : 2924, 2853, 1721, 1608, 1484, 1456, 1430, 1340, 1170, 812 cm^{-1}; MS (m/z): HRMS (ESI-TOF) Calcd. for $C_{34}H_{27}ClN_3O_4$ ($[M+H]^+$): 576.1685, Found: 576.1683.

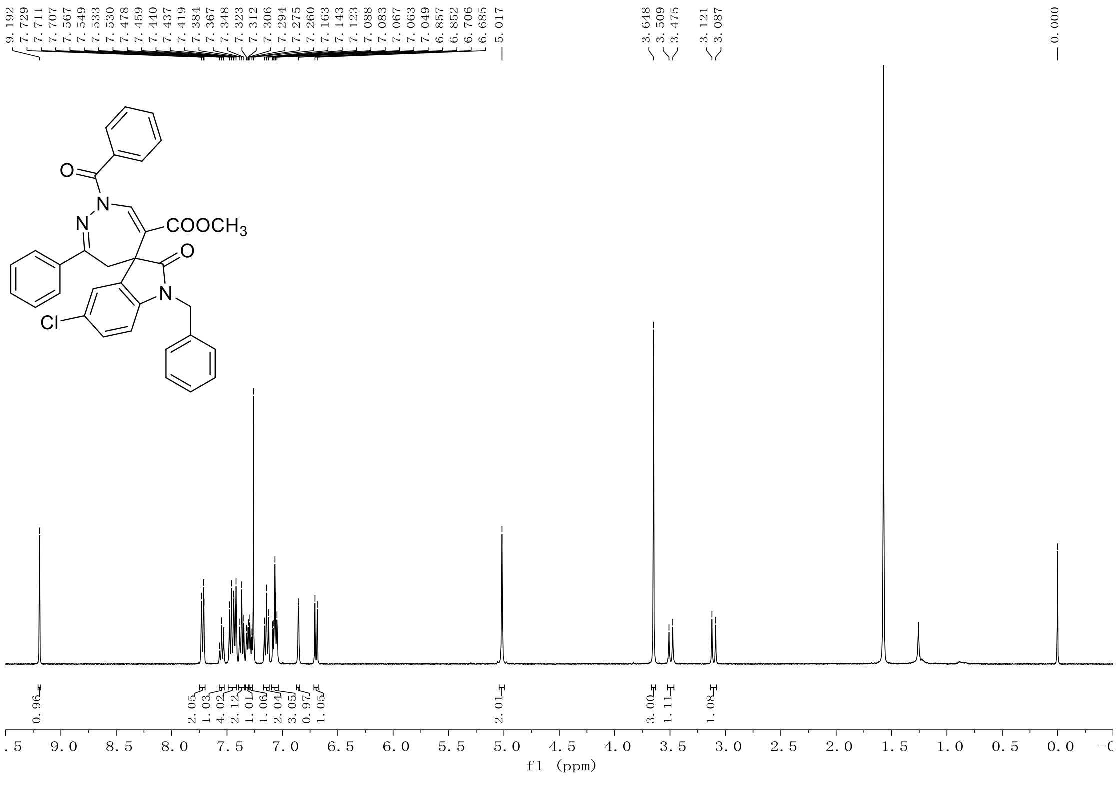

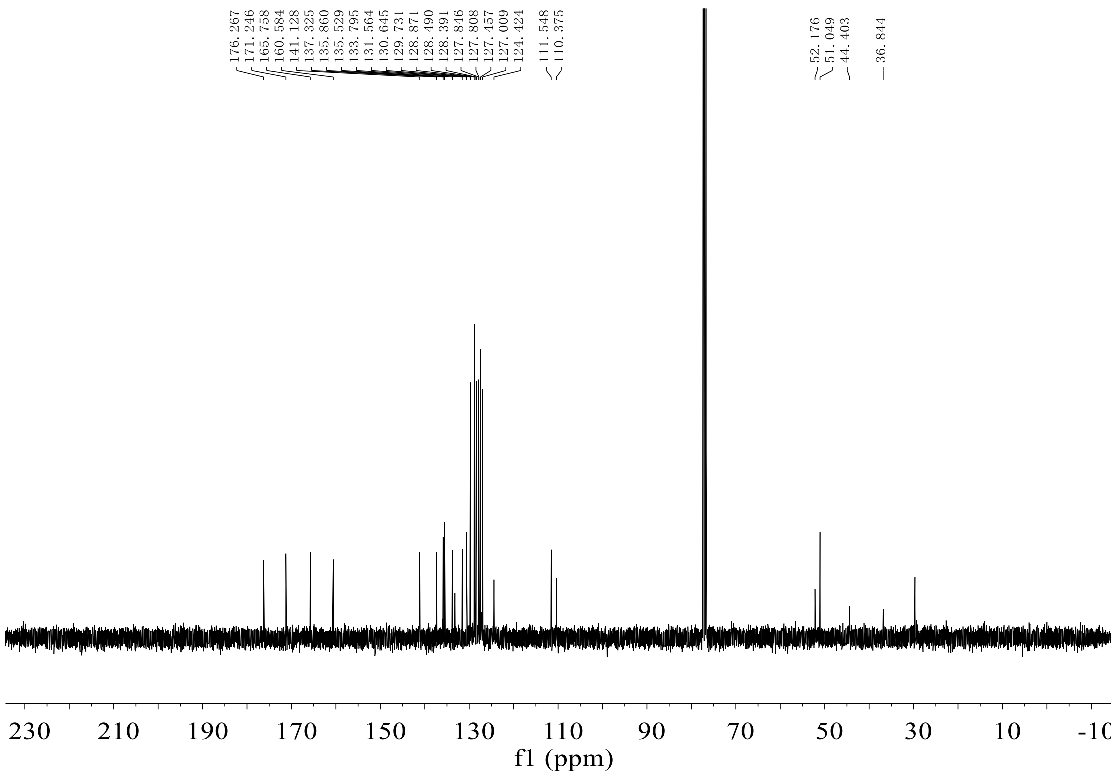
176.267
171.246
165.758
160.584
141.128
137.325
135.860
135.529
133.795
131.564
130.645
129.731
128.871
128.490
128.391
127.846
127.808
127.457
127.009
124.424
111.548
110.375
52.176
51.049
44.403
36.844
230 210 190 170 150 130 110 90 70 50 30 10 -10
f1 (ppm)

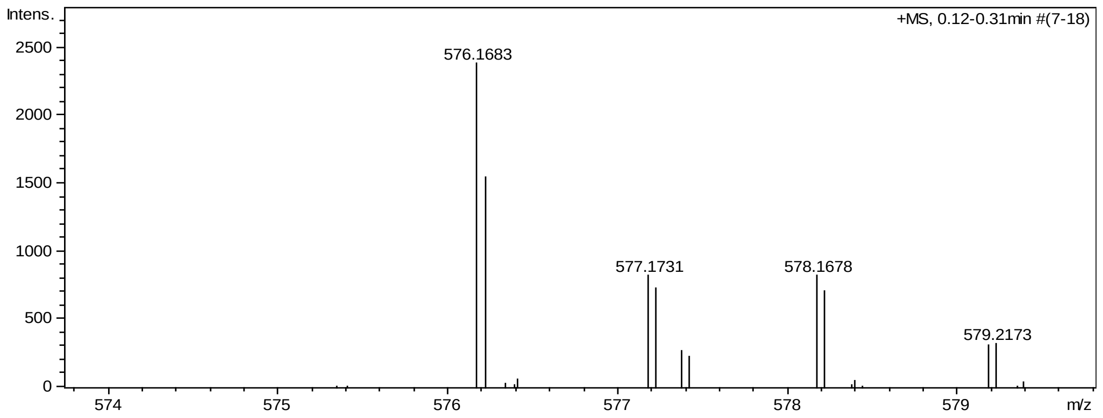
Intens.
+MS, 0.12-0.31min #(7-18)
576.1683
577.1731
578.1678
579.2173
2500
2000
1500
1000
500
0
574 575 576 577 578 579 m/z

Methyl 1'-benzoyl-1-benzyl-5-methyl-2-oxo-3'-phenyl-1',4'-dihydrospiro[indoline-3,5'-[1,2]diazepine]-6'-carboxylate (5b): yellow solid, 0.416 g, 75%, m.p. 165-167 ºC; ^{1}H NMR (600 MHz, $CDCl_3$) δ: 9.19 (s, 1H, ArH), 7.72 (d, *J* = 7.2 Hz, 2H, ArH), 7.55-7.53 (m, 1H, ArH), 7.48-7.44 (m, 4H, ArH), 7.36-7.34 (m, 2H, ArH), 7.30-7.29 (m, 1H, ArH), 7.25-7.23 (m, 1H, ArH), 7.11-7.09 (m, 2H, ArH), 7.01-6.99 (m, 2H, ArH), 6.90-6.89 (m, 1H, ArH), 6.68-6.66 (m, 2H, ArH), 5.02 (s, 2H, CH_2), 3.62 (s, 3H, OCH_3), 3.50 (d, *J* = 12.6 Hz, 1H, CH), 3.07 (d, *J* = 13.8 Hz, 1H, CH), 1.94 (s, 3H, CH_3) ppm; 13C NMR (151 MHz, CDCl3) δ: 176.6, 171.3, 165.9, 161.2, 140.1, 137.0, 136.1, 131.9, 131.5, 130.3, 129.7, 128.8, 128.2, 127.8, 127.6, 127.5, 127.2, 124.9, 112.4, 109.2, 52.1, 51.0, 44.3, 37.3, 20.7 ppm; IR (KBr) υ : 2924, 2853, 1715, 1601, 1496, 1447, 1349, 1280, 1188, 1140, 859, 785 cm^{-1}; MS (*m/z*): HRMS (ESI-TOF) Calcd. for $C_{35}H_{30}N_3O_4$ ($[M+H]^+$): 556.2231, Found: 556.2225.

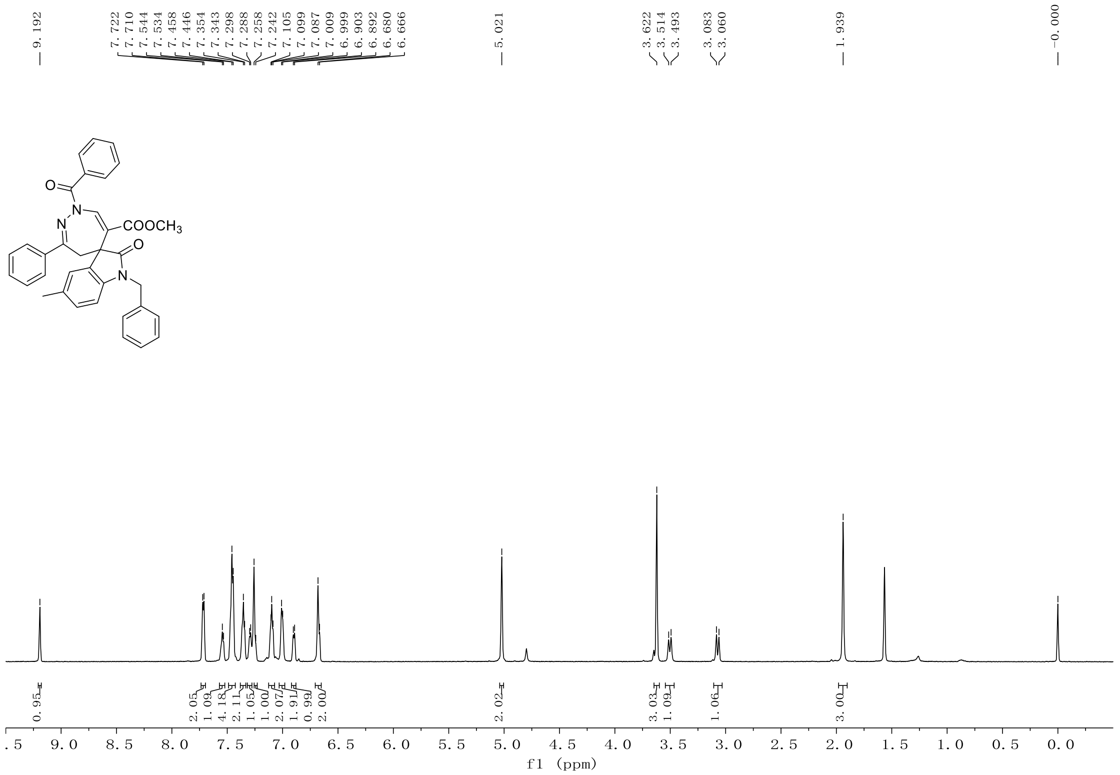

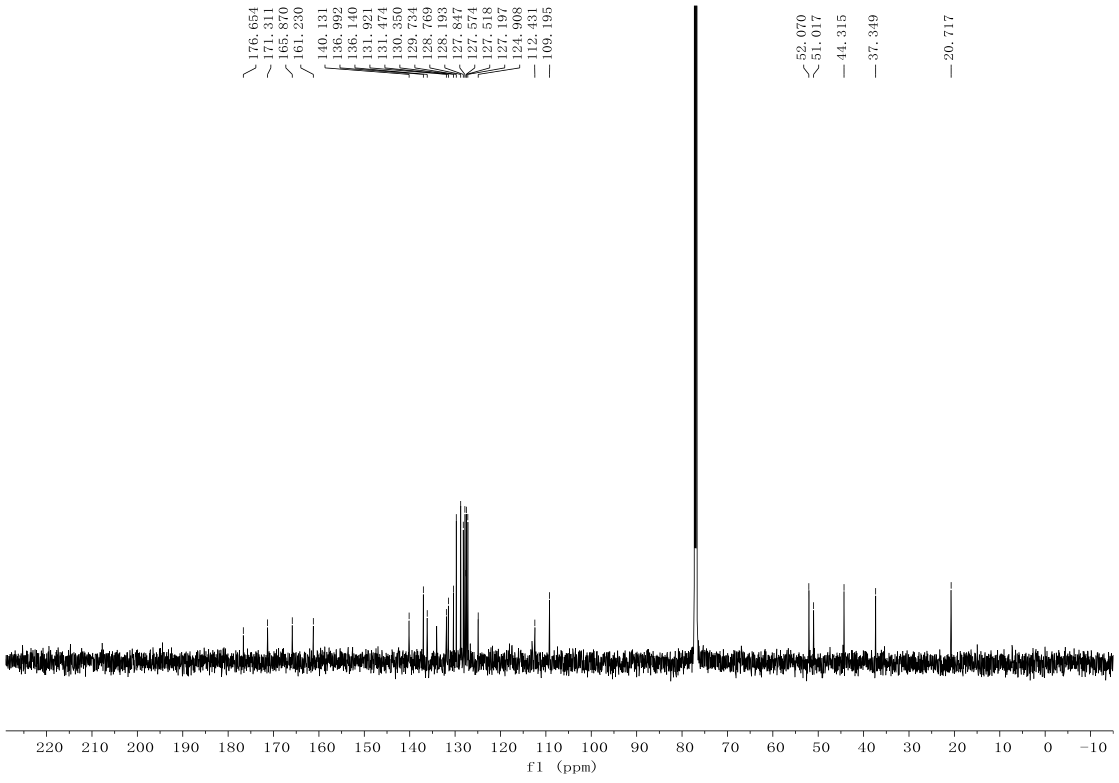

176.654
171.311
165.870
161.230
140.131
136.992
136.140
131.921
131.474
130.350
129.734
128.769
128.193
127.847
127.574
127.518
127.197
124.908
112.431
109.195
52.070
51.017
44.315
37.349
20.717
220 210 200 190 180 170 160 150 140 130 120 110 100 90 80 70 60 50 40 30 20 10 0 -10
f1 (ppm)

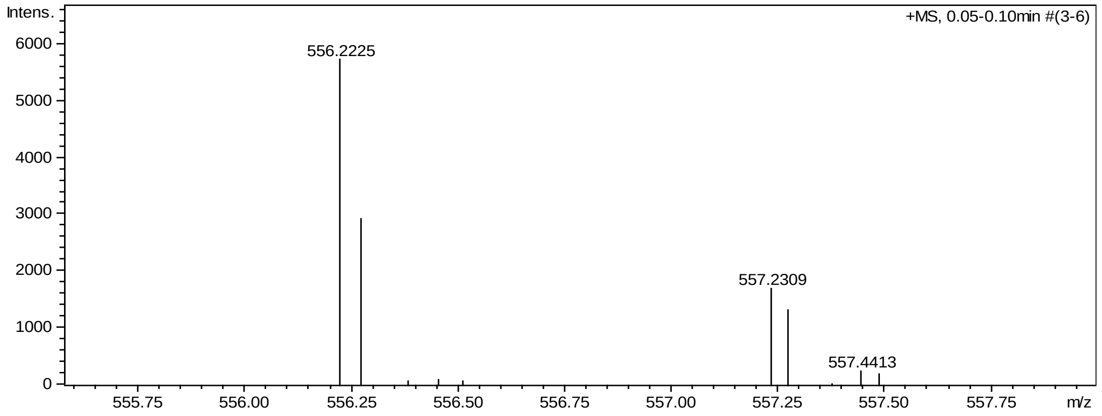

Intens.
+MS, 0.05-0.10min #(3-6)
556.2225
557.2309
557.4413
6000
5000
4000
3000
2000
1000
0
555.75
556.00
556.25
556.50
556.75
557.00
557.25
557.50
557.75
m/z

Methyl 1'-benzoyl-1-benzyl-3'-(4-chlorophenyl)-5-methyl-2-oxo-1',4'-dihydrospiro[indoline-3,5'-[1,2]diazepine]-6'-carboxylate (5c): yellow solid, 0.400 g, 68%, m.p. 213-214 °C; ^{1}H NMR (400 MHz, $CDCl_3$) δ: 9.17 (s, 1H, ArH), 7.70-7.68 (m, 2H, ArH), 7.57-7.53 (m, 1H, ArH), 7.47-7.43 (m, 4H, ArH), 7.38-7.34 (m, 2H, ArH), 7.31-7.29 (m, 1H, ArH), 7.08-7.06 (m, 2H, ArH), 6.95-6.91 (m, 3H, ArH), 6.70-6.67 (m, 2H, ArH), 5.01 (s, 2H, CH_2), 3.61 (s, 3H, OCH_3), 3.45 (d, J = 13.6 Hz, 1H, CH), 3.02 (d, J = 13.6 Hz, 1H, CH), 1.98 (s, 3H, CH_3) ppm; 13C NMR (151 MHz, CDCl3) δ: 176.5, 171.3, 165.8, 160.0, 140.1, 136.9, 136.9, 136.7, 136.1, 134.8, 134.0, 132.0, 131.6, 129.7, 129.0, 128.9, 128.8, 128.5, 128.5, 127.9, 127.7, 127.6, 124.7, 112.5, 109.3, 52.1, 51.0, 44.4, 37.3, 20.8 ppm; IR (KBr) υ: 2923, 2852, 1716, 1609, 1499, 1457, 1378, 1274, 1189, 1126, 1030, 855, 776 cm^{-1}; MS (m/z): HRMS (ESI-TOF) Calcd. for $C_{35}H_{29}ClN_3O_{34}$ ([M+H]$^{+}$):590.1841, Found: 590.1832.

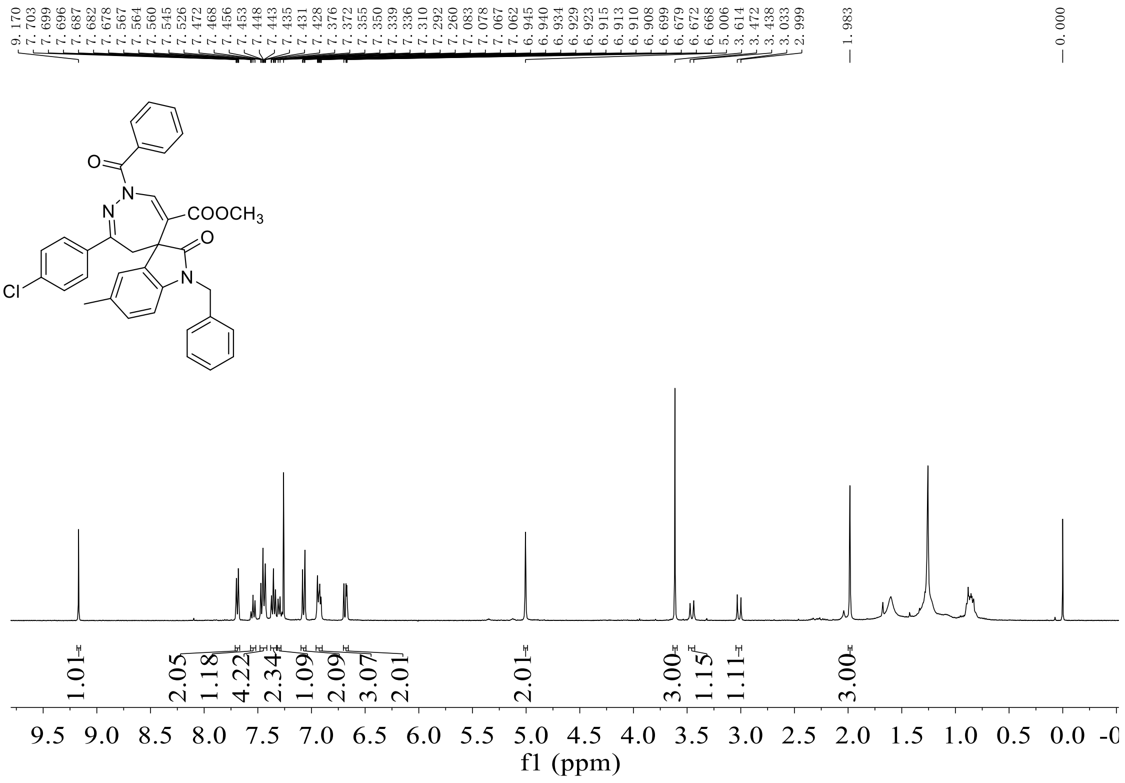

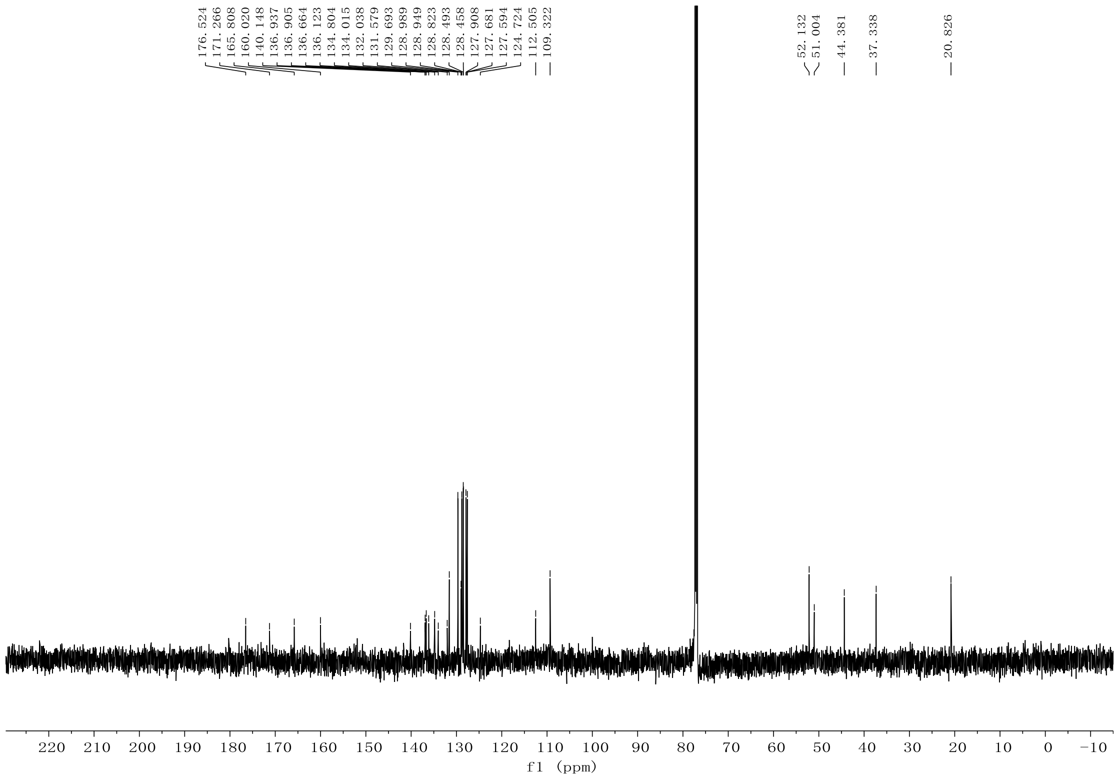
176.524
171.266
165.808
160.020
140.148
136.937
136.905
136.664
136.123
134.804
134.015
132.038
131.579
129.693
128.989
128.949
128.823
128.493
128.458
127.908
127.681
127.594
124.724
112.505
109.322
52.132
51.004
44.381
37.338
20.826
220 210 200 190 180 170 160 150 140 130 120 110 100 90 80 70 60 50 40 30 20 10 0 −10
f1 (ppm)

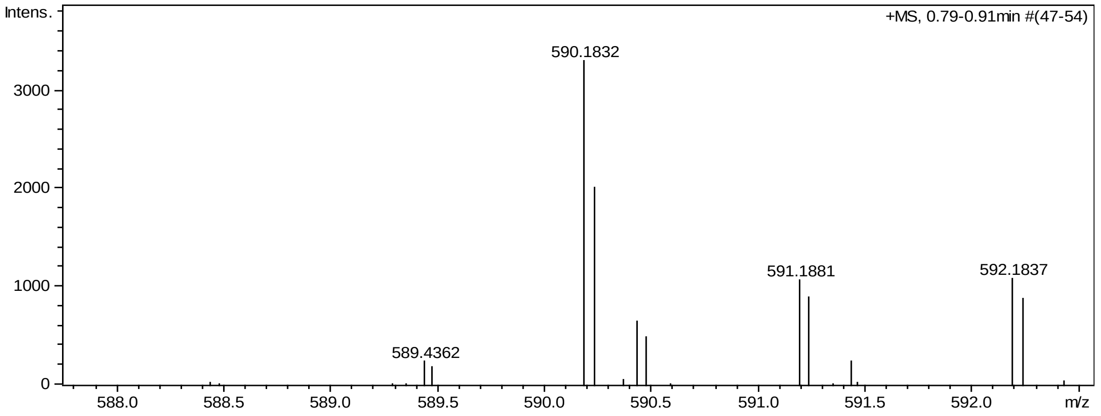
Intens.
+MS, 0.79-0.91min #(47-54)
590.1832
591.1881
592.1837
589.4362
3000
2000
1000
0
588.0
588.5
589.0
589.5
590.0
590.5
591.0
591.5
592.0
m/z

Methyl 1'-benzoyl-1-benzyl-5-chloro-2-oxo-3'-(p-tolyl)-1',4'-dihydrospiro[indoline-3,5'-[1,2]diazepine]-6'-carboxylate (5d): yellow solid, 0.365 g, 65%, m.p. 152-154 ºC; ^{1}H NMR (600 MHz, $CDCl_3$) δ: 9.19 (s, 1H, ArH), 7.71-7.70 (m, 2H, ArH), 7.55-7.52 (m, 1H, ArH), 7.46-7.41 (m, 4H, ArH), 7.39-7.35 (m, 2H, ArH), 7.32-7.29 (m, 1H, ArH), 7.08-7.06 (m, 1H, ArH), 6.95 (s, 4H, ArH), 6.85 (s, 1H, ArH), 6.69-6.68 (m, 1H, ArH), 5.05-4.99 (m, 2H, CH_2), 3.65 (s, 3H, OCH_3), 3.48 (d, J = 13.8 Hz, 1H, CH), 3.08 (d, J = 13.8 Hz, 1H, CH), 2.27 (s, 3H, CH_3) ppm; ^{13}C NMR (100 MHz, $CDCl_3$) δ: 176.3, 171.2, 165.8, 160.7, 141.2, 141.1, 137.5, 135.6, 133.9, 133.2, 133.1, 131.5, 129.7, 129.1, 128.9, 128.4, 127.8, 127.7, 127.5, 127.0, 124.5, 111.5, 110.3, 52.2, 51.1, 44.4, 36.8, 21.3 ppm; IR (KBr) υ: 2925, 2854, 1721, 1608, 1483, 1436, 1342, 1171, 812cm^{-1}; MS (m/z): HRMS (ESI-TOF) Calcd. for $C_{35}H_{29}ClN_3O_4$ ($[M+H]^+$): 590.1841, Found: 590.1849.

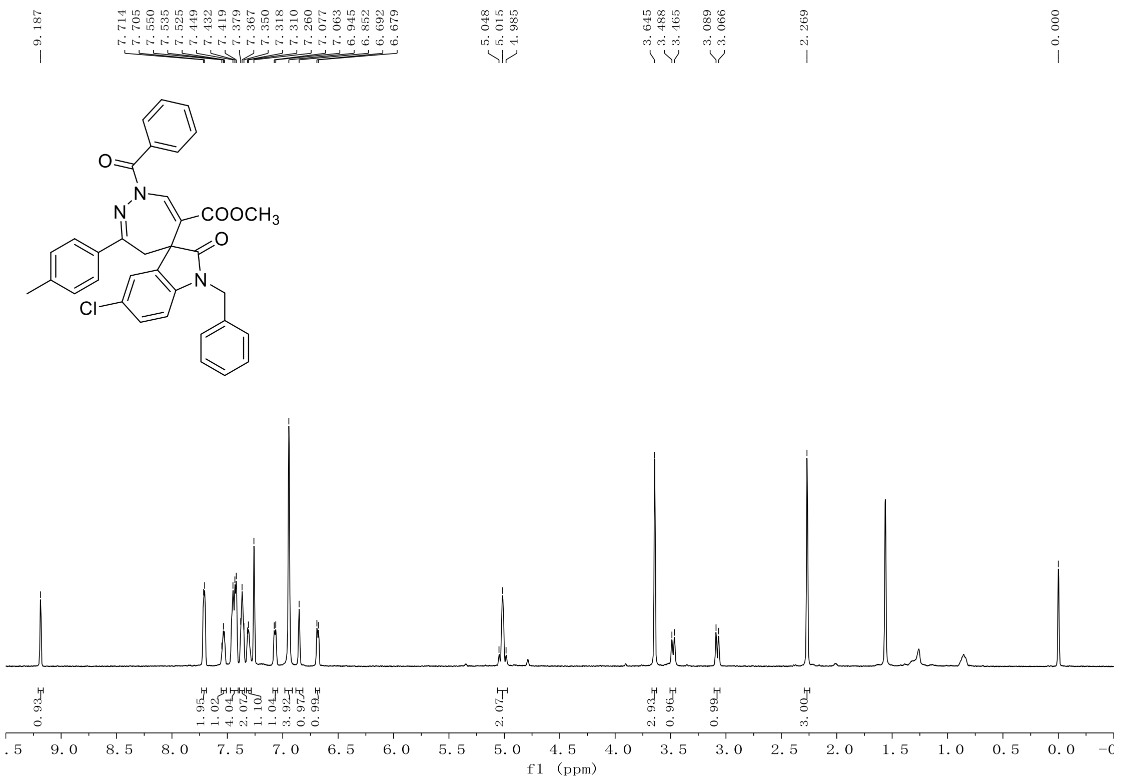

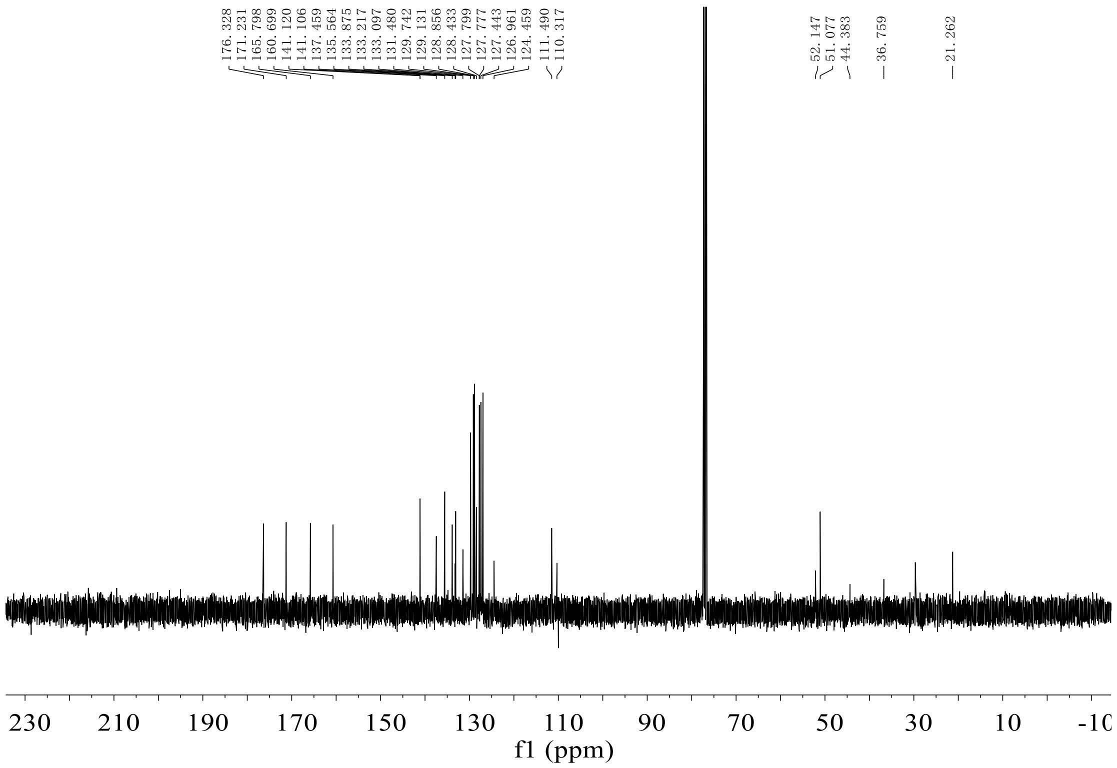
176.328
171.231
165.798
160.699
141.120
141.106
137.459
135.564
133.875
133.217
133.097
131.480
129.742
129.131
128.856
128.433
127.799
127.777
127.443
126.961
124.459
111.490
110.317
52.147
51.077
44.383
36.759
21.262
230 210 190 170 150 130 110 90 70 50 30 10 -10
f1 (ppm)

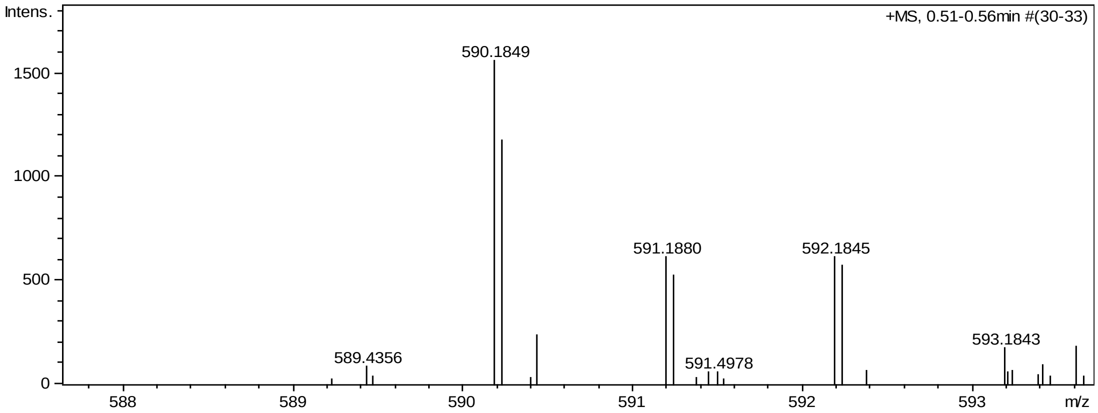
Intens.
+MS, 0.51-0.56min #(30-33)
590.1849
591.1880
592.1845
593.1843
589.4356
591.4978
1500
1000
500
0
588 589 590 591 592 593 m/z

Methyl 1-benzyl-5-methyl-1'-(4-methylbenzoyl)-2-oxo-3'-(p-tolyl)-1',4'-dihydrospiro[indoline-3,5'-[1,2]diazepine]-6'-carboxylate (5e): yellow solid, 0.426 g, 73%, m.p. 201-202 °C; ^{1}H NMR (600 MHz, $CDCl_3$) δ: 9.18 (s, 1H, ArH), 7.64 (d, *J* = 7.2 Hz, 2H, ArH), 7.45-7.43 (m, 2H, ArH), 7.37-7.32 (m, 2H, ArH), 7.30-7.28 (m, 1H, ArH), 7.26-7.24 (m, 2H, ArH), 6.97-6.95 (m, 2H, ArH), 6.94-6.92 (m, 2H, ArH), 6.90-6.88 (m, 1H, ArH), 6.69 (s, 1H, ArH), 6.66-6.65 (m, 1H, ArH), 5.05-4.98 (m, 2H, CH_2), 3.61 (s, 3H, OCH_3), 3.48 (d, *J* = 12.6 Hz, 1H, CH), 3.05 (d, *J* = 13.2 Hz, 1H, CH), 2.43 (s, 3H, CH_3), 2.26 (s, 3H, CH_3), 1.95 (s, 3H, CH_3) ppm; ^{13}C NMR (100 MHz, $CDCl_3$) δ: 176.74, 171.08, 165.95, 161.16, 142.11, 140.65, 140.07, 137.34, 136.37, 136.16, 133.54, 131.82, 131.59, 130.98, 130.12, 128.90, 128.85, 128.72, 128.66, 128.46, 128.12, 127.52, 127.47, 127.13, 126.35, 124.93, 112.04, 109.07, 51.95, 51.01, 44.26, 37.17, 21.64, 21.22, 20.72 ppm; IR (KBr) υ: 2925, 2855, 1716, 1602, 1497, 1449, 1347, 1281, 1189, 1138, 857, 786cm^{-1}; MS (*m*/*z*): HRMS (ESI-TOF) Calcd. for $C_{37}H_{33}N_3O_4Na$ ($[M+Na]^+$): 606.2363, Found: 606.2370.

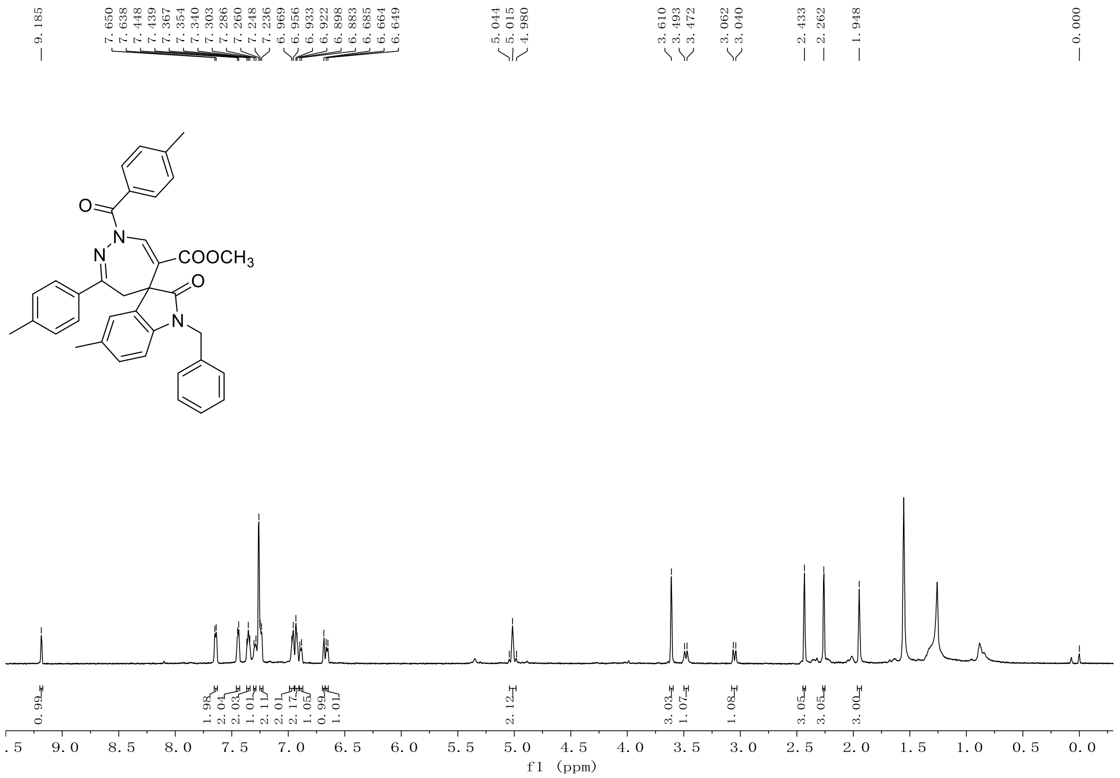

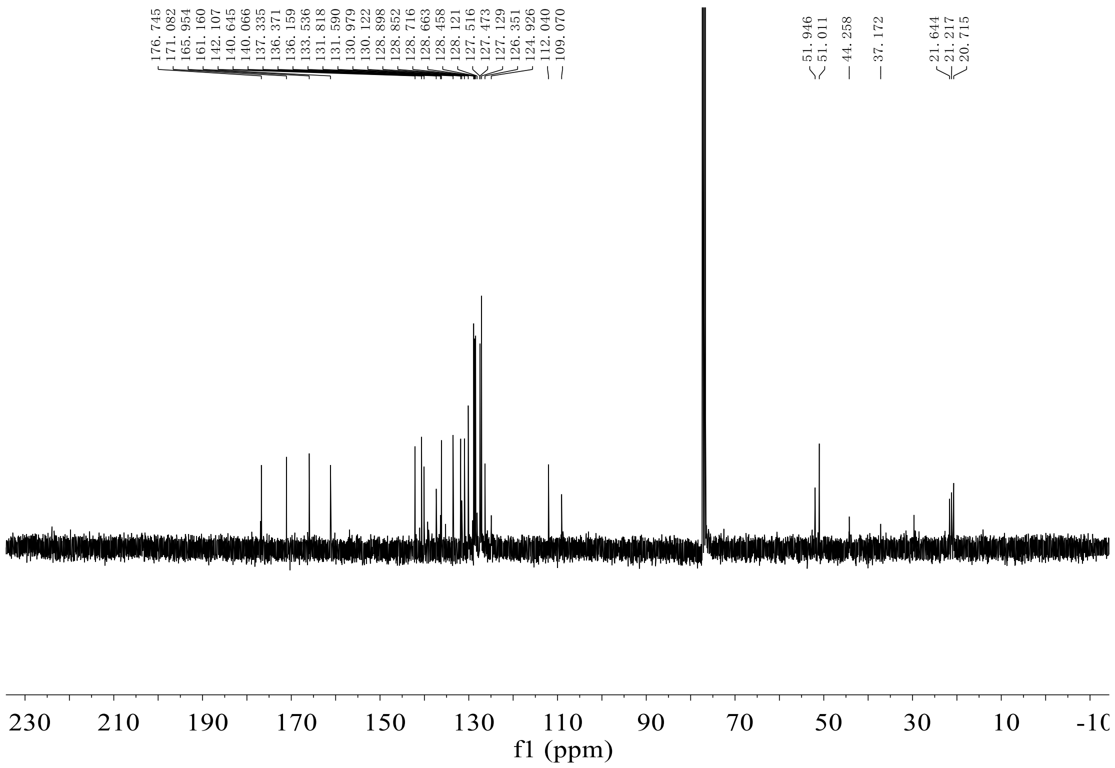
176.745
171.082
165.954
161.160
142.107
140.645
140.066
137.335
136.371
136.159
133.536
131.818
131.590
130.979
130.122
128.898
128.852
128.716
128.663
128.458
128.121
127.516
127.473
127.129
126.351
124.926
112.040
109.070
51.946
51.011
44.258
37.172
21.644
21.217
20.715
230
210
190
170
150
130
110
90
70
50
30
10
-1(
f1 (ppm)

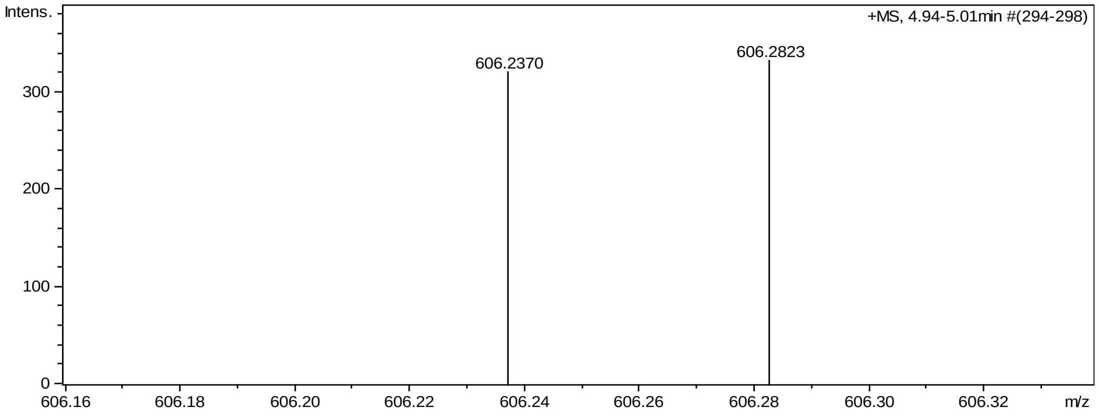
Intens.
+MS, 4.94-5.01min #(294-298)
606.2370
606.2823
300
200
100
0
606.16
606.18
606.20
606.22
606.24
606.26
606.28
606.30
606.32
m/z

Methyl 1'-benzoyl-1-benzyl-5-chloro-3'-(4-methoxyphenyl)-2-oxo-1',4'-dihydrospiro[indoline-3,5'-[1,2]diazepine]-6'-carboxylate (5f): yellow solid, 0.466 g, 77%, m.p. 136-137 ^{o}C; ^{1}H NMR (600 MHz, $CDCl_3$) δ: 9.18 (s, 1H, ArH), 7.71 (d, *J* = 7.2Hz, 2H, ArH), 7.54-7.52 (m, 1H, ArH), 7.47-7.42 (m, 4H, ArH), 7.38-7.36 (m, 2H, ArH), 7.33-7.31 (m, 1H, ArH), 7.08-7.06 (m, 1H, ArH), 7.01-6.99 (m, 2H, ArH), 6.85 (s, 1H, ArH), 6.69 (d, *J* = 7.8Hz, 1H, ArH), 6.64 (d, *J* = 8.4Hz, 2H, ArH), 5.02 (s, 2H, CH_2), 3.75 (s, 3H, OCH_3), 3.65 (s, 3H, OCH_3), 3.48 (d, *J* = 13.2 Hz, 1H, CH), 3.05 (d, *J* = 13.8 Hz, 1H, CH) ppm; ^{13}C NMR (100 MHz, $CDCl_3$) δ: 176.4, 171.2, 165.8, 161.6, 160.5, 141.1, 137.7, 135.6, 134.0, 133.1, 131.5, 129.7, 128.9, 128.7, 128.4, 128.3, 127.8, 127.7, 127.5, 124.5, 113.8, 111.4, 110.3, 55.3, 52.1, 51.1, 44.4, 29.7 ppm; IR (KBr) υ: 2927, 2851, 1718, 1600, 1498, 1444, 1351, 1282, 1189, 1143, 857, 788 cm^{-1}; MS (*m/z*): HRMS (ESI-TOF) Calcd. for $C_{35}H_{29}ClN_3O_5$ ($[M+H]^+$): 606.1790, Found: 606.1747.

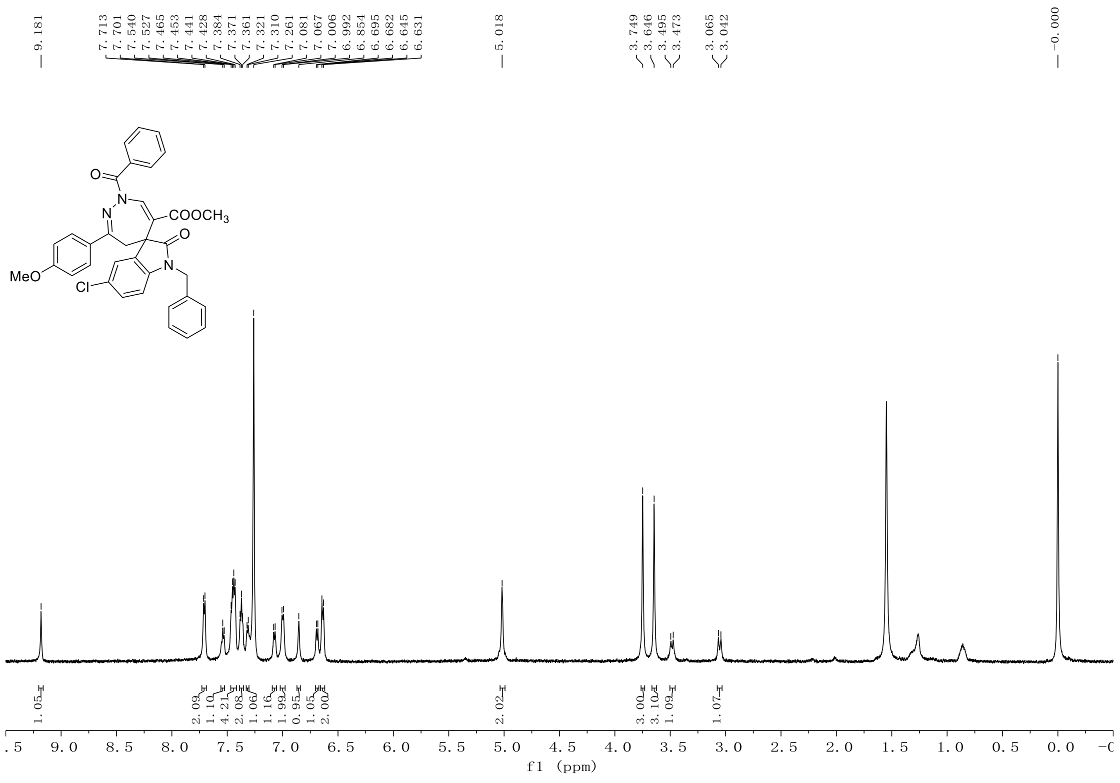

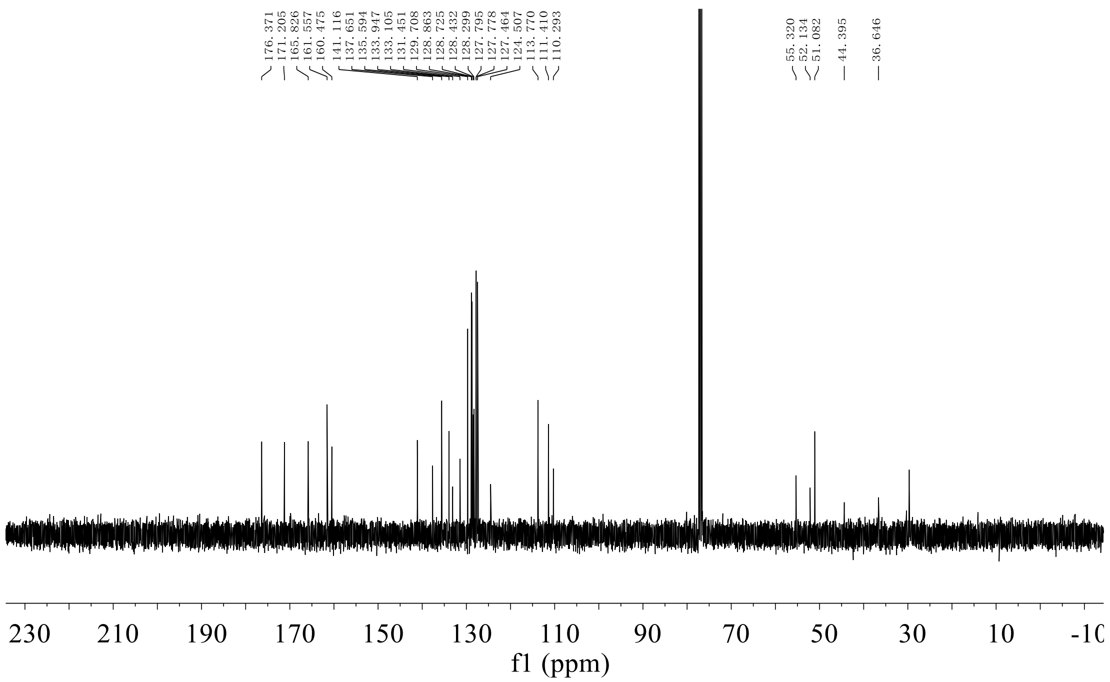
176.371
171.205
165.826
161.557
160.475
141.116
137.651
135.594
133.947
133.105
131.451
129.708
128.863
128.725
128.432
128.299
127.795
127.778
127.464
124.507
113.770
111.410
110.293
55.320
52.134
51.082
44.395
36.646
230
210
190
170
150
130
110
90
70
50
30
10
-10
f1 (ppm)

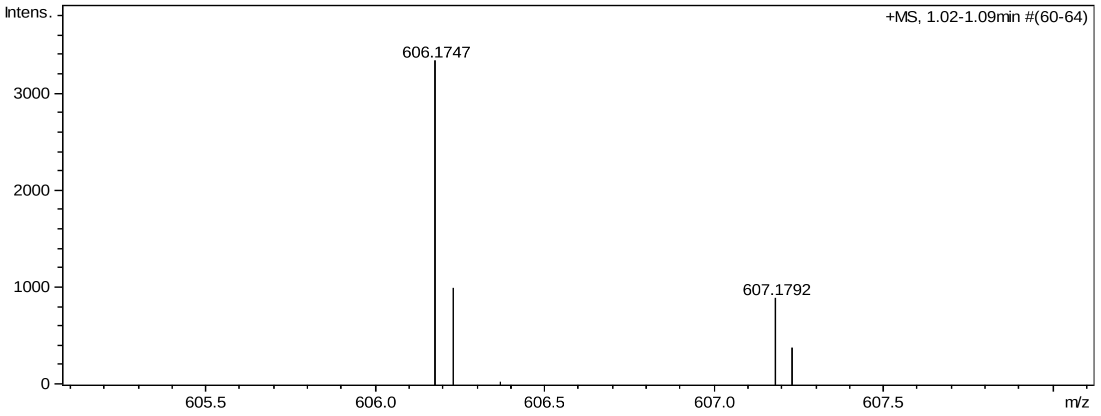
Intens.
+MS, 1.02-1.09min #(60-64)
606.1747
607.1792
3000
2000
1000
0
605.5
606.0
606.5
607.0
607.5
m/z

Methyl 1'-benzoyl-1-benzyl-3'-(4-methoxyphenyl)-5-methyl-2-oxo-1',4'-dihydrospiro[indoline-3,5'-[1,2]diazepine]-6'-carboxylate (5g): yellow solid, 0.356 g, 63%, m.p. 184-187 °C; ^{1}H NMR (400 MHz, $CDCl_3$) δ: 9.17 (s, 1H, ArH), 7.71-7.69 (m, 2H, ArH), 7.55-7.52 (m, 1H, ArH), 7.47-7.44 (m, 4H, ArH), 7.38-7.34 (m, 2H, ArH), 7.31-7.29 (m, 1H, ArH), 6.95 (d, J = 8.8 Hz, 2H, ArH), 6.90 (d, J = 8.0 Hz, 1H, ArH), 6.69-6.66 (m, 2H, ArH), 6.60 (d, J = 8.8 Hz, 2H, ArH), 5.02 (s, 2H, CH_2), 3.73 (s, 3H, OCH_3), 3.62 (s, 3H, OCH_3), 3.50 (d, J = 13.2 Hz, 1H, CH), 3.03 (d, J = 13.2 Hz, 1H, CH), 1.95 (s, 3H, CH_3) ppm; ^{13}C NMR (100 MHz, $CDCl_3$) δ: 176.3, 171.3, 165.8, 160.6, 141.1, 137.3, 135.9, 135.5, 133.8, 133.2, 131.6, 130.6, 129.7, 128.9, 128.7, 128.5, 128.4, 127.9, 127.8, 127.5, 127.0, 124.4, 111.6, 110.4, 52.2, 52.2, 51.1, 44.4, 36.9 ppm; IR (KBr) υ: 2923, 2851, 1716, 1603, 1497, 1438, 1348, 1282, 1189, 1142, 857, 783 cm^{-1}; MS (m/z): HRMS (ESI-TOF) Calcd. for $C_{36}H_{32}N_3O_5$ ([M+H]$^{+}$): 586.2336, Found: 586.2325.

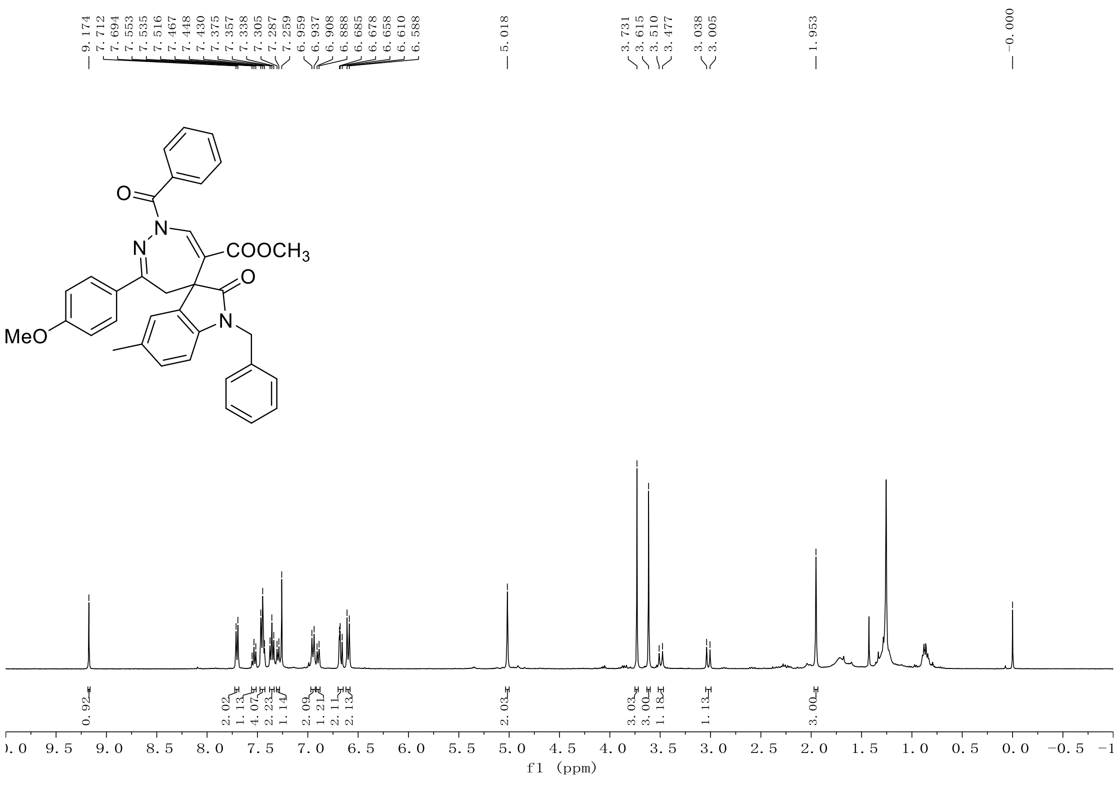

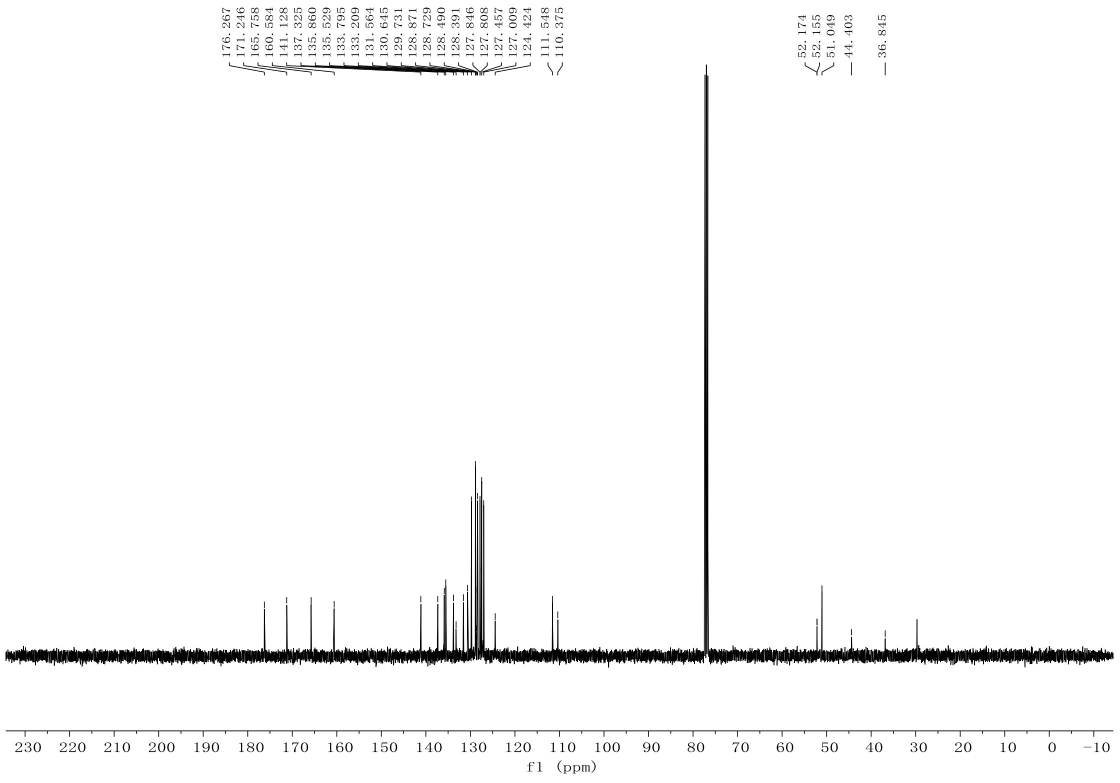
176.267
171.246
165.758
160.584
141.128
137.325
135.860
135.529
133.795
133.209
131.564
130.645
129.731
128.871
128.729
128.490
128.391
127.846
127.808
127.457
127.009
124.424
111.548
110.375
52.174
52.155
51.049
44.403
36.845
f1 (ppm)

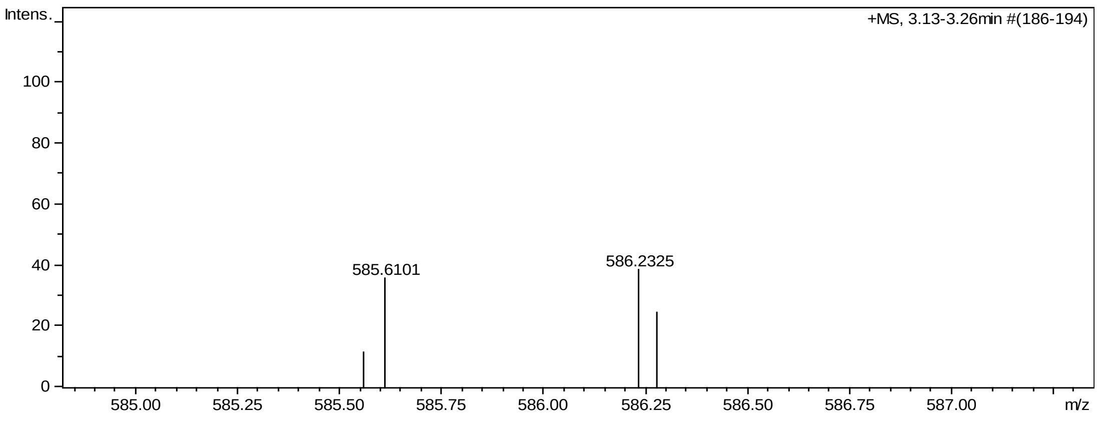
Intens.
+MS, 3.13-3.26min #(186-194)
585.6101
586.2325
m/z

1-Benzyl-2-oxo-3'-(p-tolyl)-1'-tosyl-1',4'-dihydrospiro[indoline-3,5'-[1,2]diazepine]-6'-carbonitrile (7a): white solid, 64%, m.p. 226-230 ºC; ^{1}H NMR (400 MHz, $CDCl_3$) δ: 8.10 (s, 1H, C=CH-N), 7.97 (d, *J* = 8.0 Hz, 2H, ArH), 7.42 (d, *J* = 8.0 Hz, 2H, ArH), 7.29 (d, *J* = 8.4 Hz, 2H, ArH), 7.28-7.26 (s, 1H, ArH), 7.26-7.24 (m, 2H, ArH), 7.22-7.20 (m, 1H, ArH), 7.19-7.16 (m, 2H, ArH), 7.09 (s, 1H, ArH), 7.06 (d, *J* = 4.8 Hz, 2H, ArH), 6.98 (t, *J* = 7.6 Hz, 1H, ArH), 6.78 (d, *J* = 7.6 Hz, 1H, ArH), 4.88 (d, *J* = 15.6 Hz, 1H, CH), 4.79 (d, *J* = 15.6 Hz, 1H, CH), 3.29 (d, *J* = 10.0 Hz, 1H, CH), 3.20 (d, *J* = 10.0 Hz, 1H, CH), 2.49 (s, 3H, CH_3), 2.34 (s, 3H, CH_3) ppm; ^{13}C {^{1}H} NMR (100 MHz, $CDCl_3$) δ: 173.7, 160.5, 145.9, 141.8, 141.1, 139.4, 135.1, 133.5, 132.7, 129.9, 129.9, 129.5, 129.2, 129.0, 128.8, 127.8, 127.3, 127.2, 124.5, 123.5, 117.3, 109.9, 91.9, 52.1, 44.3, 37.3, 21.8, 21.3 ppm; IR (KBr) υ: 3057, 3055, 2928, 2217, 1716, 1613, 1488, 1467, 1449, 1369, 1297, 1259, 1189, 1175, 1090, 1021, 999, 869, 804, 764, 754, 696, 667, 657, 639 cm^{-1}; MS (*m/z*): HRMS (ESI-TOF) Calcd. for $C_{34}H_{28}ClN_4O_3SNa$ ([M+Na]$^{+}$): 595.1774, Found: 595.1765.

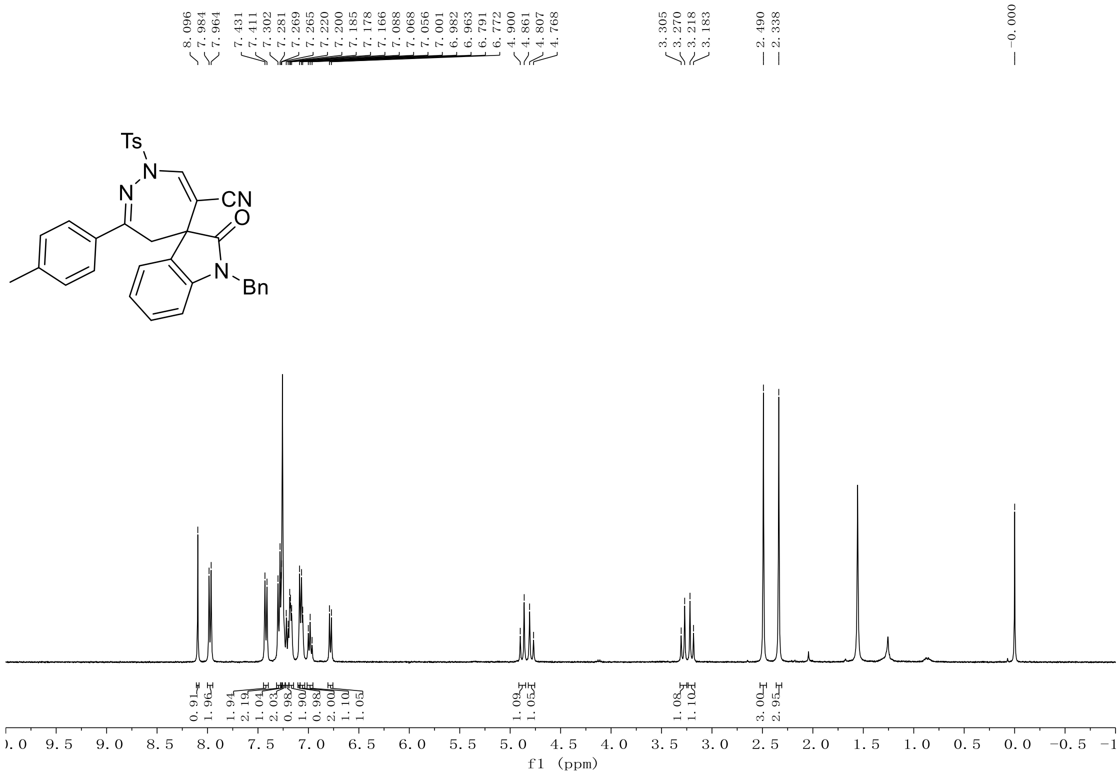

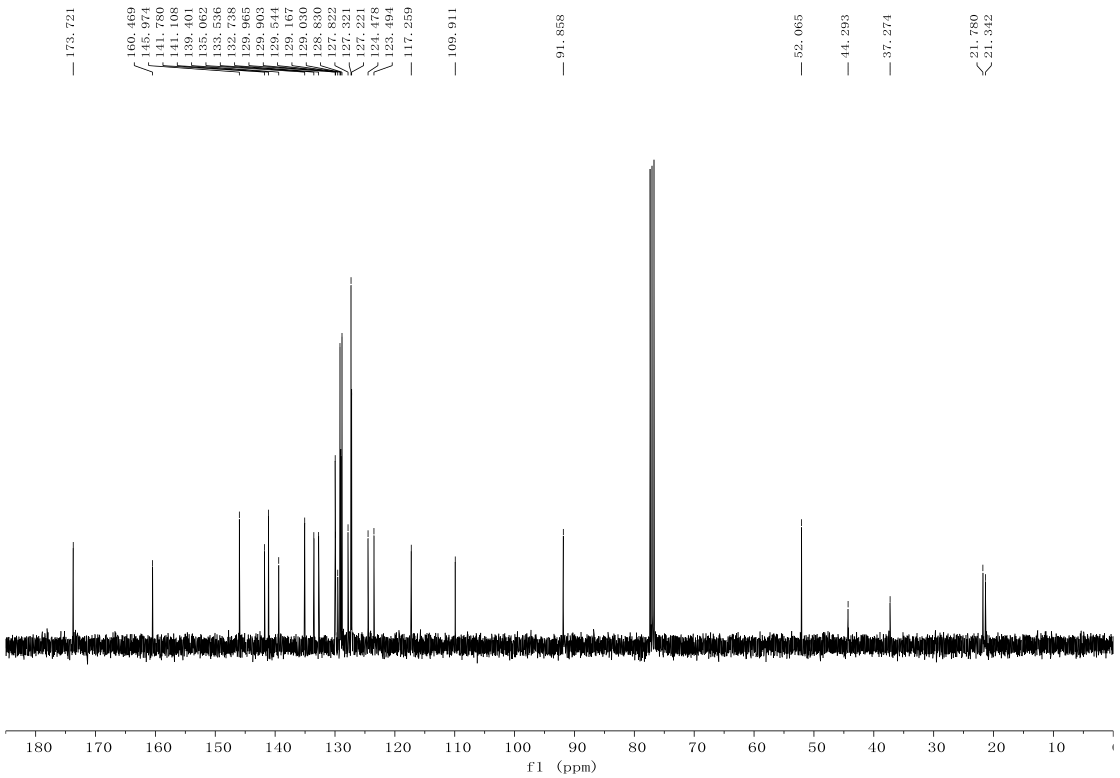

173.721
160.469
145.974
141.780
141.108
139.401
135.062
133.536
132.738
129.965
129.903
129.544
129.167
129.030
128.830
127.822
127.321
127.221
124.478
123.494
117.259
109.911
91.858
52.065
44.293
37.274
21.780
21.342
180 170 160 150 140 130 120 110 100 90 80 70 60 50 40 30 20 10
f1 (ppm)

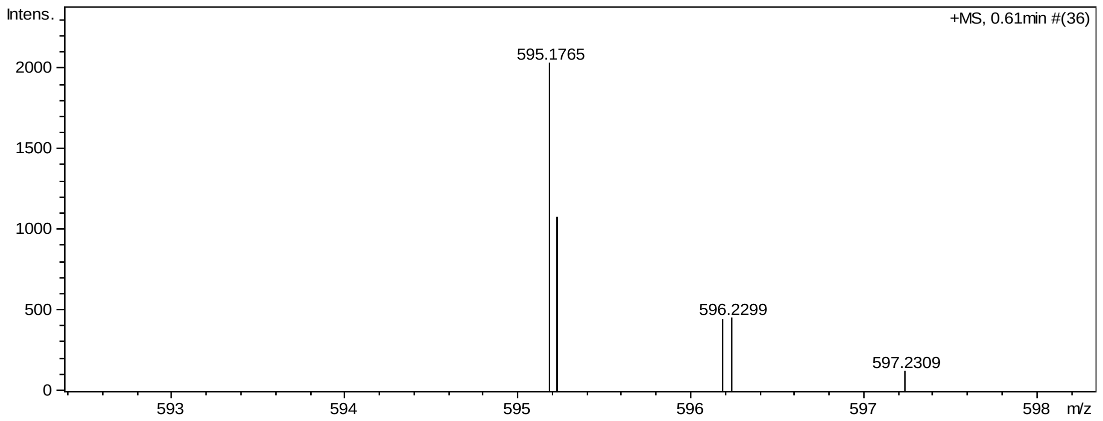

Intens.
+MS, 0.61min #(36)
595.1765
596.2299
597.2309
2000
1500
1000
500
0
593
594
595
596
597
598
m/z

1-Butyl-5-chloro-2-oxo-3'-phenyl-1'-tosyl-1',4'-dihydrospiro[indoline-3,5'-[1,2]diazepine]-6'-carbonitrile (7b): white solid, 76%, m.p. 224-228 ºC; ^{1}H NMR (400 MHz, $CDCl_3$) δ: 8.06 (s, 1H, C=CH-N), 7.97 (d, *J* = 8.4 Hz, 2H, ArH), 7.45-7.42 (m, 3H, ArH), 7.41-7.40 (m, 1H, ArH), 7.39-7.37 (m, 1H, ArH), 7.33-7.31 (m, 1H, ArH), 7.30-7.29 (m, 1H, ArH), 7.28-7.27 (m, 1H, ArH), 7.10 (d, *J* = 2.0 Hz, 1H, ArH), 6.82 (d, *J* = 8.4 Hz, 1H, ArH), 3.65-3.60 (m, 2H, CH_2), 3.28 (d, *J* = 14.0 Hz, 1H, CH), 3.16 (d, *J* = 13.6 Hz, 1H, CH), 2.50 (s, 3H, CH_3), 1.60-1.53 (m, 2H, CH_2), 1.23 (t, *J* = 8.0 Hz, 2H, CH_2), 0.86 (t, *J* = 7.2 Hz, 3H, CH_3) ppm; ^{13}C {^{1}H} NMR (100 MHz, $CDCl_3$) δ: 173.1, 160.2, 146.2, 140.8, 139.4, 136.2, 132.6, 131.0, 130.8, 130.0, 129.9, 129.0, 128.6, 128.5, 127.1, 125.1, 116.8, 110.2, 91.5, 51.9, 40.4, 36.8, 29.1, 21.8, 19.9, 13.6 ppm; IR (KBr) υ: 3061, 2957, 2932, 2872, 2215, 1719, 1618, 1595, 1483, 1446, 1428, 1370, 1296, 1189, 1174, 1109, 1088, 994, 886, 813, 759, 698, 658, 606 cm^{-1}; MS (*m*/*z*): HRMS (ESI-TOF) Calcd. for $C_{30}H_{27}ClN_4O_3SNa$ ($[M+Na]^+$): 581.1385, Found: 581.1386.

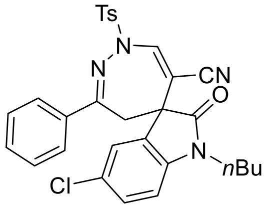

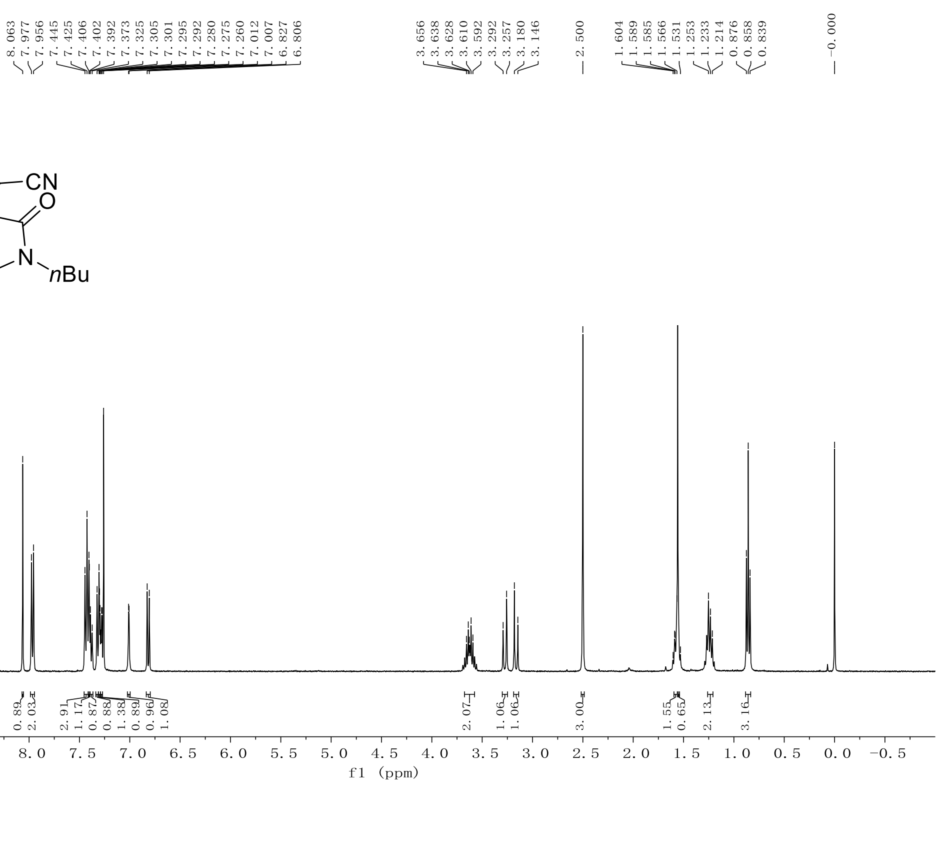

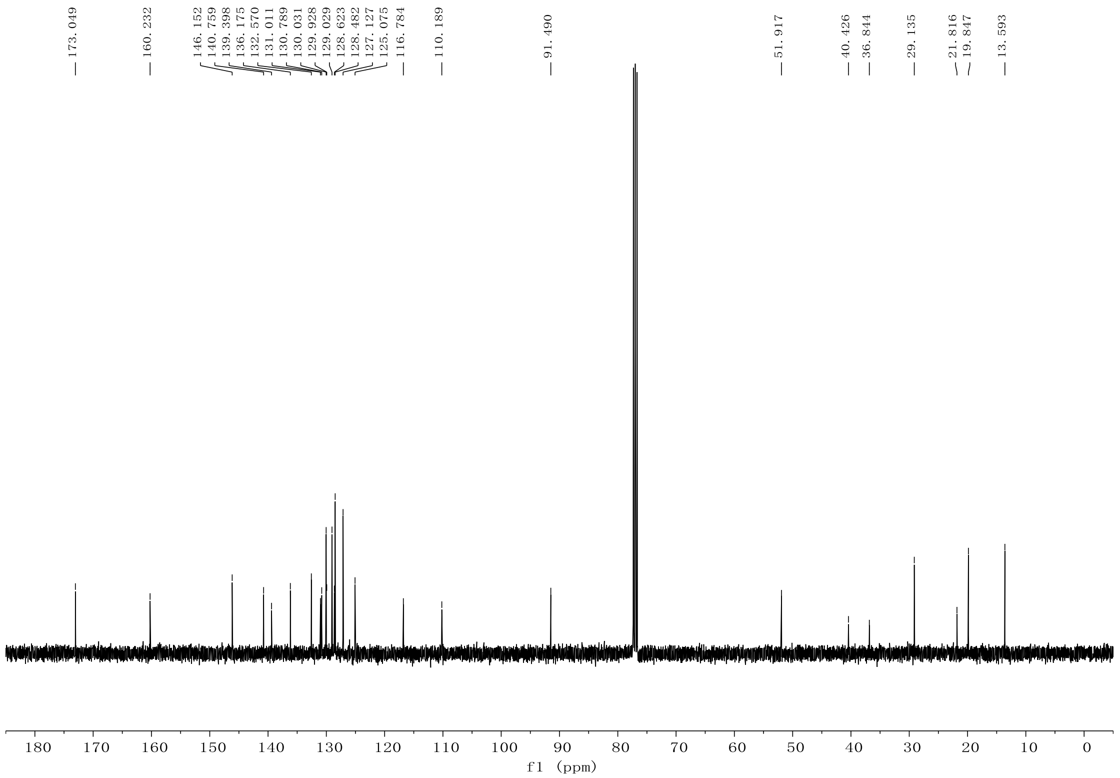
173.049
160.232
146.152
140.759
139.398
136.175
132.570
131.011
130.789
130.031
129.928
129.029
128.623
128.482
127.127
125.075
116.784
110.189
91.490
51.917
40.426
36.844
29.135
21.816
19.847
13.593
f1 (ppm)

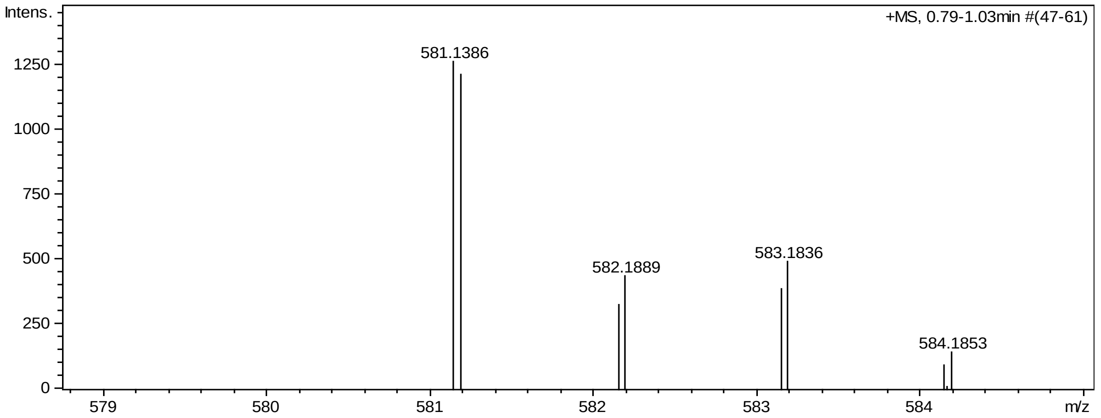
Intens.
+MS, 0.79-1.03min #(47-61)
581.1386
582.1889
583.1836
584.1853
m/z

1-Butyl-5-chloro-3'-(4-chlorophenyl)-2-oxo-1'-tosyl-1',4'-dihydrospiro[indoline-3,5'-[1,2]diazepine]-6'-carbonitrile (7c): white solid, 83%, m.p. 208-213 ºC; ^{1}H NMR (400 MHz, $CDCl_3$) δ: 8.06 (s, 1H, C=CH-N), 7.94 (d, *J* = 8.4 Hz, 2H, ArH), 7.43 (d, *J* = 8.0 Hz, 2H, ArH), 7.36-7.34 (m, 2H, ArH), 7.33-7.31 (m, 1H, ArH), 7.30-7.29 (m, 1H, ArH), 7.28-7.27 (m, 1H, ArH), 7.04 (d, *J* = 2.0 Hz, 1H, ArH), 6.83 (d, *J* = 8.4 Hz, 1H, ArH), 3.67-3.57 (m, 2H, CH_2), 3.20 (d, *J* = 14.0 Hz, 1H, CH), 3.15 (d, *J* = 13.6 Hz, 1H, CH), 2.50 (s, 3H, CH_3), 1.60-1.53 (m, 2H, CH_2), 1.26-1.20 (m, 2H, CH_2), 0.86 (t, *J* = 7.6 Hz, 3H, CH_3) ppm; ^{13}C {^{1}H} NMR (100 MHz, $CDCl_3$) δ: 173.0, 159.0, 146.3, 140.7, 139.2, 137.1, 134.8, 132.5, 131.0, 130.1, 129.0, 128.8, 128.4, 125.0, 116.7, 110.3, 91.6, 51.9, 40.5, 36.8, 29.1, 21.8, 19.8, 13.6 ppm; IR (KBr) υ: 3072, 2958, 2931, 2871, 2213, 1715, 1625, 1594, 1484, 1429, 1398, 1362, 1292, 1263, 1230, 1212, 1189, 1174, 1142, 1108, 1089, 1011, 999, 890, 831, 812, 761, 750, 728, 715, 701, 682, 660, 584 cm^{-1}; MS (*m*/*z*): HRMS (ESI-TOF) Calcd. for $C_{30}H_{26}ClN_4O_3SNa$ ($[M+Na]^+$): 615.0995, Found: 615.0996.

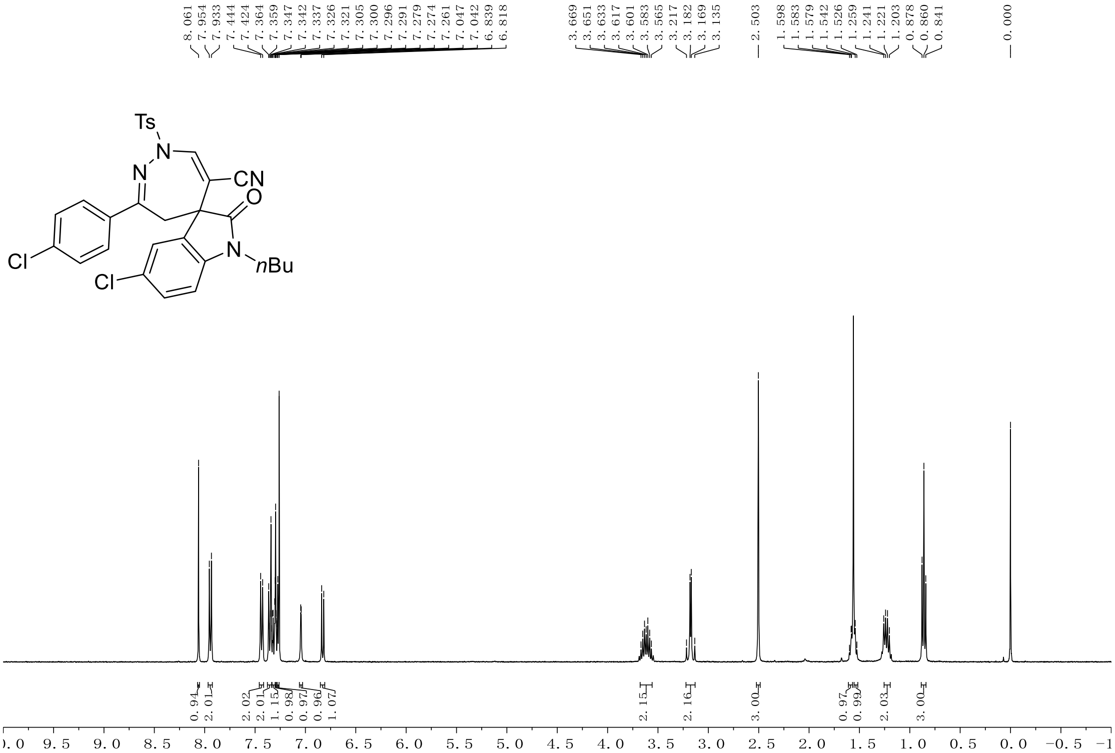

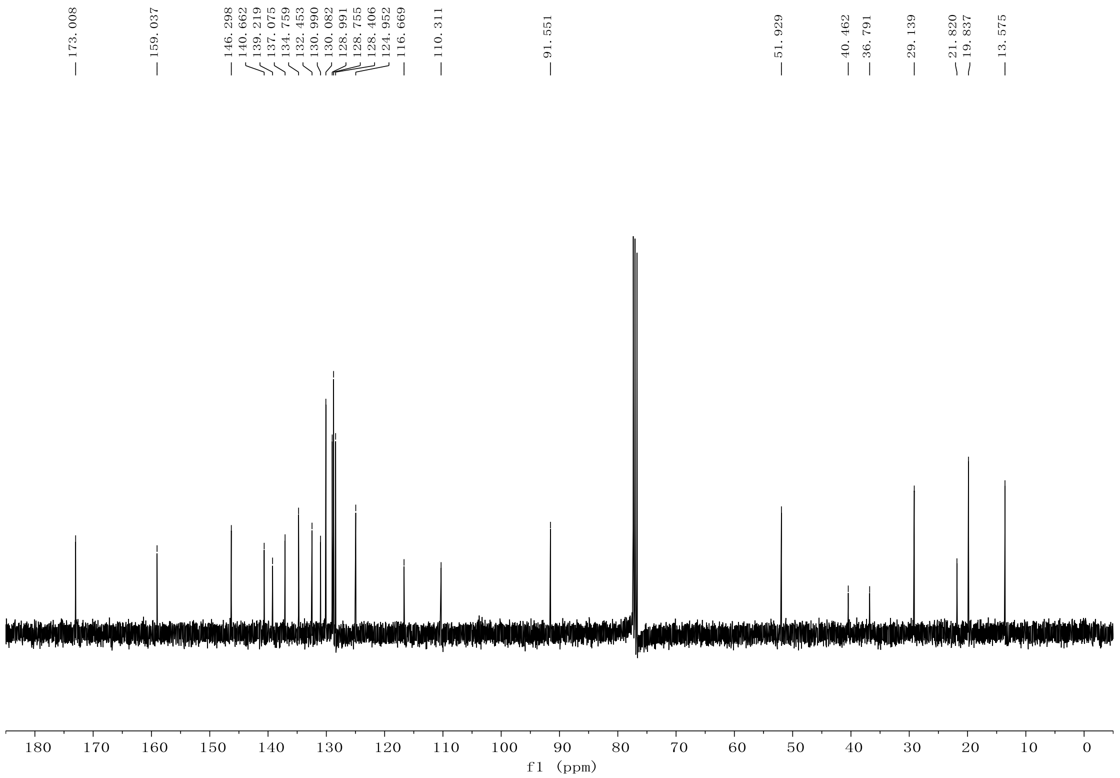
173.008
159.037
146.298
140.662
139.219
137.075
134.759
132.453
130.990
130.082
128.991
128.755
128.406
124.952
116.669
110.311
91.551
51.929
40.462
36.791
29.139
21.820
19.837
13.575
180 170 160 150 140 130 120 110 100 90 80 70 60 50 40 30 20 10 0
f1 (ppm)

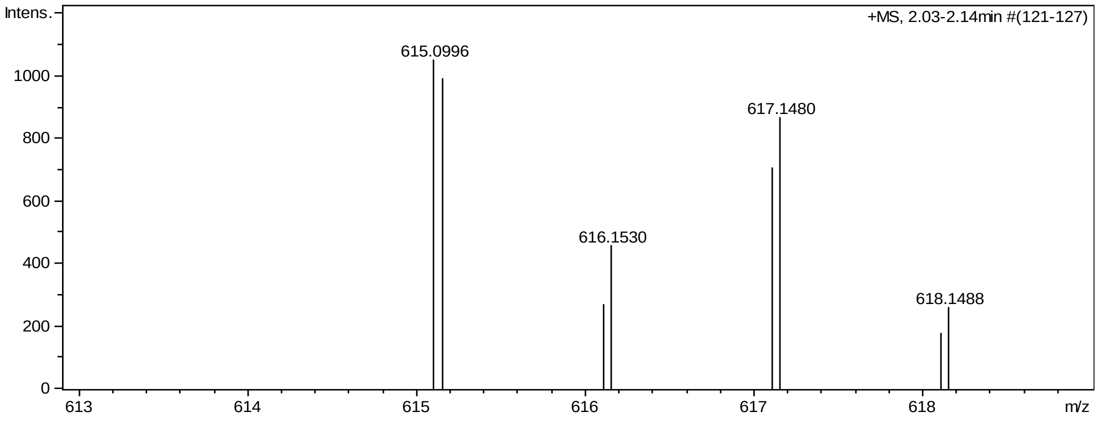
Intens.
+MS, 2.03-2.14min #(121-127)
615.0996
617.1480
616.1530
618.1488
1000
800
600
400
200
0
613
614
615
616
617
618
m/z

1-Benzyl-5-methyl-2-oxo-3'-phenyl-1'-tosyl-1',4'-dihydrospiro[indoline-3,5'-[1,2]diazepine]-6'-carbonitrile (7d): white solid, 64%, m.p. 246-249 ºC; ^{1}H NMR (400 MHz, $CDCl_3$) δ: 8.10 (s, 1H, C=CH-N), 7.98 (d, *J* = 8.0 Hz, 2H, ArH), 7.44-7.40 (m, 4H, ArH), 7.38-7.36 (m, 1H, ArH), 7.29 (d, *J* = 7.6 Hz, 2H, ArH), 7.25-7.23 (m, 3H, ArH), 7.15-7.13 (m, 2H, ArH), 7.02 (d, *J* = 8.0 Hz, 2H, ArH), 6.91 (s, 1H, ArH), 6.67 (d, *J* = 8.0 Hz, 2H, ArH), 4.85 (d, *J* = 15.6 Hz, 1H, CH), 4.75 (d, *J* = 16.0 Hz, 1H, CH), 3.26 (d, *J* = 14.4 Hz, 1H, CH), 3.22 (d, *J* = 14.4 Hz, 1H, CH), 2.49 (s, 3H, CH_3), 2.21 (s, 3H, CH_3) ppm; ^{13}C {^{1}H} NMR (100 MHz, $CDCl_3$) δ: 173.7, 160.5, 146.0, 139.3, 139.2, 136.5, 135.2, 133.3, 132.7, 130.6, 130.2, 130.0, 129.6, 129.0, 128.8, 128.4, 127.8, 127.3, 127.3, 125.1, 117.3, 109.7, 92.2, 52.2, 44.3, 37.5, 21.8, 21.0 ppm; IR (KBr) υ: 3057, 2923, 2214, 1714, 1618, 1603, 1495, 1446, 1370, 1283, 1234, 1189, 1175, 1089, 1029, 995, 879, 810, 762, 740, 696, 659, 611, 595, 577, 547, 529, 466 cm^{-1}; MS (*m*/*z*): HRMS (ESI-TOF) Calcd. for $C_{34}H_{28}ClN_4O_3SNa$ ($[M+Na]^+$): 595.1774, Found: 595.1776.

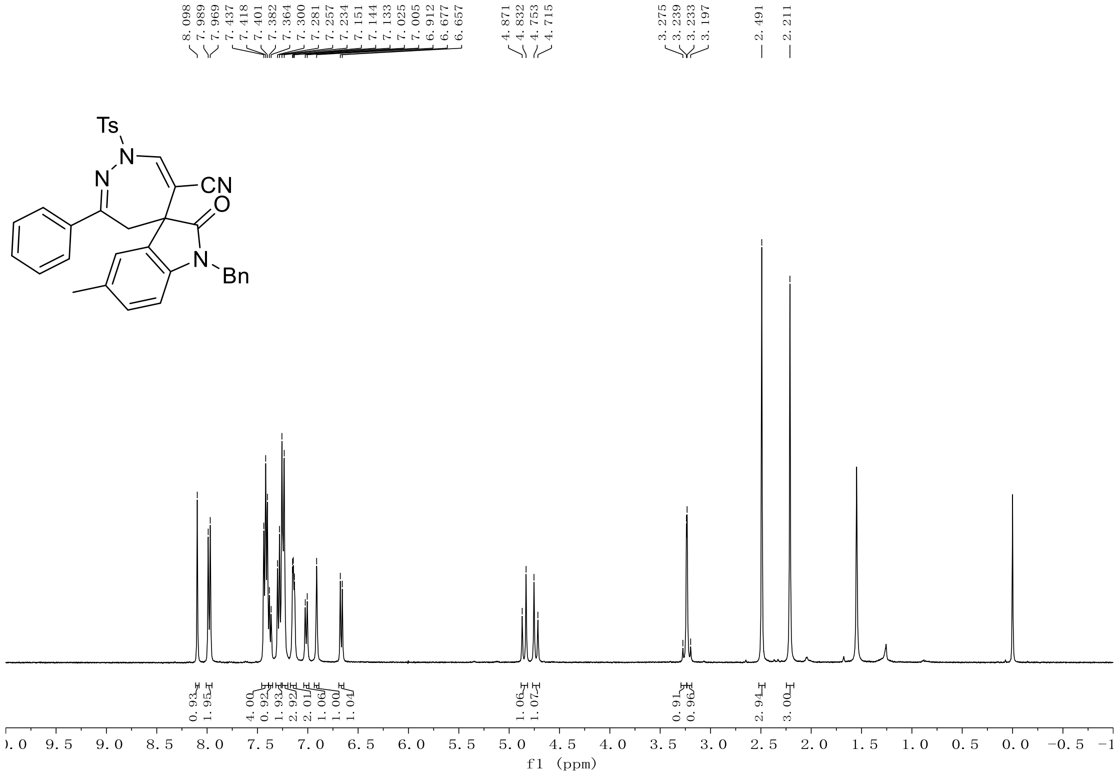

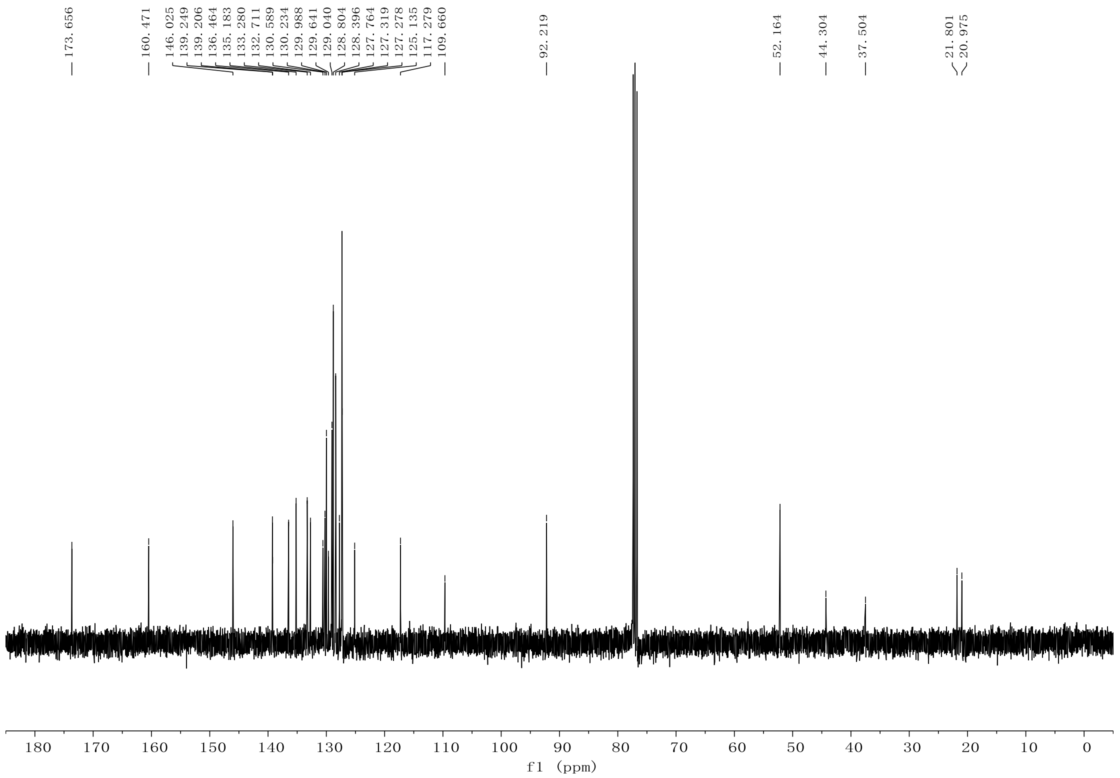
173.656
160.471
146.025
139.249
139.206
136.464
135.183
133.280
132.711
130.589
130.234
129.988
129.641
129.040
128.804
128.396
127.764
127.319
127.278
125.135
117.279
109.660
92.219
52.164
44.304
37.504
21.801
20.975
180 170 160 150 140 130 120 110 100 90 80 70 60 50 40 30 20 10 0
f1 (ppm)

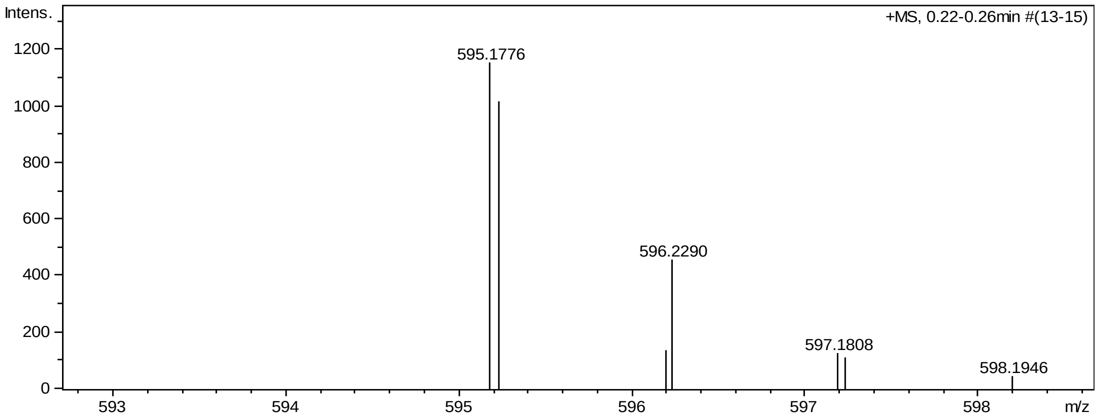
Intens.
+MS, 0.22-0.26min #(13-15)
1200
1000
800
600
400
200
0
595.1776
596.2290
597.1808
598.1946
593 594 595 596 597 598 m/z

1-Benzyl-3'-(4-chlorophenyl)-5-fluoro-2-oxo-1'-tosyl-1',4'-dihydrospiro[indoline-3,5'-[1,2]diazepine]-6'-carbonitrile (7e): white solid, 60%, m.p. 175-179 ºC; ^{1}H NMR (400 MHz, $CDCl_3$) δ: 8.11 (s, 1H, C=CH-N), 7.95 (d, *J* = 7.6 Hz, 2H, ArH), 7.43 (d, *J* = 7.6 Hz, 2H, ArH), 7.33 (d, *J* = 7.6 Hz, 2H, ArH), 7.27-7.26 (m, 4H, ArH), 7.25-7.24 (m, 1H, ArH), 7.14 (s, 2H, ArH), 6.95 (t, *J* = 8.0 Hz, 1H, ArH), 6.83 (d, *J* = 4.2 Hz, 1H, ArH), 6.75-6.73 (m, 1H, ArH), 4.85 (d, *J* = 15.6 Hz, 1H, CH), 4.77 (d, *J* = 15.6 Hz, 1H, CH), 3.24-3.17 (m, 2H, CH_2), 2.50 (s, 3H, CH_3) ppm; ^{13}C {^{1}H} NMR (100 MHz, $CDCl_3$) δ: 173.4, 159.4 (d, *J* = 243 Hz), 158.2, 146.3, 139.3, 137.7, 137.6, 137.1, 134.7, 134.7, 132.4, 130.8 (d, *J* = 7.6 Hz), 130.1, 129.0, 129.0, 128.8, 128.5, 128.1, 127.3, 116.9, 116.7 (d, *J* = 23.5 Hz), 112.5 (d, *J* = 24.6 Hz), 110.8 (d, *J* = 8.1 Hz), 91.2, 52.3, 44.5, 37.2, 21.8 ppm; IR (KBr) υ: 3063, 2921, 2212, 1718, 1619, 1594, 1492, 1454, 1437, 1400, 1360, 1292, 1268, 1234, 1215, 1188, 1173, 1088, 1011, 1001, 991, 887, 839, 828, 812, 772, 729, 701, 687, 660 cm^{-1}; MS (*m/z*): HRMS (ESI-TOF) Calcd. for $C_{33}H_{24}ClFN_4O_3SNa$ ($[M+Na]^+$): 633.1134, Found: 633.1133.

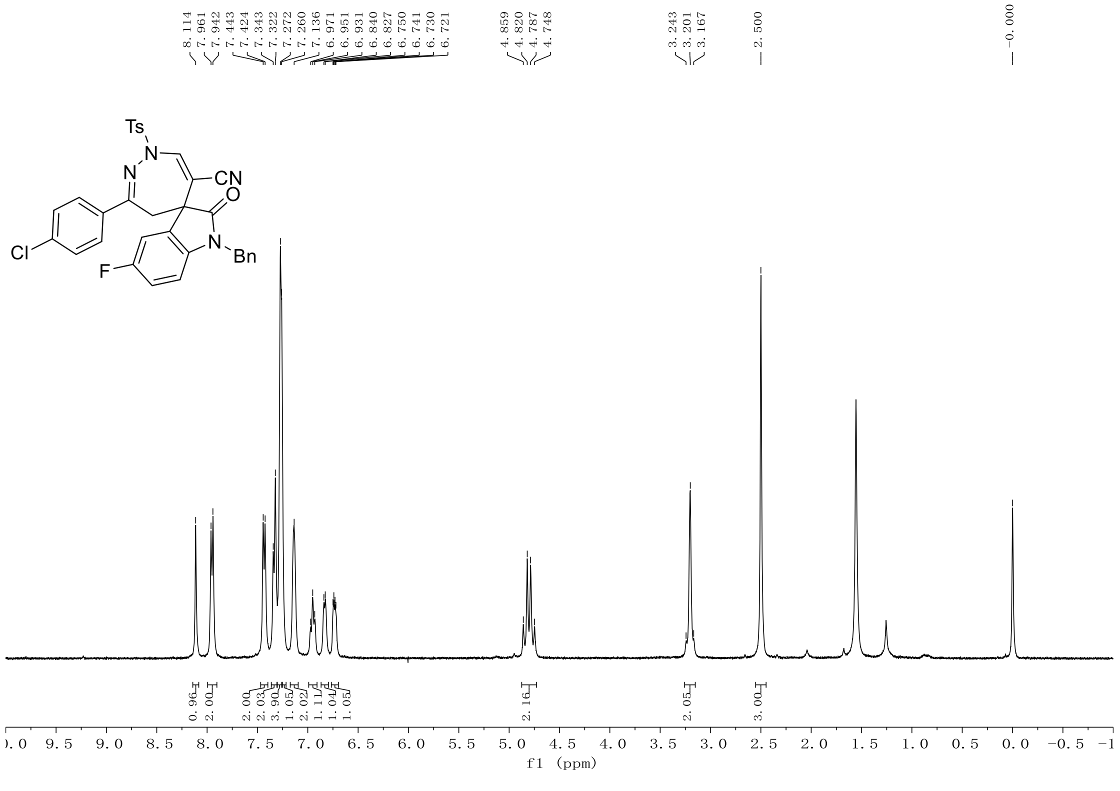

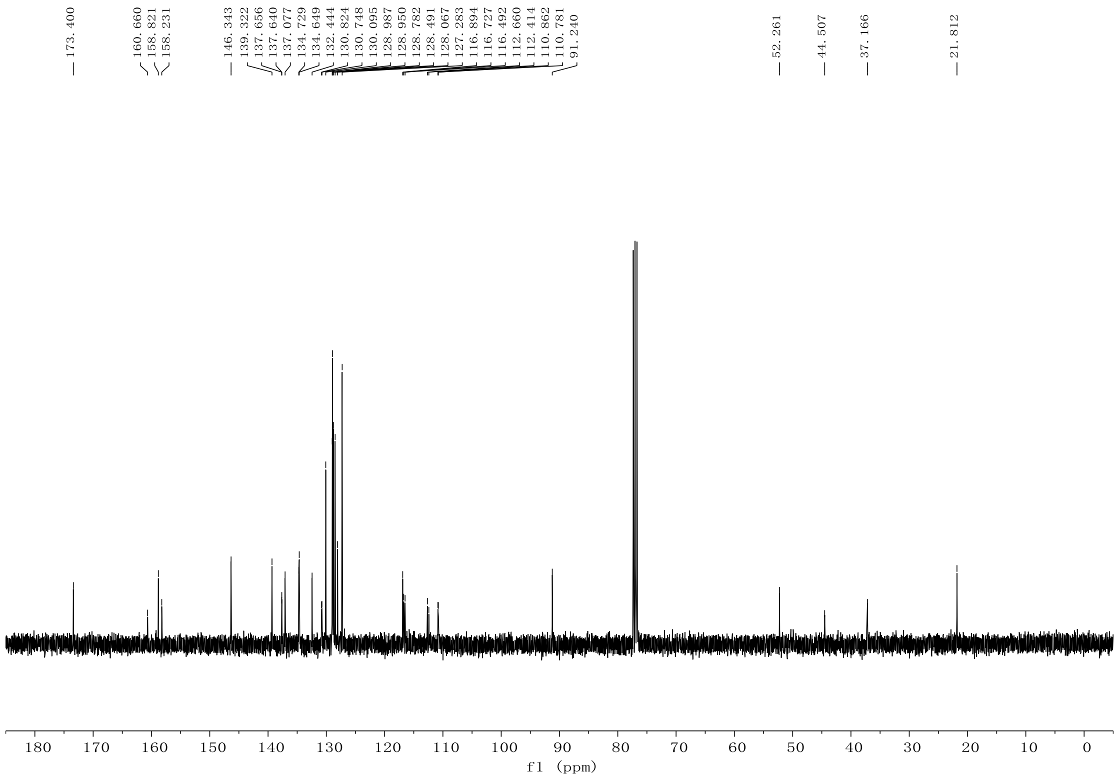
173.400
160.660
158.821
158.231
146.343
139.322
137.656
137.640
137.077
134.729
134.649
132.444
130.824
130.748
130.095
128.987
128.950
128.782
128.491
128.067
127.283
116.894
116.727
116.492
112.660
112.414
110.862
110.781
91.240
52.261
44.507
37.166
21.812
f1 (ppm)

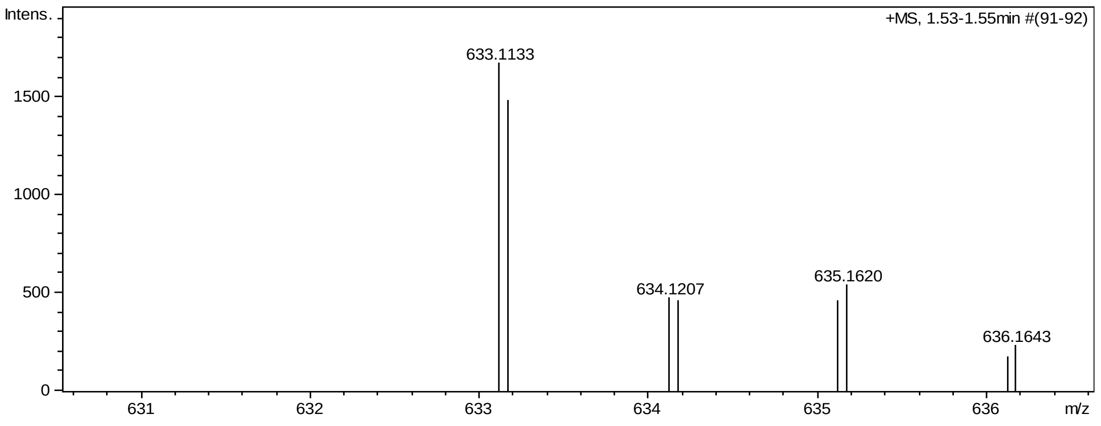
Intens.
+MS, 1.53-1.55min #(91-92)
633.1133
634.1207
635.1620
636.1643
m/z

1-Butyl-5-chloro-3'-(4-methoxyphenyl)-2-oxo-1'-tosyl-1',4'-dihydrospiro[indoline-3,5'-[1,2]diazepine]-6'-carbonitrile (7f): white solid, 79%, m.p. 223-227 ºC; ^{1}H NMR (400 MHz, $CDCl_3$) δ: 8.04 (s, 1H, C=CH-N), 7.96 (d, *J* = 8.4 Hz, 2H, ArH), 7.43 (d, *J* = 8.0 Hz, 2H, ArH), 7.36 (d, *J* = 9.2 Hz, 2H, ArH), 7.28-7.26 (m, 1H, ArH), 6.98-6.97 (m, 1H, ArH), 6.81 (d, *J* = 8.4 Hz, 1H, ArH), 6.80 (d, *J* = 9.2 Hz, 2H, ArH), 3.80 (s, 3H, OCH_3), 3.71-3.56 (m, 2H, CH_2), 3.26 (d, *J* = 13.6 Hz, 1H, CH), 3.11 (d, *J* = 13.6 Hz, 1H, CH), 2.50 (s, 3H, CH_3), 1.56-1.53 (m, 2H, CH_2), 1.30-1.24 (m, 2H, CH_2), 0.88 (t, *J* = 7.2 Hz, 3H, CH_3) ppm; ^{13}C {^{1}H} NMR (100 MHz, $CDCl_3$) δ: 173.1, 161.8, 160.1, 146.0, 140.8, 139.7, 139.7, 132.7, 131.0, 130.0, 129.9, 129.0, 128.8, 128.5, 128.5, 125.2, 116.9, 113.9, 110.1, 91.3, 55.3, 51.9, 40.4, 36.5, 29.2, 21.8, 19.9, 13.6 ppm; IR (KBr) υ: 302, 2962, 2928, 2871, 2827, 2213, 1715, 1614, 1592, 1481, 1441, 1422, 1370, 1292, 1185, 1173, 1088, 992, 884, 811, 756, 695, 656, 603 cm^{-1}; MS (*m/z*): HRMS (ESI-TOF) Calcd. for $C_{31}H_{29}ClN_4O_4SNa$ ($[M+Na]^+$): 611.1490, Found: 611.1483.

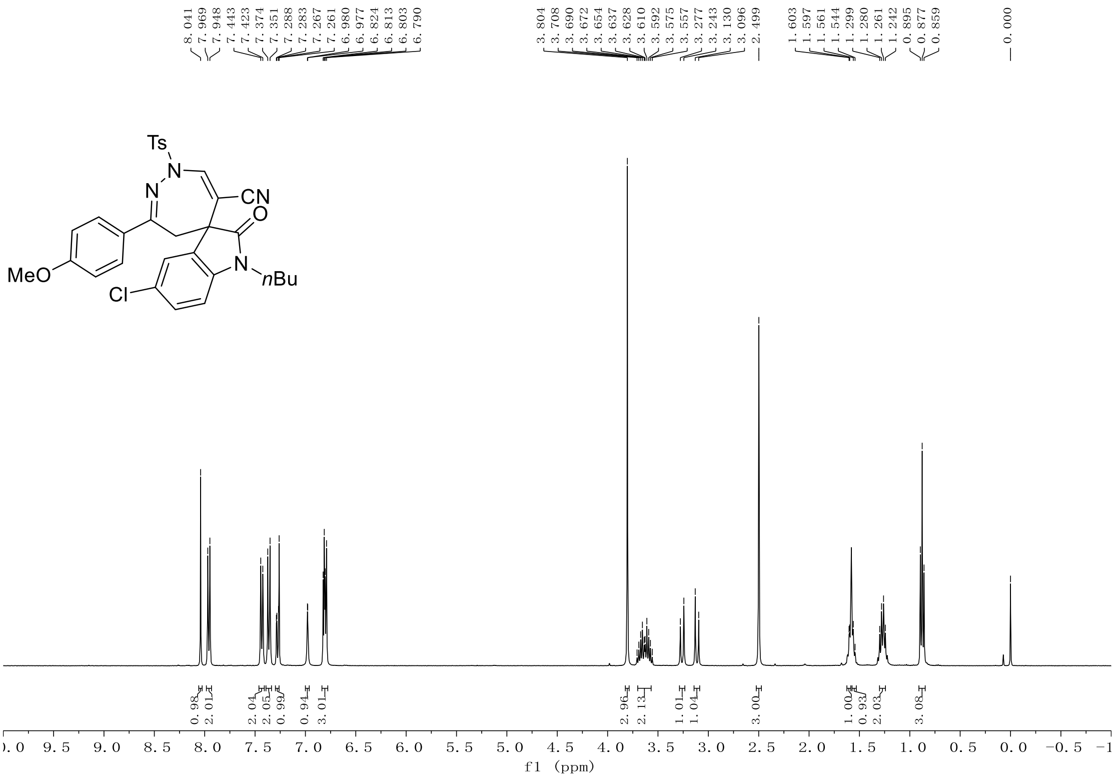

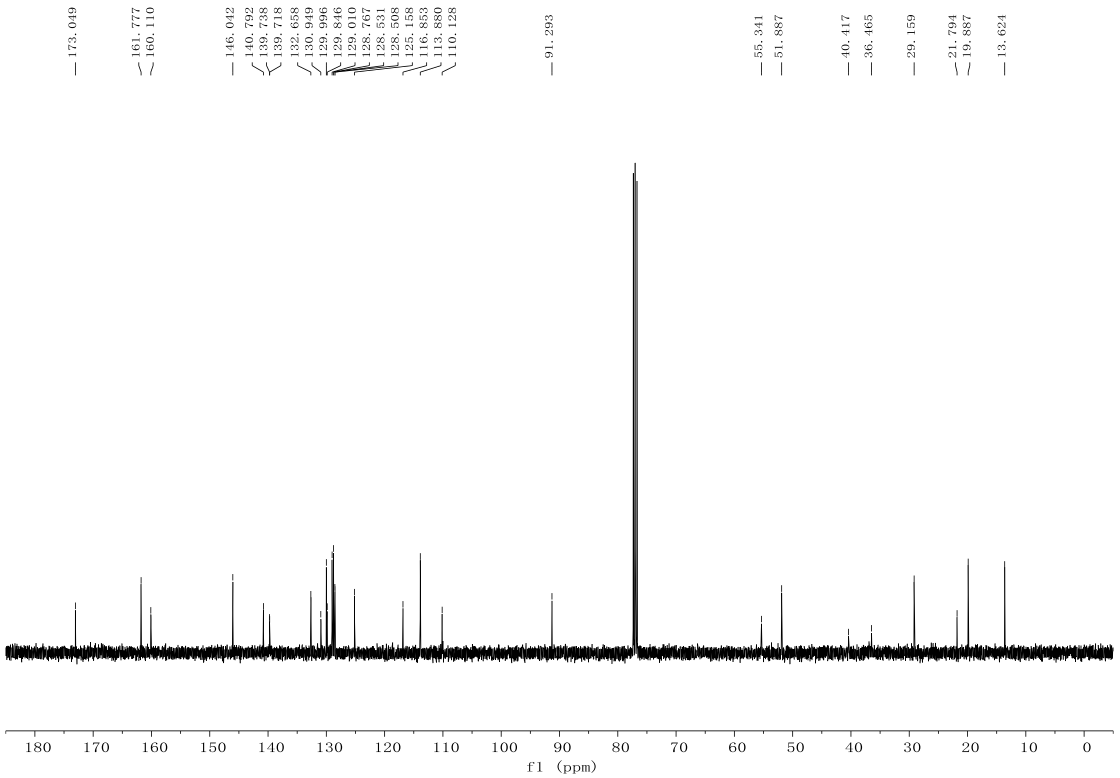
173.049
161.777
160.110
146.042
140.792
139.738
139.718
132.658
130.949
129.996
129.846
129.010
128.767
128.531
128.508
125.158
116.853
113.880
110.128
91.293
55.341
51.887
40.417
36.465
29.159
21.794
19.887
13.624
180 170 160 150 140 130 120 110 100 90 80 70 60 50 40 30 20 10 0
f1 (ppm)

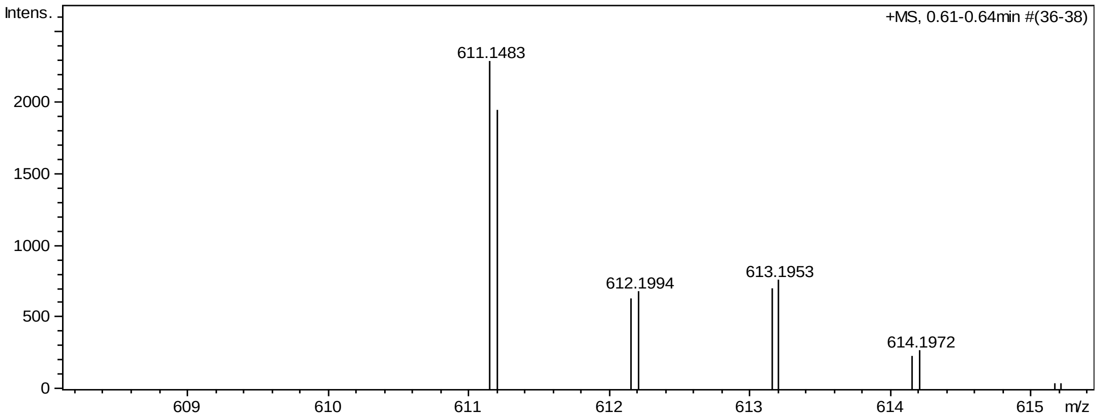
Intens.
+MS, 0.61-0.64min #(36-38)
611.1483
612.1994
613.1953
614.1972
0
500
1000
1500
2000
609
610
611
612
613
614
615
m/z

1-Butyl-3'-(4-chlorophenyl)-5-methyl-2-oxo-1'-tosyl-1',4'-dihydrospiro[indoline-3,5'-[1,2]diazepine]-6'-carbonitrile (7g): white solid, 63%, m.p. 214-217 ºC; ^{1}H NMR (400 MHz, $CDCl_3$) δ: 8.04 (s, 1H, C=CH-N), 7.95 (d, *J* = 8.4 Hz, 2H, ArH), 7.42 (d, *J* = 8.0 Hz, 2H, ArH), 7.36 (d, *J* = 8.8 Hz, 2H, ArH), 7.27 (d, *J* = 7.6 Hz, 1H, ArH), 7.13 (d, *J* = 8.0 Hz, 1H, ArH), 6.91 (s, 1H, ArH), 6.78 (d, *J* = 8.4 Hz, 1H, ArH), 3.67-3.55 (m, 2H, CH_2), 3.18 (d, *J* = 14.0 Hz, 1H, CH), 3.14 (d, *J* = 14.0 Hz, 1H, CH), 2.50 (s, 3H, CH_3), 2.26 (s, 3H, CH_3), 1.60-1.53 (m, 2H, CH_2), 1.27-1.18 (m, 2H, CH_2), 0.85 (t, *J* = 7.6 Hz, 3H, CH_3) ppm; ^{13}C {^{1}H} NMR (100 MHz, $CDCl_3$) δ: 173.3, 159.5, 146.1, 139.6, 138.9, 138.9, 136.8, 135.0, 133.1, 132.6, 130.4, 130.0, 129.7, 129.0, 128.6, 128.5, 125.1, 117.0, 109.0, 927, 52.0, 40.3, 37.1, 29.3, 21.8, 21.0, 19.9, 13.6 ppm; IR (KBr) υ: 3061, 3057, 2912, 2210, 1710, 1613, 1591, 1488, 1450, 1433, 1375, 1342, 1291, 1263, 1231, 1211, 1183, 1168, 1083, 1010, 985, 882, 833, 823, 810, 767, 722, 701, 683, 655 cm^{-1}; MS (*m/z*): HRMS (ESI-TOF) Calcd. for $C_{31}H_{29}ClN_4O_3SNa$ ($[M+Na]^+$): 595.1541, Found: 595.1541.

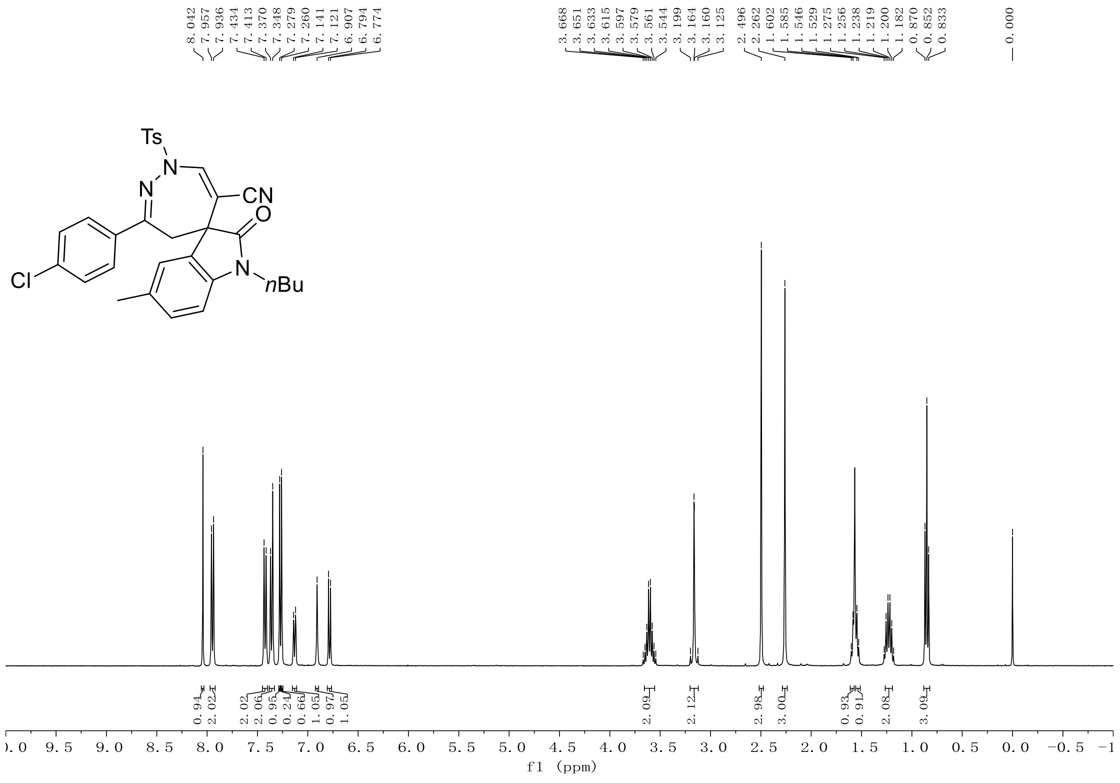

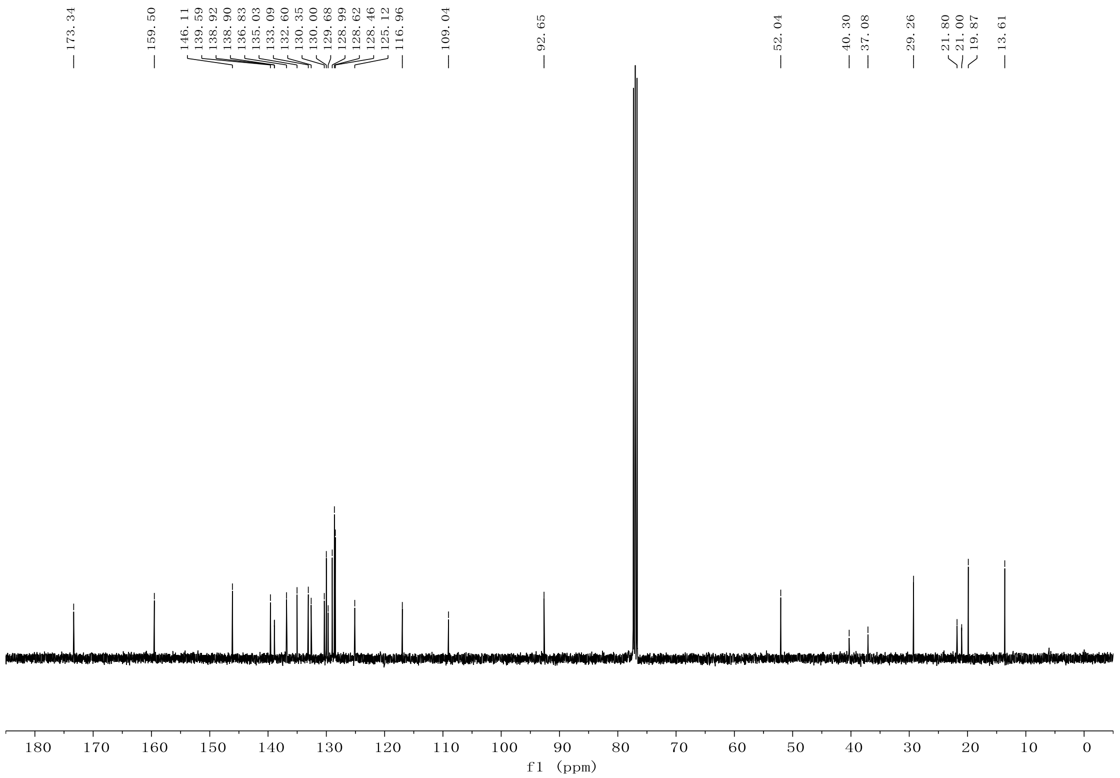

173.34
159.50
146.11
139.59
138.92
138.90
136.83
135.03
133.09
132.60
130.35
130.00
129.68
128.99
128.62
128.46
125.12
116.96
109.04
92.65
52.04
40.30
37.08
29.26
21.80
21.00
19.87
13.61
f1 (ppm)

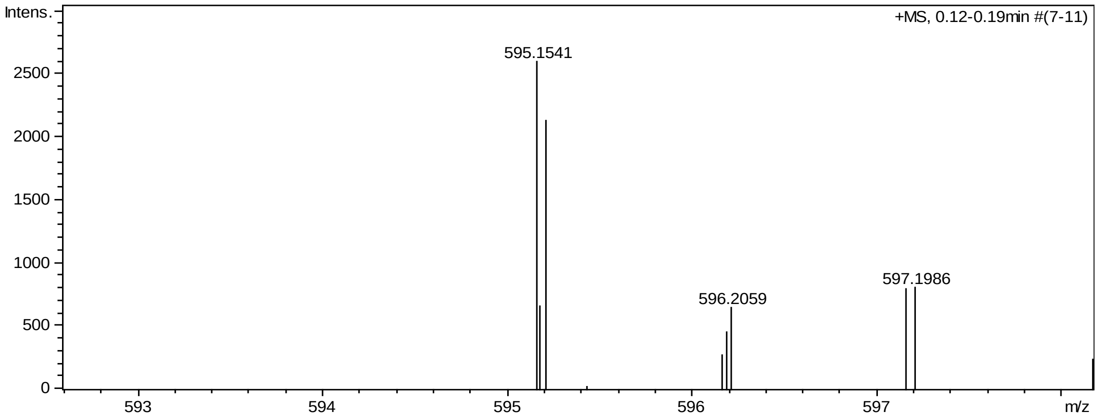

Intens.
+MS, 0.12-0.19min #(7-11)
595.1541
596.2059
597.1986
m/z

1-Butyl-5-methyl-2-oxo-3'-(p-tolyl)-1'-tosyl-1',4'-dihydrospiro[indoline-3,5'-[1,2]diazepine]-6'-carbonitrile (7h): white solid, 58%, m.p. 185-188 ºC; ^{1}H NMR (400 MHz, $CDCl_3$) δ: 8.04 (s, 1H, C=CH-N), 7.96 (d, *J* = 8.4 Hz, 2H, ArH), 7.41 (d, *J* = 8.0 Hz, 2H, ArH), 7.31 (d, *J* = 8.4 Hz, 2H, ArH), 7.11-7.08 (m, 3H, ArH), 6.87 (s, 1H, ArH), 6.77 (d, *J* = 8.0 Hz, 1H, ArH), 3.68-3.55 (m, 2H, CH_2), 3.22 (d, *J* = 14.0 Hz, 1H, CH), 3.14 (d, *J* = 14.0 Hz, 1H, CH), 2.49 (s, 3H, CH_3), 2.33 (s, 3H, CH_3), 2.22 (s, 3H, CH_3), 1.61-1.54 (m, 2H, CH_2), 1.28-1.22 (m, 2H, CH_2), 0.86 (t, *J* = 7.6 Hz, 3H, CH_3) ppm; ^{13}C {^{1}H} NMR (100 MHz, $CDCl_3$) δ: 173.4, 160.8, 145.9, 141.0, 139.7, 139.2, 133.7, 132.9, 132.8, 130.1, 129.9, 129.7, 129.1, 129.0, 127.1, 125.3, 117.1, 108.9, 92.5, 52.0, 40.2, 37.0, 29.3, 21.8, 21.3, 20.9, 19.9, 13.6 ppm; IR (KBr) υ: 3061, 3057, 2952, 2926, 2866, 2204, 1710, 1613, 1595, 1475, 1438, 1414, 1370, 1291, 1182, 1168, 1102, 1082, 988, 878, 810, 752, 692, 658, 600 cm^{-1}; MS (*m/z*): HRMS (ESI-TOF) Calcd. for $C_{32}H_{32}N_4O_3SNa$ ($[M+Na]^+$): 575.2087, Found: 575.2084.

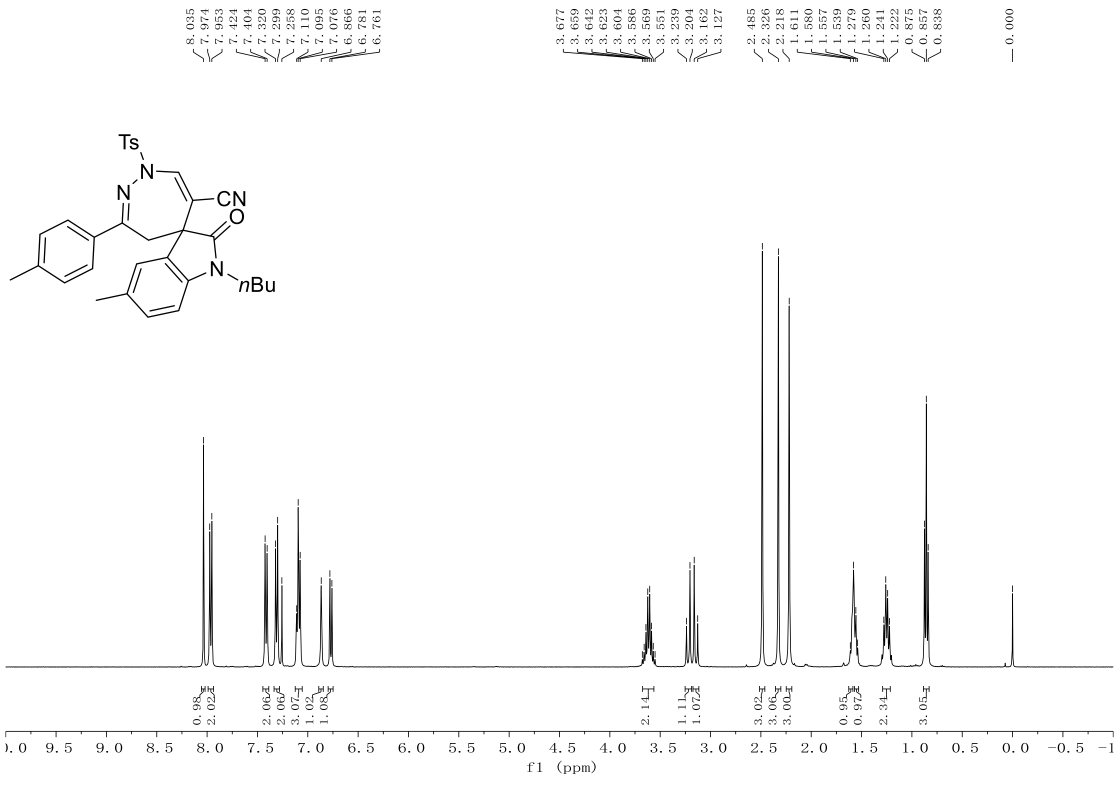

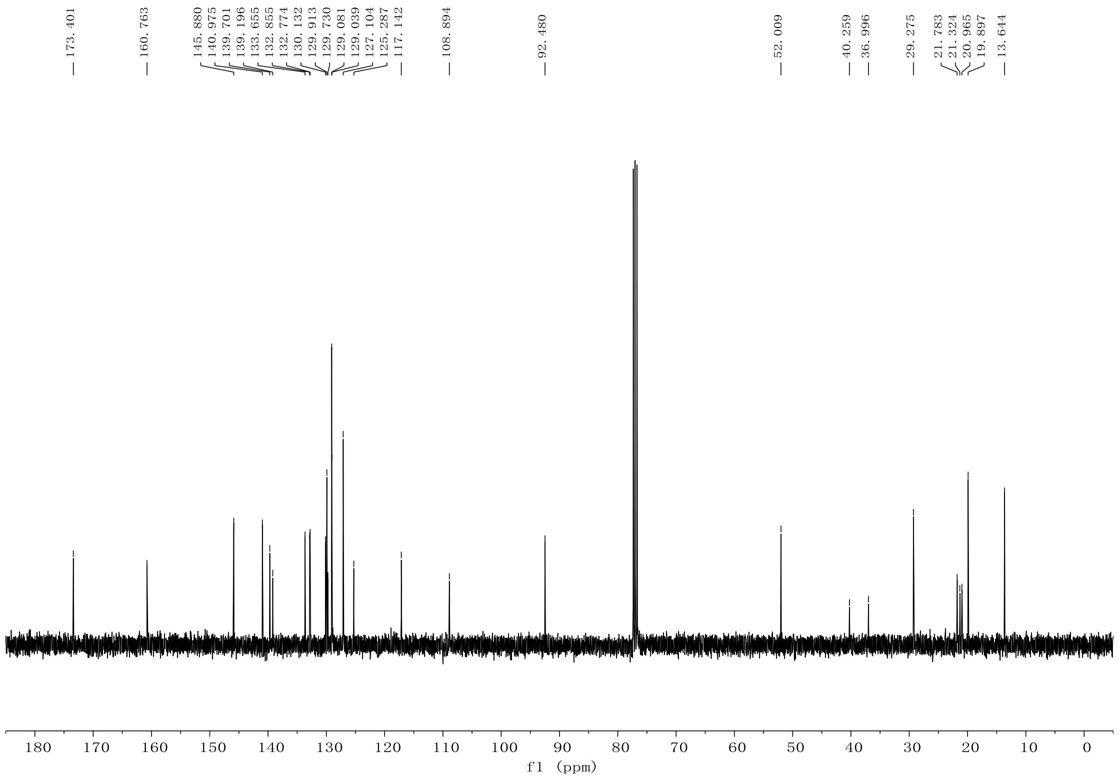
173.401
160.763
145.880
140.975
139.701
139.196
133.655
132.855
132.774
130.132
129.913
129.730
129.081
129.039
127.104
125.287
117.142
108.894
92.480
52.009
40.259
36.996
29.275
21.783
21.324
20.965
19.897
13.644
f1 (ppm)

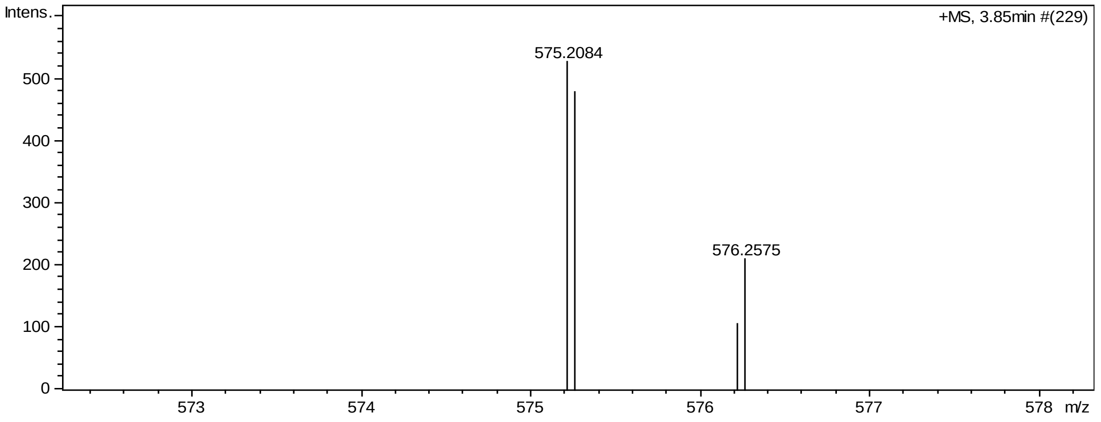
Intens.
+MS, 3.85min #(229)
575.2084
576.2575
m/z

1-Butyl-5-fluoro-3'-(4-methoxyphenyl)-2-oxo-1'-tosyl-1',4'-dihydrospiro[indoline-3,5'-[1,2]diazepine]-6'-carbonitrile (7i): white solid, 82%, m.p. 263-265 ºC; ^{1}H NMR (400 MHz, $CDCl_3$) δ: 8.04 (s, 1H, C=CH-N), 7.96 (d, *J* = 8.4 Hz, 2H, ArH), 7.42 (d, *J* = 8.0 Hz, 2H, ArH), 7.36 (d, *J* = 9.2 Hz, 2H, ArH), 7.03-6.98 (m, 1H, ArH), 6.84-6.82 (m, 1H, ArH), 6.79 (d, *J* = 9.2 Hz, 2H, ArH), 6.75-6.72 (m, 1H, ArH), 3.80 (s, 3H, OCH_3), 3.71-3.59 (m, 2H, CH_2), 3.28 (d, *J* = 14.0 Hz, 1H, CH), 3.11 (d, *J* = 13.6 Hz, 1H, CH), 2.50 (s, 3H, CH_3), 1.62-1.55 (m, 2H, CH_2), 1.32-1.26 (m, 2H, CH_2), 0.89 (t, *J* = 7.2 Hz, 3H, CH_3) ppm; ^{13}C {^{1}H} NMR (100 MHz, $CDCl_3$) δ: 178.9, 175.4, 173.2, 165.4, 161.8, 160.1, 159,1 (d, *J* = 242 Hz), 146.0, 139.7 (d, *J* = 3.2 Hz), 138.2, 132.7, 130.8 (d, *J* = 7.0 Hz), 130.0, 129.9, 128.8, 128.5, 116.9, 116.3 (d, *J* = 23 Hz), 113.9, 109.9, 109.8, 109.8, 91.4, 55.4, 52.1, 40.4, 36.5, 29.2, 21.8, 19.9, 13.6 ppm; IR (KBr) υ: 3061, 2957, 2942, 2930, 2871, 2205, 1720, 1612, 1599, 1488, 1449, 1422, 1375, 1299, 1182, 1114, 1094, 998, 759, 698, 658, 606 cm^{-1}; MS (*m/z*): HRMS (ESI-TOF) Calcd. for $C_{31}H_{29}FN_4O_4SNa$ ([M+Na]$^+$): 595.1786, Found: 595.1785.

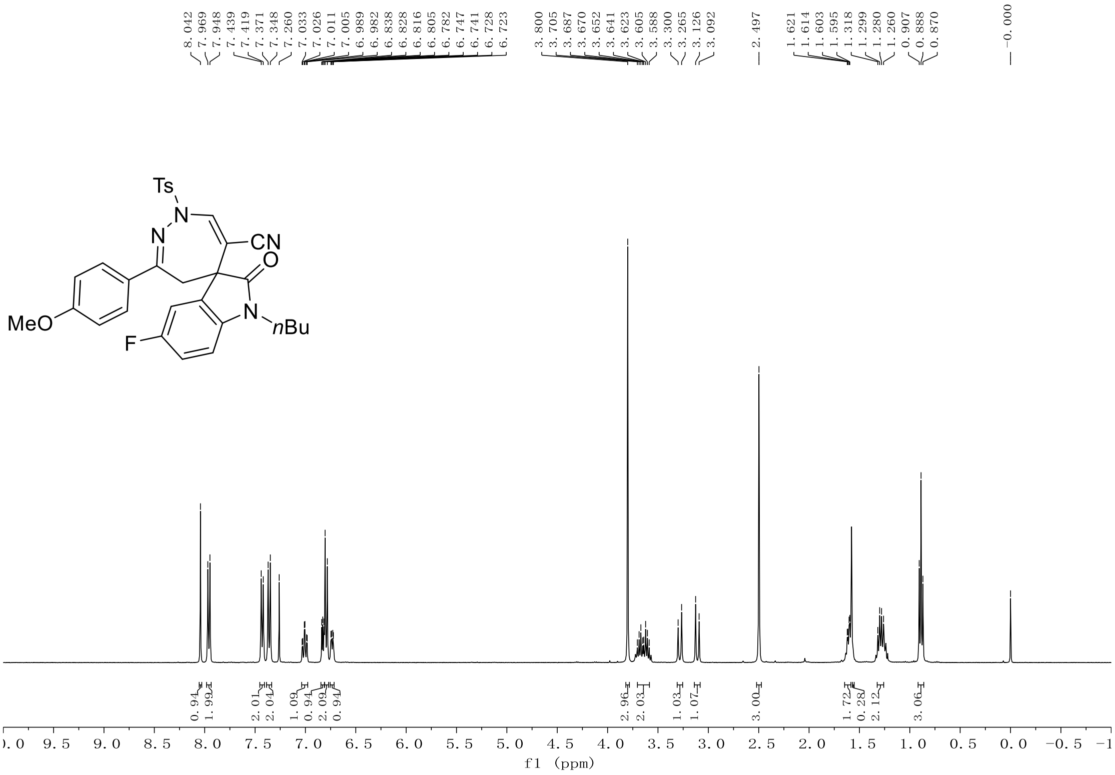

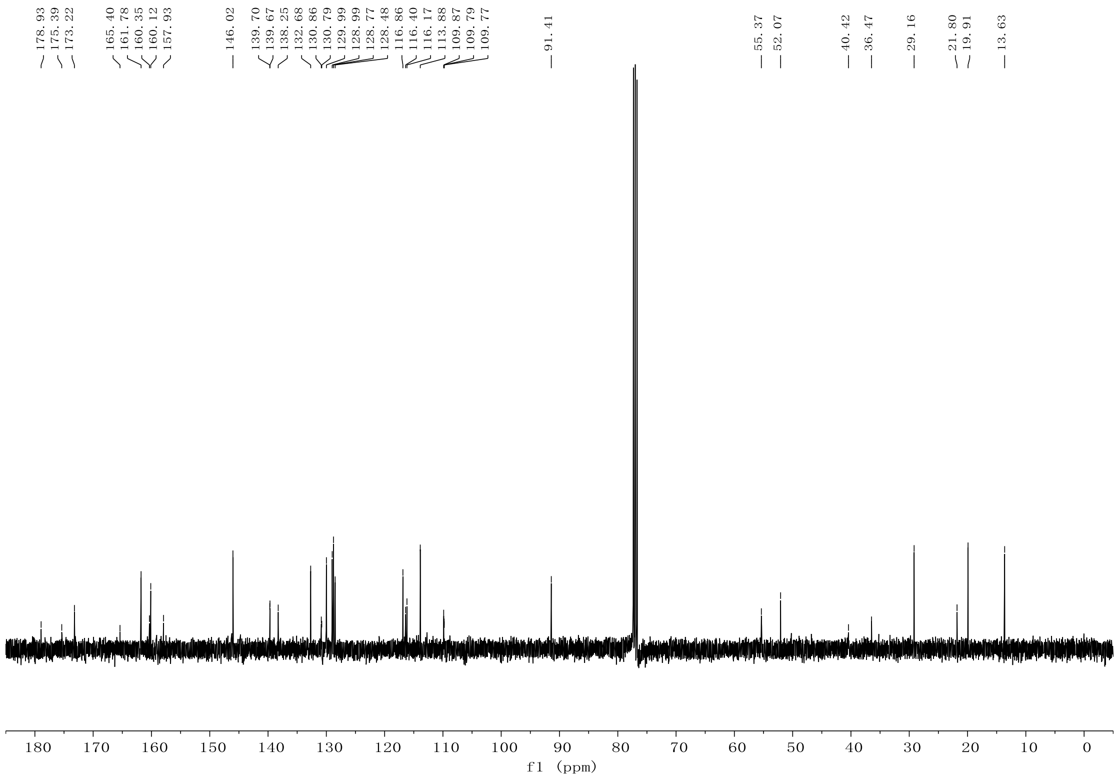
178.93
175.39
173.22
165.40
161.78
160.35
160.12
157.93
146.02
139.70
139.67
138.25
132.68
130.86
130.79
129.99
128.99
128.77
128.48
116.86
116.40
116.17
113.88
109.87
109.79
109.77
91.41
55.37
52.07
40.42
36.47
29.16
21.80
19.91
13.63
180 170 160 150 140 130 120 110 100 90 80 70 60 50 40 30 20 10 0
f1 (ppm)

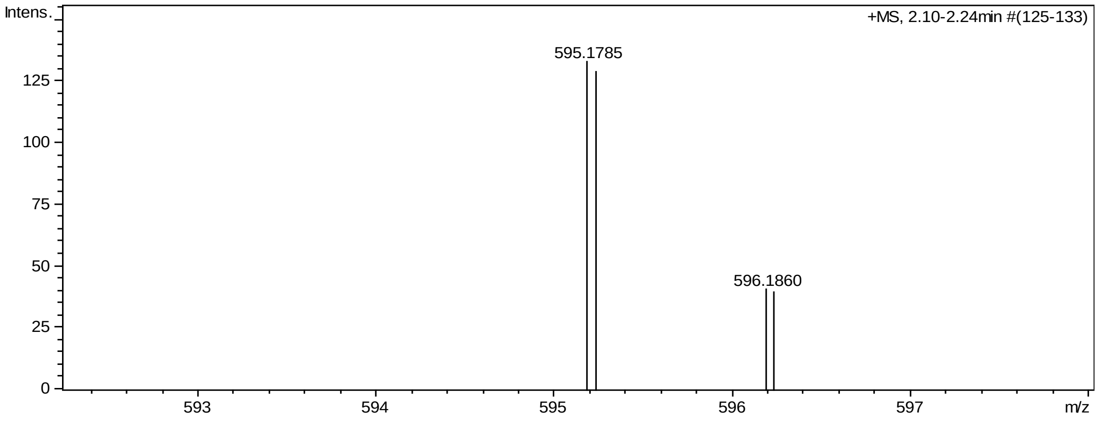
Intens.
+MS, 2.10-2.24min #(125-133)
595.1785
596.1860
125
100
75
50
25
0
593
594
595
596
597
m/z

1-Butyl-3'-(4-methoxyphenyl)-5-methyl-2-oxo-1'-tosyl-1',4'-dihydrospiro[indoline-3,5'-[1,2]diazepine]-6'-carbonitrile (7j): white solid, 62%, m.p. 189-192 ºC; ^{1}H NMR (400 MHz, $CDCl_3$) δ: 8.03 (s, 1H, C=CH-N), 7.97 (d, *J* = 8.0 Hz, 2H, ArH), 7.42 (d, *J* = 8.4 Hz, 2H, ArH), 7.37 (d, *J* = 8.8 Hz, 2H, ArH), 7.10 (d, *J* = 7.6 Hz, 1H, ArH), 6.86 (s, 1H, ArH), 6.80-6.76 (m, 3H, ArH), 3.80 (s, 3H, OCH_3), 3.67-3.56 (m, 2H, CH_2), 3.22 (d, *J* = 13.6 Hz, 1H, CH), 3.13 (d, *J* = 14.0 Hz, 1H, CH), 2.49 (s, 3H, CH_3), 2.21 (s, 3H, CH_3), 1.62-1.55 (m, 2H, CH_2), 1.31-1.22 (m, 2H, CH_2), 0.87 (t, *J* = 7.6 Hz, 3H, CH_3) ppm; ^{13}C {^{1}H} NMR (100 MHz, $CDCl_3$) δ: 173.4, 161.6, 160.5, 145.8, 139.7, 139.4, 132.8, 130.1, 129.9, 129.7, 129.0, 128.9, 128.8, 125.3, 117.1, 113.7, 108.9, 92.4, 55.4, 52.0, 40.3, 36.8, 29.3, 21.8, 20.9, 19.9, 13.7 ppm; IR (KBr) υ: 3057, 2941, 2930, 2852, 2201, 1715, 1601, 1592, 1477, 1442, 1421, 1371, 1299, 1182, 1110, 1090, 991, 753, 694, 654, 601 cm^{-1}; MS (*m/z*): HRMS (ESI-TOF) Calcd. for $C_{32}H_{32}N_4O_4SNa$ ($[M+Na]^+$): 591.2036, Found: 591.2039.

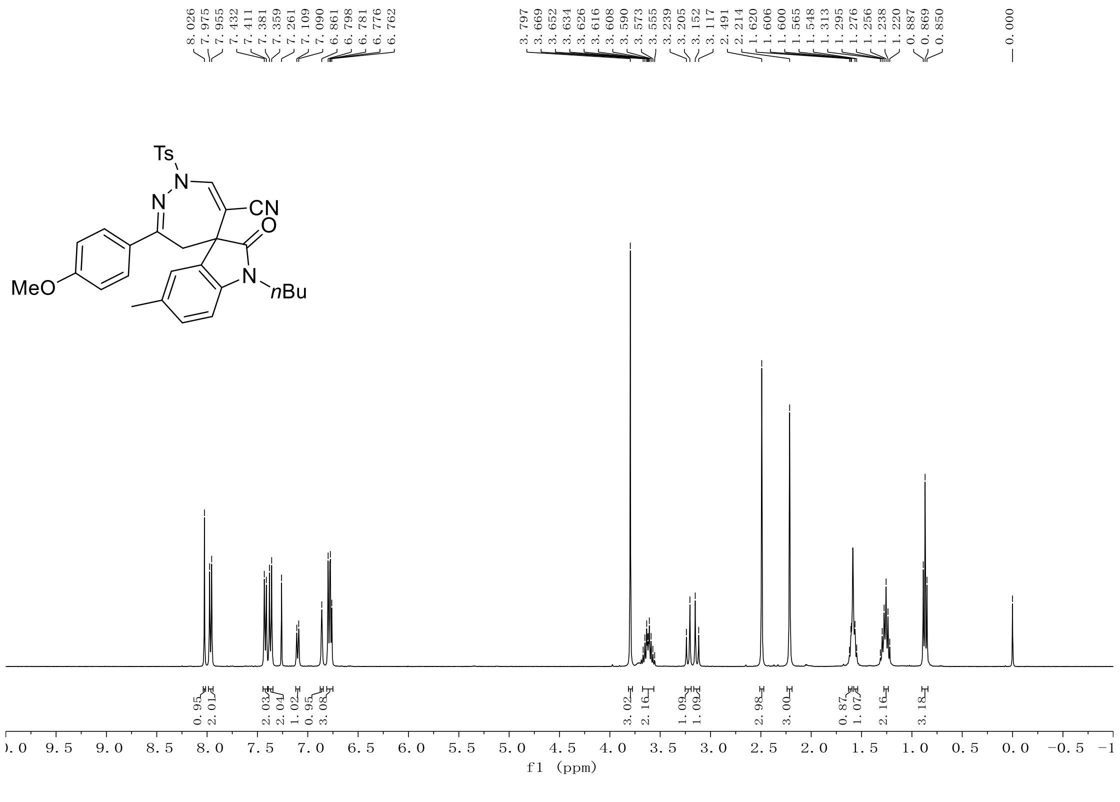

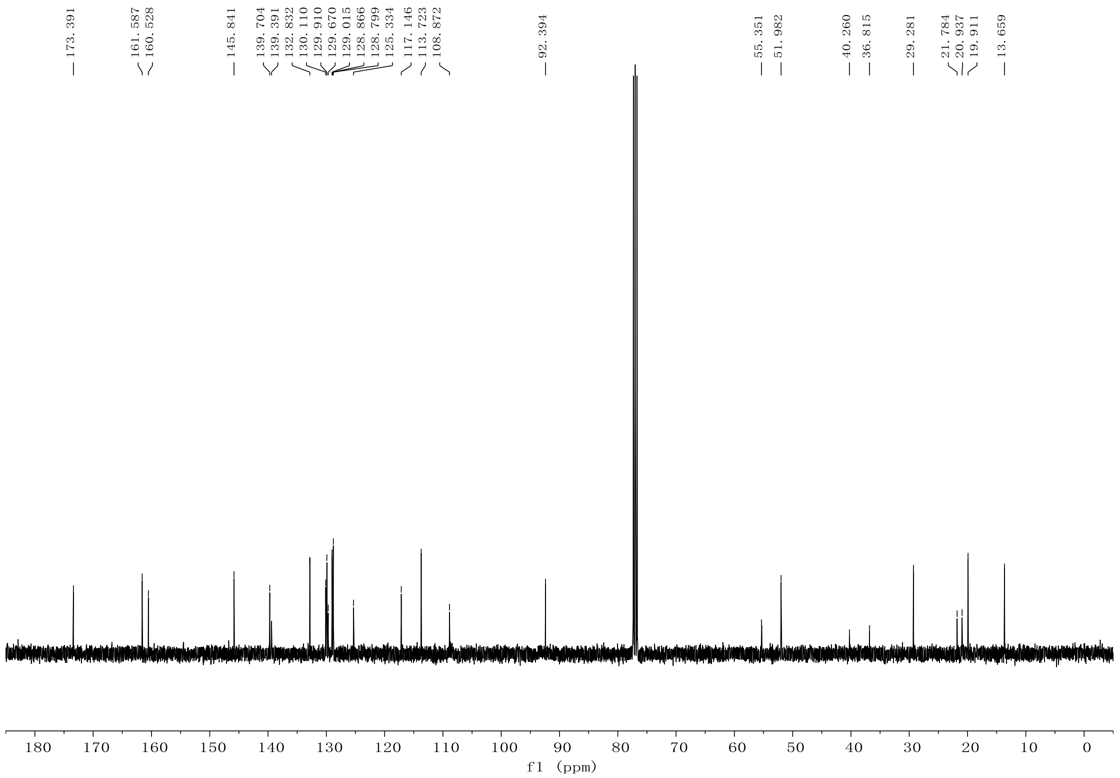
173.391
161.587
160.528
145.841
139.704
139.391
132.832
130.110
129.910
129.670
129.015
128.866
128.799
125.334
117.146
113.723
108.872
92.394
55.351
51.982
40.260
36.815
29.281
21.784
20.937
19.911
13.659
f1 (ppm)

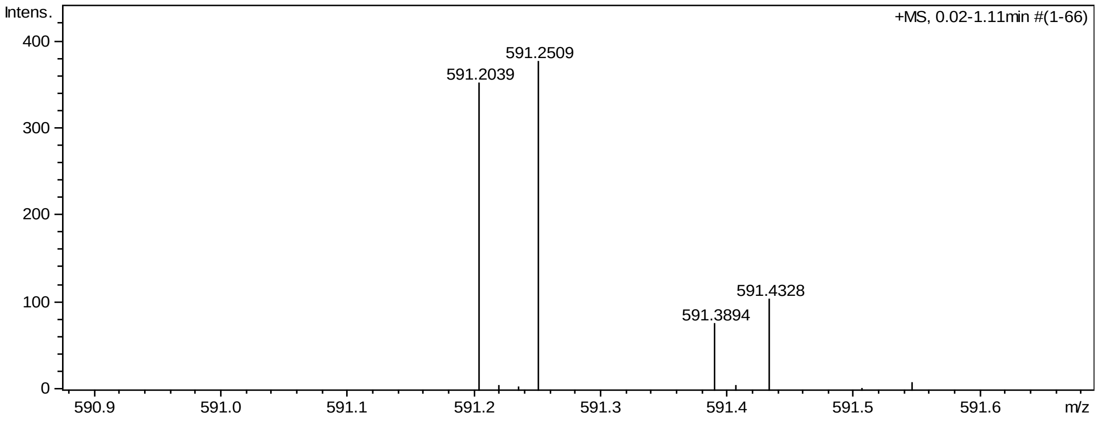
Intens.
+MS, 0.02-1.11min #(1-66)
591.2509
591.2039
591.4328
591.3894
m/z

1-Butyl-3'-(4-chlorophenyl)-5-fluoro-2-oxo-1'-tosyl-1',4'-dihydrospiro[indoline-3,5'-[1,2]diazepine]-6'-carbonitrile (7k): white solid, 65%, m.p. 242-244 ºC; ^{1}H NMR (400 MHz, $CDCl_3$) δ: 8.06 (s, 1H, C=CH-N), 7.94 (d, *J* = 8.0 Hz, 2H, ArH), 7.43 (d, *J* = 8.0 Hz, 2H, ArH), 7.35 (d, *J* = 8.8 Hz, 2H, ArH), 7.28-7.25 (m, 2H, ArH), 7.07-7.02 (m, 1H, ArH), 6.85-6.80 (m, 2H, ArH), 3.69-3.55 (m, 2H, CH_2), 3.20 (d, *J* = 14.0 Hz, 1H, CH), 3.14 (d, *J* = 14.0 Hz, 1H, CH), 2.50 (s, 3H, CH_3), 1.59-1.54 (m, 2H, CH_2), 1.27-1.21 (m, 2H, CH_2), 0.86 (t, *J* = 7.2 Hz, 3H, CH_3) ppm; ^{13}C {^{1}H} NMR (100 MHz, $CDCl_3$) δ: 173.2, 159.5 (d, *J* = 242 Hz), 158.0, 146.3, 139.2 (d, *J* = 2.6 Hz), 138.1 (d, *J* = 2.1 Hz), 137.1, 134.7, 132.4, 130.8 (d, *J* = 7.8 Hz), 130.1, 129.0, 128.8, 128.4, 116.7 (d, *J* = 23.4 Hz), 116.4, 112.6 (d, *J* = 24.6 Hz), 110 (d, *J* = 7.8 Hz), 91.6, 52.1, 40.5, 36.8, 29.1, 21.8, 19.9, 13.6 ppm; IR (KBr) υ: 3072, 2959, 2931, 2921, 2872, 2216, 1719, 1623, 1599, 1482, 1457, 1447, 1409, 1370, 1295, 1273, 1239, 1219, 1189, 1177, 1089, 1009, 999, 889, 846, 831, 812, 782, 737, 709, 692, 672 cm^{-1}; MS (*m/z*): HRMS (ESI-TOF) Calcd. for $C_{30}H_{26}ClFN_4O_3SNa$ ([M+Na]$^+$): 599.1290, Found: 599.1292.

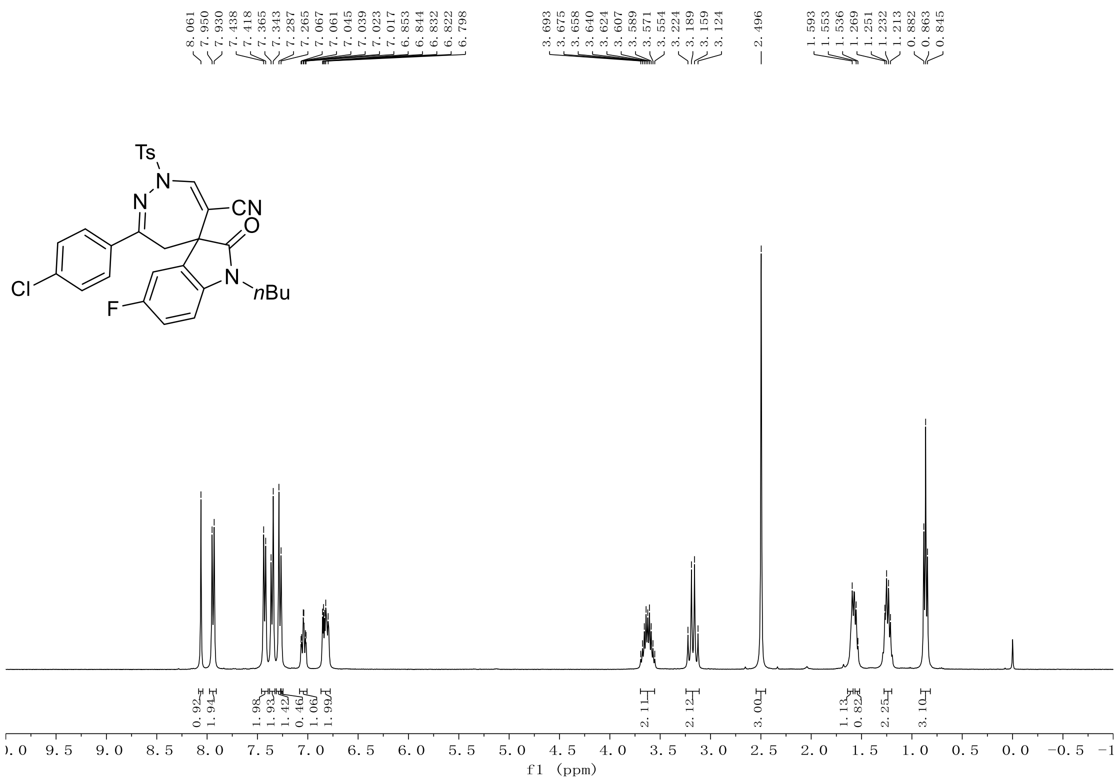

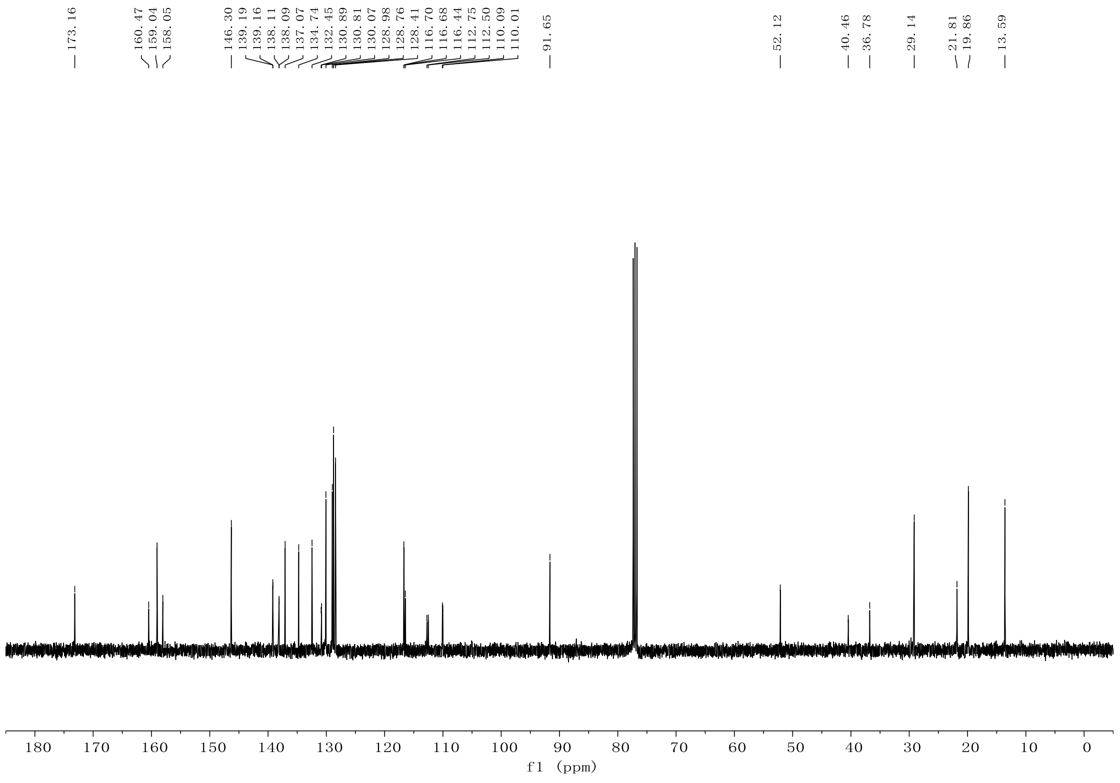
173.16
160.47
159.04
158.05
146.30
139.19
139.16
138.11
138.09
137.07
134.74
132.45
130.89
130.81
130.07
128.98
128.76
128.41
116.70
116.68
116.44
112.75
112.50
110.09
110.01
91.65
52.12
40.46
36.78
29.14
21.81
19.86
13.59
180 170 160 150 140 130 120 110 100 90 80 70 60 50 40 30 20 10 0
f1 (ppm)

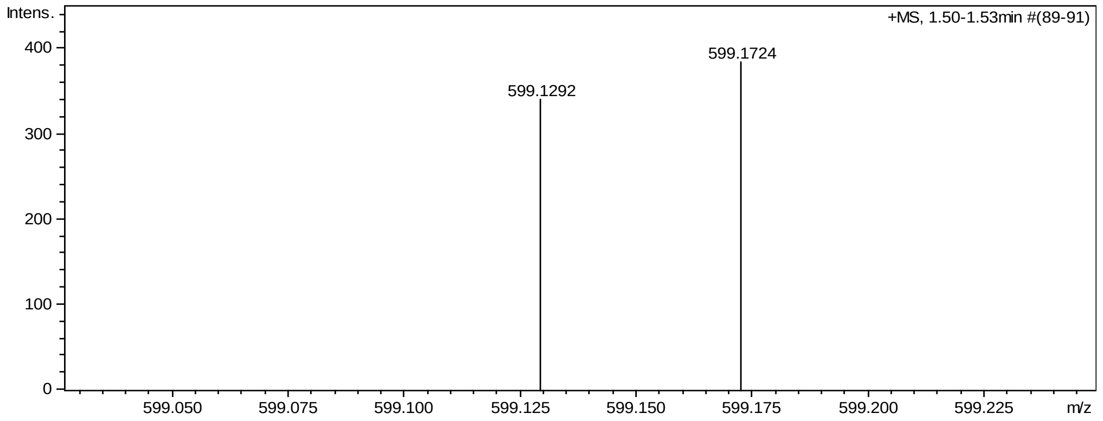
Intens.
+MS, 1.50-1.53min #(89-91)
599.1724
599.1292
400
300
200
100
0
599.050 599.075 599.100 599.125 599.150 599.175 599.200 599.225 m/z

1-Benzyl-2-oxo-3'-phenyl-1'-tosyl-1',4'-dihydrospiro[indoline-3,5'-[1,2]diazepine]-6'-carbonitrile (7l): white solid, 75%, m.p. 208-211 ºC; ^{1}H NMR (400 MHz, $CDCl_3$) δ: 8.11 (s, 1H, C=CH-N), 7.98 (d, *J* = 8.4 Hz, 2H, ArH), 7.43-7.39 (m, 3H, ArH), 7.37 (d, *J* = 6.8 Hz, 1H, ArH), 7.29 (d, *J* = 7.6 Hz, 2H, ArH), 7.26-7.25 (m, 3H, ArH), 7.21 (d, *J* = 7.2 Hz, 1H, ArH), 7.17-7.15 (m, 2H, ArH), 7.08 (d, *J* = 7.6 Hz, 1H, ArH),7.01-6.97 (m, 1H, ArH), 6.79 (d, *J* = 7.2 Hz, 1H, ArH), 4.87 (d, *J* = 15.6 Hz, 1H, CH), 4.77 (d, *J* = 15.6 Hz, 1H, CH), 3.29 (d, *J* = 14.0 Hz, 1H, CH), 3.22 (d, *J* = 14.0 Hz, 1H, CH), 2.49 (s, 3H, CH_3) ppm; ^{13}C {^{1}H} NMR (100 MHz, $CDCl_3$) δ: 173.7, 160.3, 146.0, 141.8, 139.3, 139.2, 136.4, 135.0, 132.7, 130.6, 130.0, 130.0, 129.6, 129.0, 128.8, 128.4, 127.8, 127.3, 127.3, 124.4, 123.5, 117.2, 110.0, 109.9, 91.9, 52.1, 44.3, 37.5, 21.8 ppm; IR (KBr) υ: 3056, 2926, 2216, 1715, 1611, 1486, 1466, 1447, 1367, 1295, 1259, 1188, 1173, 1089, 1019, 997, 868, 802, 762, 753, 694, 665, 656, 638 cm^{-1}; MS (*m*/*z*): HRMS (ESI-TOF) Calcd. for $C_{33}H_{26}N_4O_3SNa$ ([M+Na]$^+$): 581.1618, Found: 581.1617.

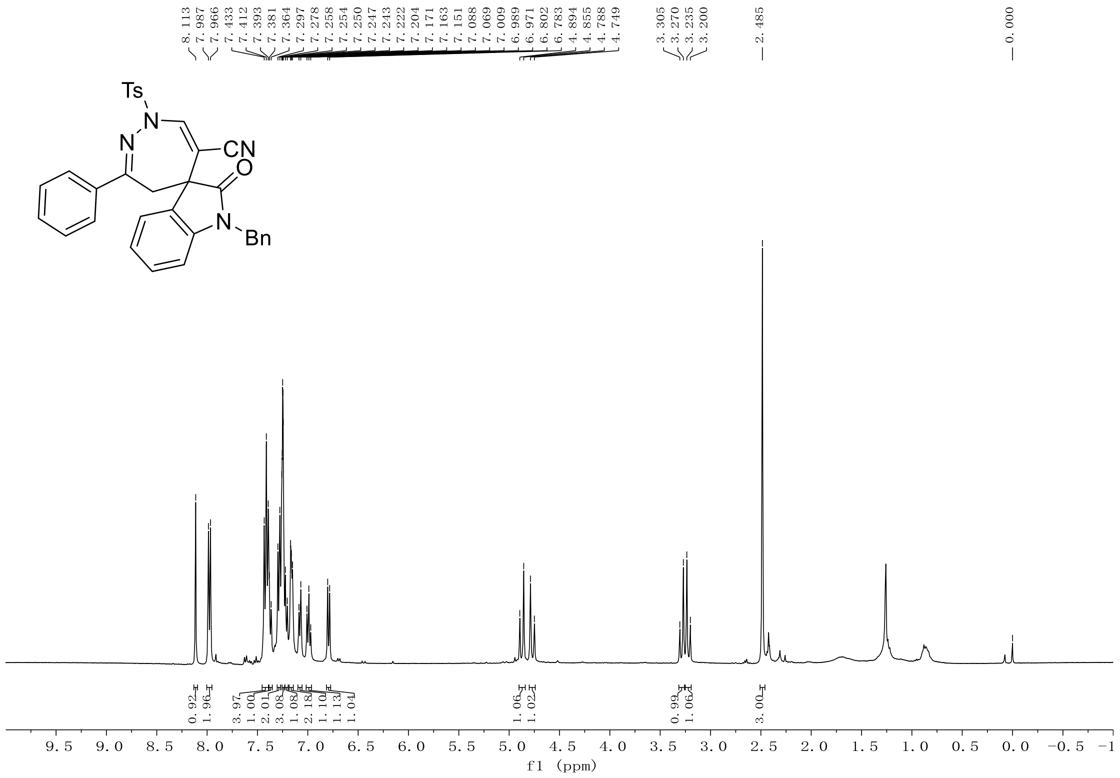

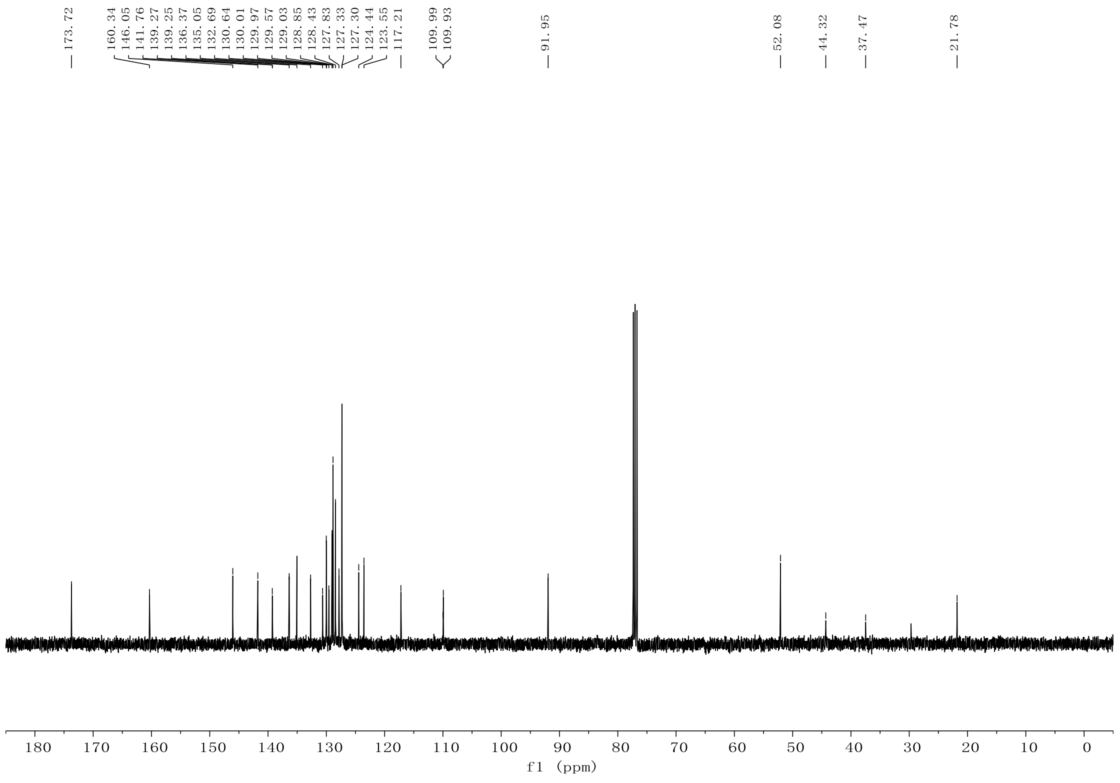

173.72
160.34
146.05
141.76
139.27
139.25
136.37
135.05
132.69
130.64
130.01
129.97
129.57
129.03
128.85
128.43
127.83
127.33
127.30
124.44
123.55
117.21
109.99
109.93
91.95
52.08
44.32
37.47
21.78
180 170 160 150 140 130 120 110 100 90 80 70 60 50 40 30 20 10 0
f1 (ppm)

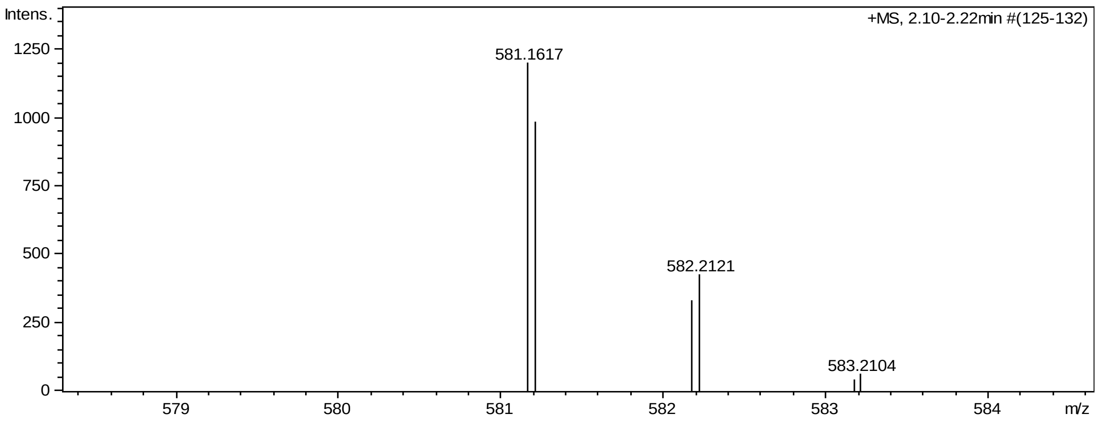

Intens.
+MS, 2.10-2.22min #(125-132)
1250
1000
750
500
250
0
581.1617
582.2121
583.2104
579 580 581 582 583 584
m/z

1-Butyl-3'-(3-chlorophenyl)-5-methyl-2-oxo-1'-tosyl-1',4'-dihydrospiro[indoline-3,5'-[1,2]diazepine]-6'-carbonitrile (7m): white solid, 71%, m.p. 199-202 ºC; ^{1}H NMR (600 MHz, $CDCl_3$) δ: 8.06 (s, 1H, C=CH-N), 7.97-7.96 (m, 2H, ArH), 7.45-7.44 (m, 2H, ArH), 7.40 (s, 1H, ArH), 7.36-7.34 (m, 1H, ArH), 7.32-7.29 (m, 1H, ArH), 7.24-7.21 (m, 1H, ArH), 7.15-7.13 (m, 1H, ArH), 6.93 (s, 1H, ArH), 6.81-6.78 (m, 1H, ArH), 3.63-3.60 (m, 2H, CH_2), 3.18-3.12 (m, 2H, CH_2), 2.51 (s, 3H, CH_3), 2.28 (s, 3H, CH_3), 1.60-1.52 (m, 2H, CH_2), 1.26-1.22 (m, 2H, CH_2), 0.87-0.84 (m, 3H, CH_3) ppm; ^{13}C NMR (151 MHz, $CDCl_3$) δ: 173.4, 158.9, 146.3, 139.6, 138.8, 138.4, 134.5, 133.2, 132.6, 130.5, 130.4, 130.1, 129.8, 129.6, 129.1, 127.4, 125.3, 125.2, 117.0, 109.1, 92.7, 52.2, 40.4, 37.2, 29.3, 21.9, 21.1, 19.9, 13.7 ppm; IR (KBr) υ: 3064, 3052, 2922, 2213, 1715, 1611, 1597, 1489, 1445, 1428, 1375, 1341, 1281, 1258, 1222, 1207, 1181, 1162, 1075, 1009, 978, 878, 822, 762, 701, 683, 655 cm^{-1}; MS (*m*/*z*): HRMS (ESI-TOF) Calcd. for $C_{31}H_{29}ClN_4O_3SNa$ ($[M+Na]^+$): 595.1541, Found: 595.1571.

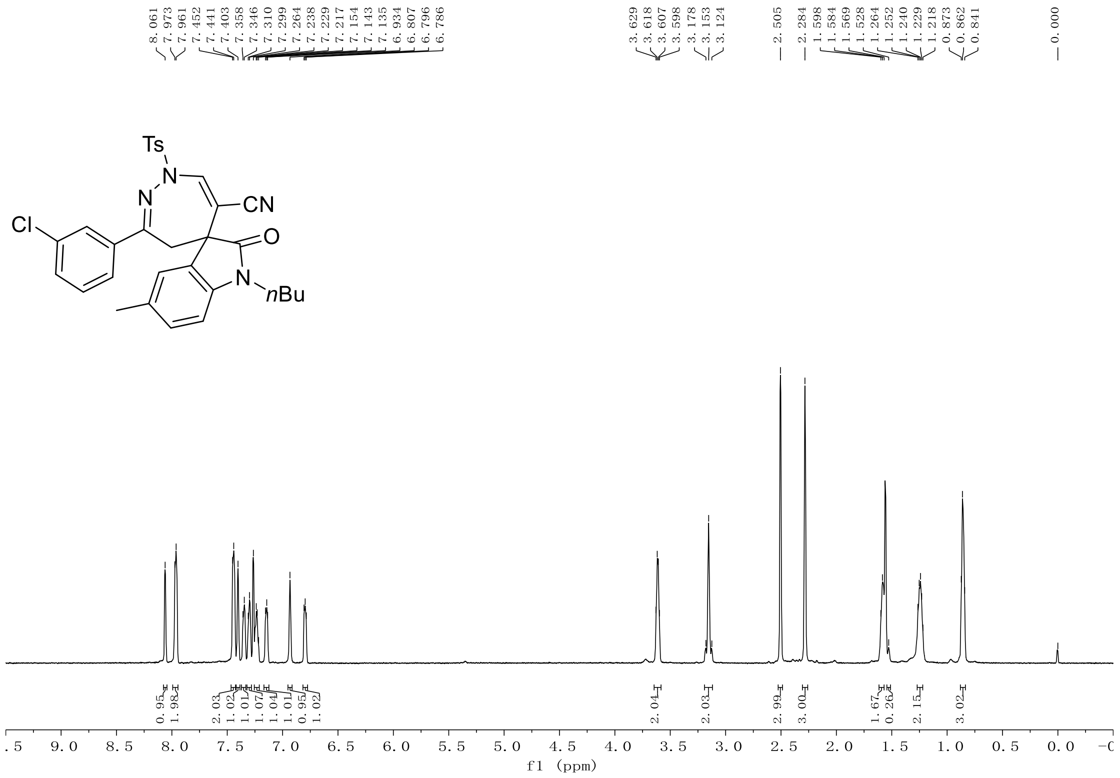

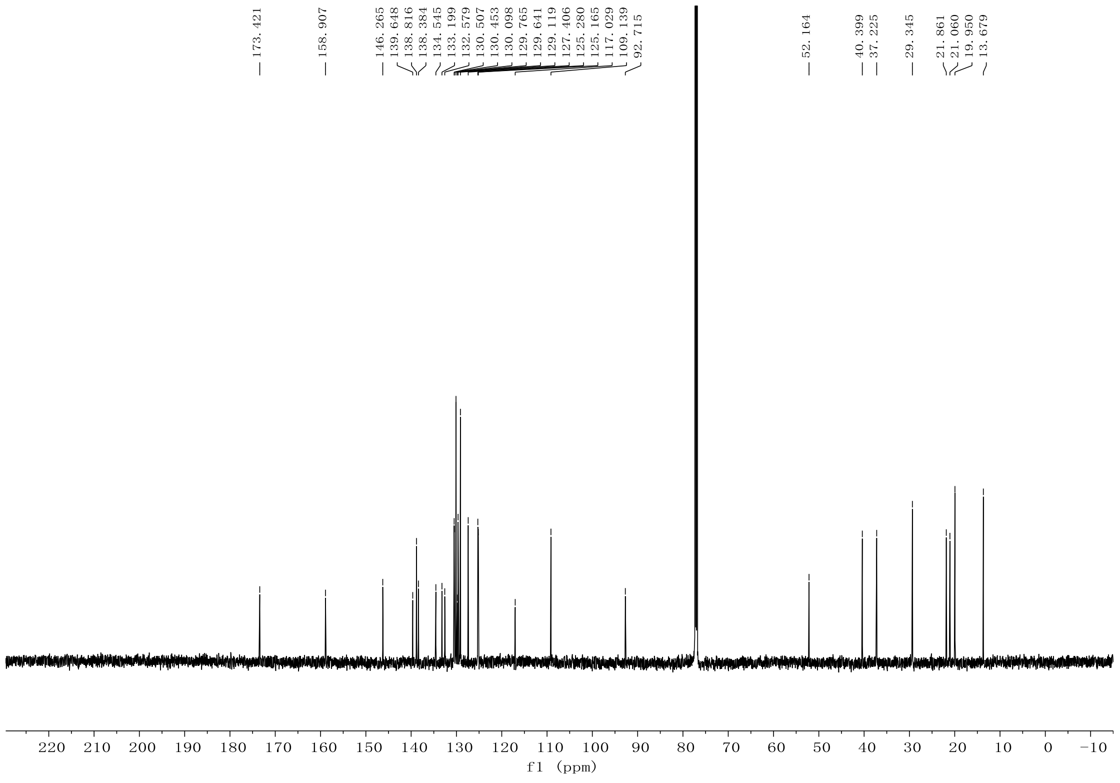
173.421
158.907
146.265
139.648
138.816
138.384
134.545
133.199
132.579
130.507
130.453
130.098
129.765
129.641
129.119
127.406
125.280
125.165
117.029
109.139
92.715
52.164
40.399
37.225
29.345
21.861
21.060
19.950
13.679
f1 (ppm)

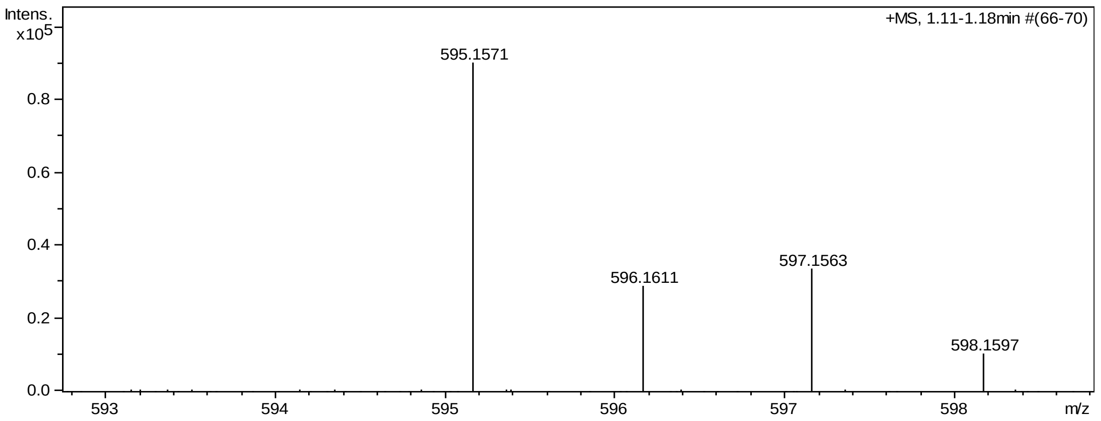
Intens.
x10^5
+MS, 1.11-1.18min #(66-70)
595.1571
596.1611
597.1563
598.1597
m/z

1-Butyl-3'-(2-chlorophenyl)-5-methyl-2-oxo-1'-tosyl-1',4'-dihydrospiro[indoline-3,5'-[1,2]diazepine]-6'-carbonitrile (7n): white solid, 64%, m.p. 185-188 ºC; ^{1}H NMR (600 MHz, $CDCl_3$) δ: 8.09 (s, 1H, C=CH-N), 7.88-7.87 (m, 2H, ArH), 7.38-7.37 (m, 2H, ArH), 7.34-7.33 (m, 1H, ArH), 7.30-7.26 (m, 2H, ArH), 7.22-7.20 (m, 1H, ArH), 7.12-7.10 (m, 1H, ArH), 7.03 (s, 1H, ArH), 6.71-6.70 (m, 1H, ArH), 3.56-3.51 (m, 2H, CH_2), 3.36 (d, J = 14.4 Hz, 1H, CH), 3.04 (d, J = 14.4 Hz, 1H, CH), 2.49 (s, 3H, CH_3), 2.34 (s, 3H, CH_3), 1.52-1.47 (m, 2H, CH_2), 1.06-1.03 (m, 2H, CH_2), 0.75-0.72 (m, 3H, CH_3) ppm; ^{13}C NMR (151 MHz, $CDCl_3$) δ 173.9, 160.5, 145.9, 139.3, 138.5, 137.5, 133.2, 132.8, 132.4, 131.9, 130.5, 130.3, 130.2, 129.9, 129.5, 129.0, 126.6, 124.9, 117.2, 108.9, 92.2, 52.4, 41.2, 40.2, 29.2, 21.8, 21.1, 19.7, 13.6 ppm; IR (KBr) υ: 3073, 3058, 2932, 2215, 1725, 1621, 1599, 1491, 1448, 1425, 1377, 1328, 1221, 1203, 1191, 1157, 1082, 1001, 973, 871, 820, 772, 711, 689, 645 cm^{-1}; MS (m/z): HRMS (ESI-TOF) Calcd. for $C_{31}H_{29}ClN_4O_3SNa$ ($[M+Na]^+$): 595.1541, Found: 595.1570.

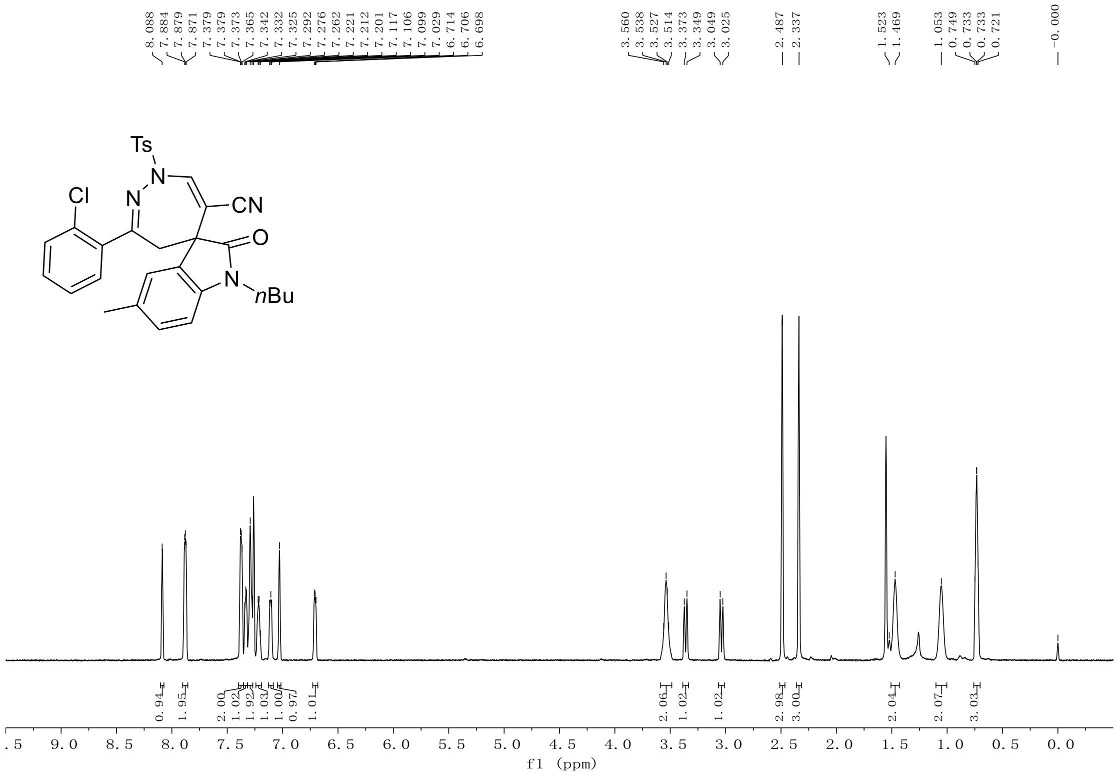

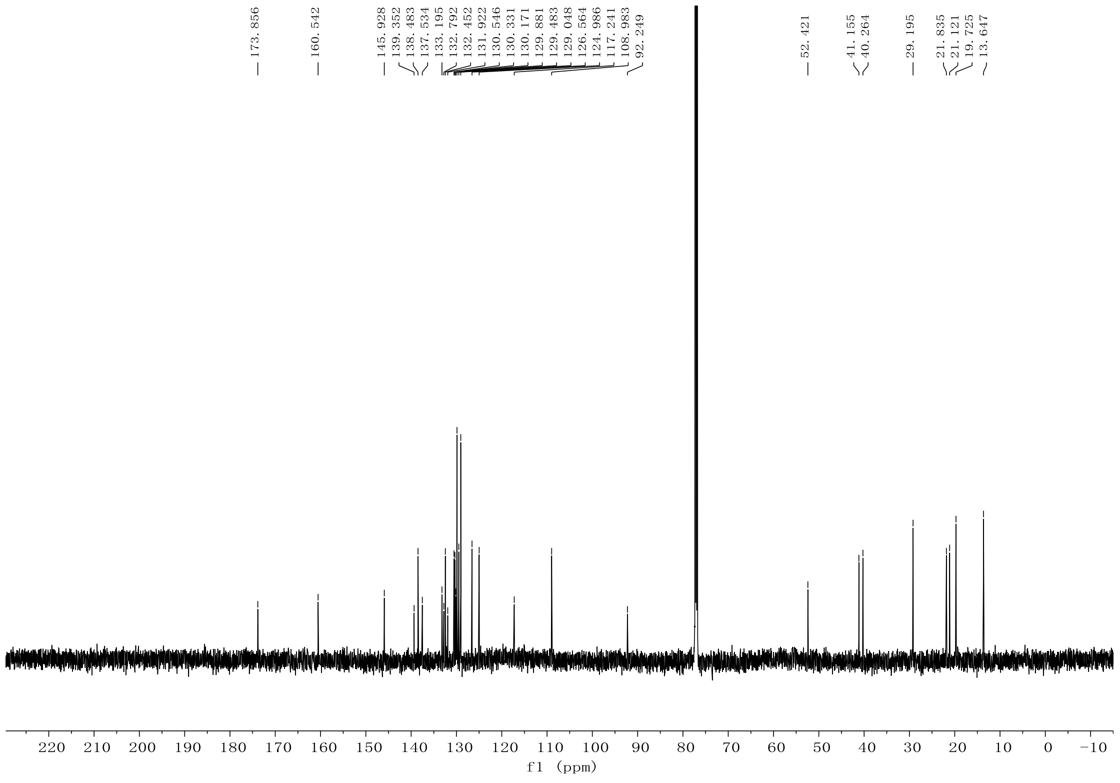
173.856
160.542
145.928
139.352
138.483
137.534
133.195
132.792
132.452
131.922
130.546
130.331
130.171
129.881
129.483
129.048
126.564
124.986
117.241
108.983
92.249
52.421
41.155
40.264
29.195
21.835
21.121
19.725
13.647
220 210 200 190 180 170 160 150 140 130 120 110 100 90 80 70 60 50 40 30 20 10 0 −10
f1 (ppm)

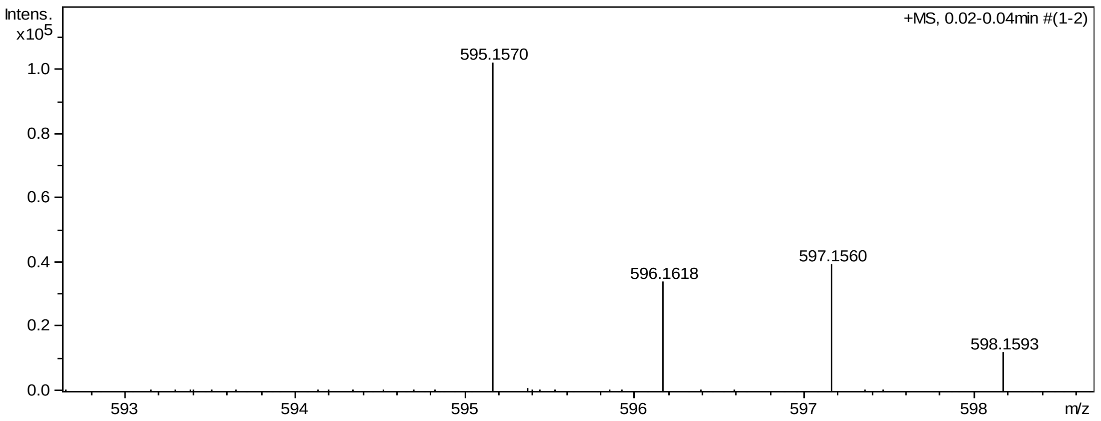
Intens.
x10^5
+MS, 0.02-0.04min #(1-2)
595.1570
596.1618
597.1560
598.1593
1.0
0.8
0.6
0.4
0.2
0.0
593
594
595
596
597
598
m/z
